# Oxidative stress and antioxidants in breast cancer: a double-edged sword

**DOI:** 10.3389/fonc.2026.1828367

**Published:** 2026-06-23

**Authors:** Ayodeji Mathias Adegoke, Grace Ochanya Igomu, Saviour God’swealth Usin, Mamello Patience Sekhoacha

**Affiliations:** 1 Department of Pharmacology, University of the Free State, Bloemfontein, South Africa; 2 Cancer Research and Molecular Biology Laboratories, Department of Biochemistry, College of Medicine, University of Ibadan, Ibadan, Nigeria; 3 Department of Medical Biochemistry, Faculty of Basic Medical Sciences, University of Cross River State, Okuku, Yala, Nigeria

**Keywords:** antioxidants, breast cancer, breast cancer prevalence, dietary antioxidants, oxidative stress, reactive oxygen species, breast cancer diagnosis, breast cancer epidemiology

## Abstract

Breast cancer is a primary cause of cancer-related deaths among women globally. Timely diagnosis of breast cancer with effective treatment helps reduce mortality. Over the years, extensive research has linked oxidative stress, which results from the imbalance in Reactive Oxygen Species (ROS) and antioxidant homeostasis, to the formation of cancer cells. Oxidative stress can lead to carcinogenesis through its ability to alter genetic material (DNA), affect cellular signalling pathways and cell cycle regulation, promote angiogenesis and metastasis. Antioxidants are defence systems used to counteract ROS, thus ensuring ROS homeostasis in a cell. Examples of endogenous antioxidants include glutathione, catalase, and superoxide dismutase. Antioxidants can be obtained exogenously from plant sources or the diet. Examples of dietary antioxidants’ sources include curcumin, brassinosteroids, gallocatechins, resveratrol, etc. Chemotherapy has some undesirable side effects which can negatively affect the quality of life of breast cancer patients, natural plant-based products rich in antioxidants have been shown to mitigate these side effects and therefore improve patients’ quality of life. Various plants have been studied, and compounds having antioxidant properties have been identified that can help reduce the side effects of cancer treatment. The intake of dietary antioxidants has become important in cancer prevention and management. From various studies on ROS and antioxidants in breast cancer aetiology and treatment, there seems to be conflicting information suggesting contradictory roles of both ROS and antioxidants. Although ROS are seen to be involved in breast cancer formation, they are still used in treating cancer by inducing apoptosis. Some newer investigations have indicated certain antioxidants in promoting carcinoma. The concept of persistent oxidative stress in cancer cells, which supports tumour microenvironment (TME), is another phenomenon requiring more research. This review focuses on breast cancer, highlighting how oxidative stress contributes to its progression and evaluating the therapeutic potential of dietary antioxidants.

## Graphical abstract

**Figure:**
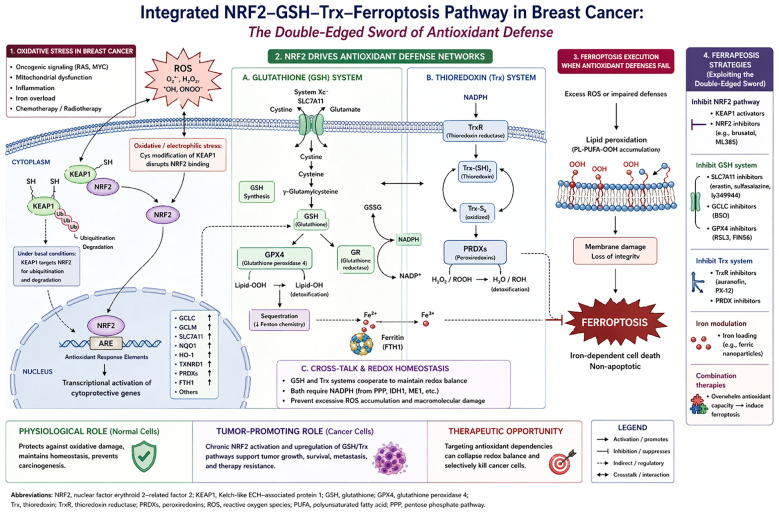

## Highlights

- ROS imbalance promotes DNA damage and drives breast cancer initiation and progression.
- Antioxidants regulate ROS and help maintain cellular homeostasis.
- ROS induce apoptosis and serve as therapeutic agents against cancer.
- Plant-derived antioxidants may reduce chemotherapy side effects.
- Certain antioxidants paradoxically promote tumour progression.

## Simple summary

Breast cancer is a major cause of cancer-related deaths among women globally. An imbalance in reactive oxygen species (ROS) and antioxidants results in oxidative stress, which plays a central role in breast cancer initiation and progression by damaging DNA, disrupting signalling pathways, and supporting angiogenesis. Antioxidants, either naturally produced in the body or obtained from the diet, help maintain ROS balance and may protect against cancer development. Plant-derived antioxidants also reduce chemotherapy side effects, improving patient well-being. However, both ROS and antioxidants have dual roles: ROS can induce cancer cell death, and some antioxidants may paradoxically promote tumour growth by reducing high levels of naturally occurring ROS in cancer cells, which would otherwise damage and kill the cells.

## Introduction

**Figure 1:**
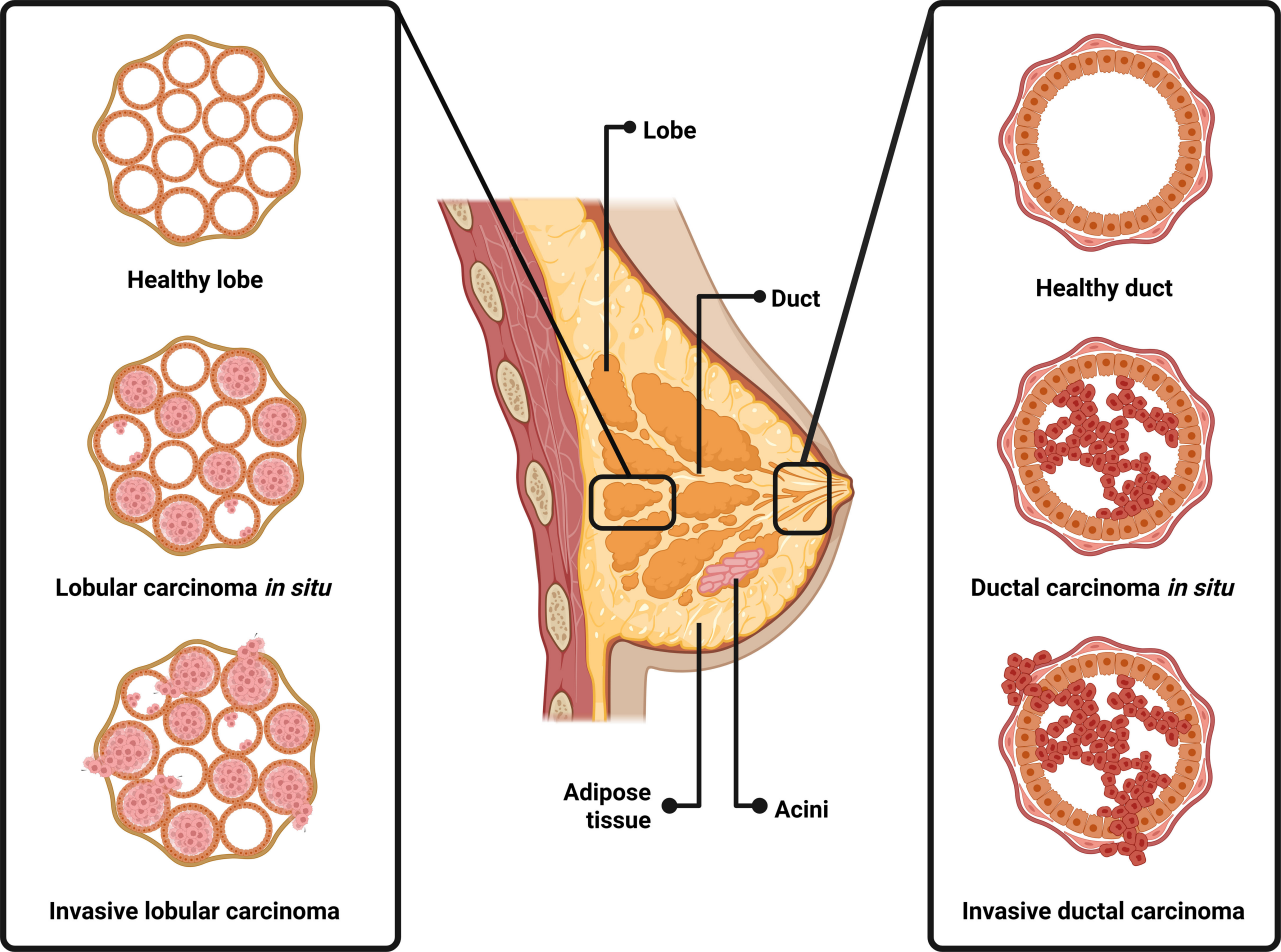
Morphological progression of breast tissue from healthy to cancerous states.

Among women worldwide, breast cancer accounts for approximately 25% of new cancer diagnoses (1) and is the leading cause of cancer deaths (2). Breast tumours are complex structures that consist of old stromal and neoplastic cells (3). Malignant breast tumour, which arises from the inner lining of milk ducts, is more common and is known as ductal carcinoma, while the less frequent type, known as lobular carcinoma, originates from the lobules that supply milk to the ducts (**Figure 1**) (4). Breast cancer is a heterogeneous disease resulting from both genetic and environmental risk factors (5). Breast cancer is a result of a genetic mutation, as discovered in the mutation of germline Breast Cancer gene 1 (*BRCA1*), which leads to basal breast cancer (6). Examples of risk factors of breast cancer include an increase in age, family history, type of nutritional diet, weight, alcohol intake and levels of exposure to oral contraceptives and hormone replacement therapies (7). Strategies employed in treating breast cancer are surgery, chemotherapy, radiation therapy and immunotherapy (8). Even the best of cancer treatments has been shown to have negative side effects like hair loss, fatigue, and limited hand movements. These changes, which affect the body image most at times, can lead to stress and anxiety in patients. Apart from affecting the organ of the patient, psychological issues from post-mastectomy can arise, like feelings of depression, humiliation and suicidal tendencies (9). Breast cancer progression has been linked to oxidative stress, a condition resulting from an imbalance between reactive oxygen species (ROS) production and the body’s ability to detoxify these reactive molecules. ROS are highly unstable molecules that can damage deoxyribonucleic acid (DNA), proteins and lipids. This damage can lead to cell death, inflammation and cancer. Dietary antioxidants are substances that can help neutralise ROS and protect cells from oxidative damage. There is a belief that diet might help in biological systems through action on different biological mechanisms like inflammation, immunity, angiogenesis, growth factors, and the regulation of the cell cycle (10, 11).

## Literature search strategy and study selection criteria

In 2025, a comprehensive literature search was conducted across multiple electronic databases, including Google Scholar, PubMed, SpringerLink, Elsevier ScienceDirect, and Web of Science, to identify relevant studies published between 2015 and 2025. In addition, earlier studies dating back to 2000 were also included where they were considered highly relevant to the scope of the review. Only articles published in the English language were considered for inclusion. The search strategy was based on a combination of keywords and subject headings, including: *“breast cancer,” “breast cancer and oxidative stress,” “breast cancer prevalence,” “reactive oxygen species in breast cancer,” “cancer statistics,” “medicinal plants in breast cancer treatment,” “dietary antioxidants and breast cancer,”* and *“antioxidants in breast cancer treatment.”* After the initial search, duplicate records were removed and the remaining studies were subjected to a two-stage screening process. Titles and abstracts were first screened for relevance, followed by full-text evaluation of eligible articles. Studies that did not meet the inclusion criteria were excluded during this process. The inclusion criteria comprised original research articles and review papers that were directly relevant to breast cancer, oxidative stress, antioxidant mechanisms, epidemiology, or related therapeutic approaches. The exclusion criteria included studies with insufficient or irrelevant information, as well as articles for which full-text versions were not accessible.

## Epidemiology of breast cancer: statistics and prevalence

Breast cancer, as of 2020, was ranked number one in the number of new cases with 11.8% of incident rates, and number 4 in the cause of cancer-related deaths, with Asia having an incidence rate of 45.4%, 23.5% in Europe, 12.5% in North America, 9.3% in Latin America and the Caribbean, and 8.3% in Africa (12, 13). Breast cancer incidence shows significant geographical variation (**Table 1**), reflecting differences in risk factors, healthcare access, screening practices, and socioeconomic development. Globally, there were approximately 2.26 million new breast cancer cases reported, representing a cumulative risk of 5.2% up to age 74. Mortality reached 684,996 deaths, with a cumulative risk of 1.49%, underscoring breast cancer as a critical public health challenge (14). The statistics emphasise both the growing prevalence of the disease and disparities between high- and low-resource settings. In Africa, the burden of breast cancer is rising. For instance, the Northern part of Africa recorded about 57,128 new cases with a cumulative risk of 5.12%, while Western Africa reported 49,339 new cases at a risk of 4.49% (14). In regions with lower breast cancer incidence, death-to-case ratios are high, indicating challenges such as late detection and restricted access to therapies. In the Eastern part of Africa, 45,709 new cases were reported (**Table 1**), but 24,047 deaths, translating to more than half of diagnosed patients succumbing to the disease, thus reflecting structural barriers such as poor access to mammographic screening, delayed presentation, and inadequate treatment infrastructure (2). The Americas display a contrasting picture. The Northern part of America documented the highest incidence within the region, with 281,591 new cases and a cumulative risk of 9.71%. However, mortality is relatively lower, with 48,407 deaths and a cumulative mortality risk of 1.36%, demonstrating the impact of early detection and advanced therapeutic options. More so, in the Southern part of America, 156,472 new cases have been reported with higher mortality (41,661 deaths), illustrating disparities within the same continent. In Asia, the patterns are diverse. Eastern Asia, with its large population, had a striking 551,638 new cases, though the cumulative risk was modest at 4.6%. Mortality was 148,471 deaths, reflecting ongoing challenges in health system capacity. South-Central Asia also bore a heavy burden with 254,881 cases and 121,670 deaths, indicating significant survival challenges. Meanwhile, Western Asia had a lower incidence (60,715 cases) but still considerable mortality (20,493 deaths). These regional differences underline the interplay between population size, lifestyle transitions, and healthcare infrastructure. Europe shows the highest breast cancer risks. Northern Europe reported 83,177 new cases with a cumulative risk of 9.35%, among the highest worldwide, yet mortality was relatively controlled at 17,964 deaths. Western Europe also had a high incidence (169,016 cases) and comparatively fewer deaths (43,706). These figures reflect widespread screening, early detection, and access to advanced treatments, which improve survival despite high case numbers. Similarly, Australia and New Zealand showed 23,777 new cases and 3,792 deaths, with incidence driven by lifestyle factors but mitigated by robust healthcare systems (14, 15). Incidence and mortality data were obtained from the GLOBOCAN database (International Agency for Research on Cancer; IARC), which provides modelled national and regional cancer estimates based on available registry data.

**Table 1:** Breast cancer incidence and mortality by world region (GLOBOCAN estimates) (12, 13).

Region	New cases	Deaths	MIR (deaths per 100 cases)
Eastern Africa	45709	24047	52.6088954
top Africa	17896	9500	53.08448815
Northern Africa	57128	21524	37.67679597
Southern Africa	16526	5090	30.79995159
Western Africa	49339	25626	51.93862867
Caribbean	14712	5874	39.92659054
Central America	38916	16429	42.21656902
South America	156472	41661	26.6252109
Northern America	281591	48407	17.19053521
Eastern Asia	551638	148471	26.91457079
South-Eastern Asia	158893	58677	36.92862492
South-Central Asia	254881	121670	47.73600229
Western Asia	60715	20493	33.75277938
Central & Eastern Europe	195706	102311	52.27790666
Western Europe	169016	43706	25.85909026
Southern Europe	118057	34536	29.2536656
Northern Europe	83177	17964	21.59731657
Australia & New Zealand	23777	3792	15.94818522
Melanesia	2215	1121	50.60948081
Polynesia	120	82	68.33333333
Micronesia	103	38	36.89320388

Mortality-to-incidence ratio (MIR) was calculated as (deaths ÷ incident cases) × 100. MIR is an ecological indicator and does not represent patient-level survival outcomes.

Furthermore, when comparing by Human Development Index (HDI), the disparities are even clearer. Very high HDI regions reported the greatest incidence (1,017,459 cases) with a cumulative risk of 8.17% (**Table 2**), yet lower mortality relative to incidence (231,096 deaths). Conversely, low HDI regions had fewer cases (109,572) but extremely high mortality (58,586 deaths) (**Table 2**), with a mortality-to-incidence ratio more than double that of high HDI regions (16). Breast cancer global estimates reveal a striking imbalance in the distribution of breast cancer burden according to human development. For instance, in countries with a very high HDI, 1 in 12 women will be diagnosed, and 1 in 71 women will die; in low-HDI countries, 1 in 27 will be diagnosed, and 1 in 48 will die (12, 13). However, in the year 2022, the IARC projected that by 2040, the burden of breast cancer is expected to increase, with projections to more than 3 million new cases per year and more than 1 million deaths per year (40% and 50% increase, respectively). In 2020, there were about 2.3 million new cases and about 685,000 deaths, with large geographical variation (17, 18).

**Table 2:** Breast cancer burden across human development index (HDI) levels.

Region	New cases	Deaths	MIR (deaths per 100 cases)
Low HDI	109572	58586	53.46803928
Medium HDI	307658	147447	47.92561871
High HDI	828438	247486	29.87381071
Very High HDI	1017459	231096	22.71305281

Mortality-to-incidence ratio (MIR) was calculated as (deaths ÷ incident cases) × 100. MIR is an ecological indicator and does not represent patient-level survival outcomes.

## Risk factors and contributing factors to breast cancer

Breast cancer is a multifactorial or heterogeneous disease that develops from the dual influence of genetic and environmental factors.

### Family history of breast cancer

Among risk factors for breast cancer, family history seems to be the strongest risk, with almost 20% of all breast cancers having a family origin and are dependent in an etiological fashion on a specific predisposing gene of that disease (5). Breast cancer risk is often inherited in an autosomal dominant pattern with incomplete penetrance, allowing carriers to transmit the gene without developing the disease. Mutations in Breast Cancer gene 1 (*BRCA1*) and Breast Cancer gene 2 (*BRCA2*), located on chromosomes 17 and 13, respectively, account for many high-risk families. Women with a first-degree relative diagnosed before age 50 have at least a twofold increased risk of developing breast cancer (7).

### Obesity

Obesity, reflected by a high body mass index (BMI), is associated with an increased risk of breast cancer in postmenopausal women, whereas this association is not observed in premenopausal women (19).

### Oral contraceptives

Use of oral contraceptives is linked to a slight rise in breast cancer risk, which diminishes over time after cessation, with negligible risk ten years post-use. Initiation at an older age, however, may increase susceptibility (5, 7).

### Hormone regulation

Hormone regulation plays a role in breast cancer development. Early pregnancy can reduce breast cancer incidence, while delayed menopause is linked with breast cancer development (7).

### Nutritional diet

Diet may act on different biological mechanisms, such as inflammation, immunity, cell regulation factors, angiogenesis, and growth factors, in the intervention of carcinogenesis (11). Diets such as the Mediterranean diet, rich in plant-based foods and limited in red meat and animal fats, are linked to decreased cancer risk (20, 21). Reduced calorie intake and even fasting cycles can reorder cellular metabolism and protect it from damage due to oxidative stress. The use of dietary restriction regimens can be beneficial in preventing cancer and enhancing therapeutic responses (22, 23).

## Molecular mechanisms involved in breast cancer development

**Figure 2:**
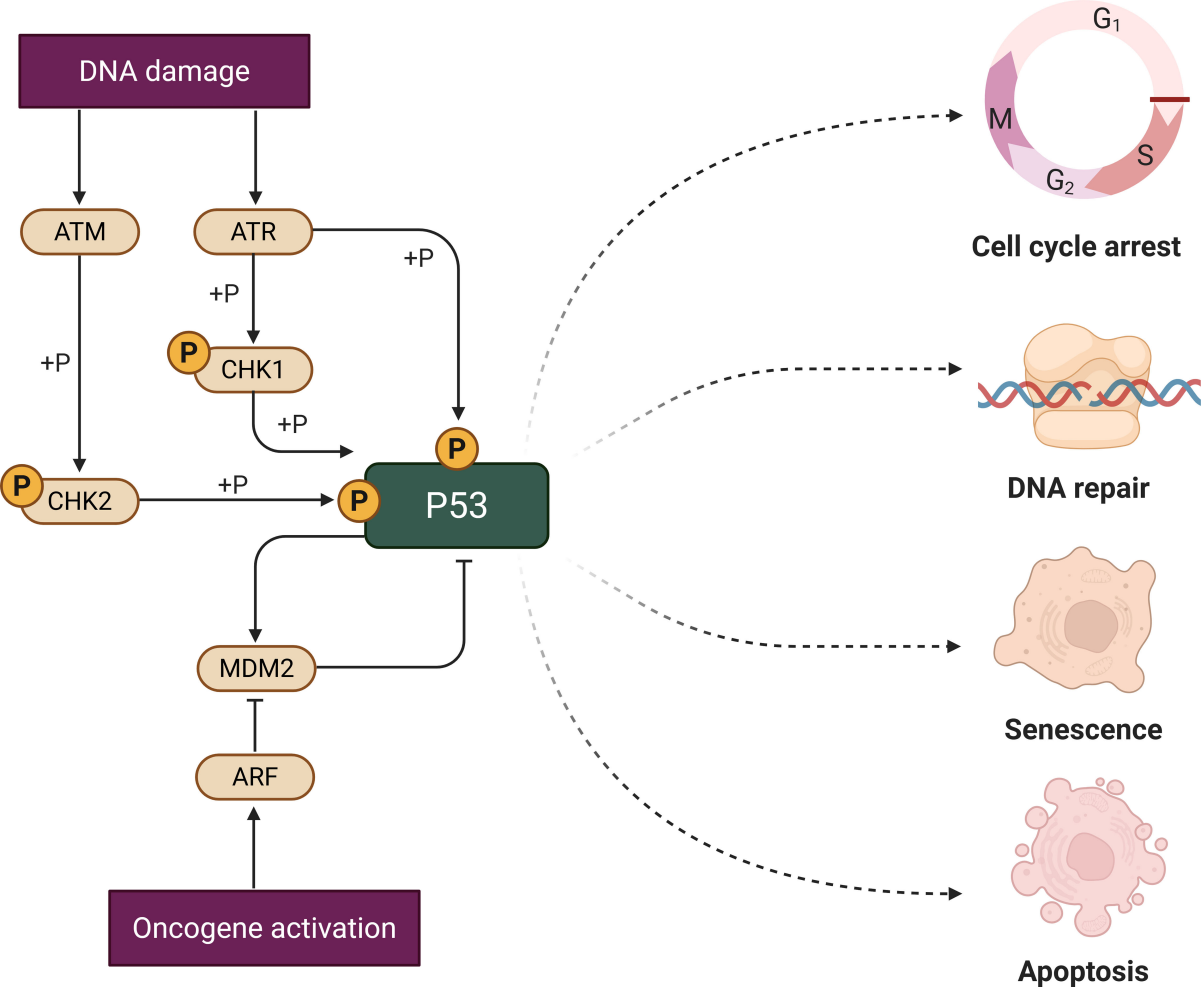
p53 regulation and signalling in breast cancer.

Carcinogenesis is a complex, multi-step process driven by a variety of molecular and cellular mechanisms (24). The formation of the tumour is believed to result from a series of progressive changes that involve the activation of oncogenes and the inactivation of tumour suppressor genes. Understanding the important role of cellular processes in triggering, regulating, and affecting cell death and cell proliferation can help determine the natural history and chemotherapeutic response of tumours (25–27). Due to the opposite effects of oncogenes and tumour suppressor genes on cell cycle progression, it was implied that the abnormalities in the growth of tumour cells are a result of the combination of insufficient tumour suppressors (cell cycle breaks) and insufficient oncogenes (cycle accelerators) (28). A gain-of-function mutation in a proto-oncogene converts it into an oncogene, producing tumour-promoting proteins, whereas a mutation in a tumour suppressor gene leads to loss of function, impairing its ability to regulate cell growth. Many oncogenes are categorised among growth factors that are abnormally activated, intracellular signalling molecules, growth factor receptors and nuclear transcription factors. Tumour suppressor genes with observed effects on the cell cycle regulation are retinoblastoma protein (pRb) and tumour protein p53 (p53) (29–31). p53 plays a central role in maintaining cellular integrity by regulating cell cycle progression, DNA repair, gene transcription, and genomic stability (32–34). Loss or mutation of p53 disrupts these pathways, promoting cellular immortalisation, checkpoint defects, and genomic instability, which allows replication of damaged DNA and contributes to tumourigenesis (35, 36). Common mutations in p53, including missense, nonsense, and frameshift mutations, can increase the protein’s half-life, leading to the accumulation of dysfunctional mutant p53 that impairs normal tumour-suppressive functions (37, 38). Key downstream effectors of p53 include Mouse Double Minute 2 (MDM2) and cyclin-dependent kinase inhibitor 1 (p21), which mediate critical regulatory pathways in breast cancer. MDM2 functions as a negative regulator of p53, targeting it for ubiquitin-mediated degradation and thereby modulating p53-dependent apoptotic and growth-arrest pathways (39, 40). In contrast, p21 enforces cell cycle checkpoints by inhibiting cyclin-dependent kinase (Cdk) complexes, blocking the G1-to-S phase transition and executing p53-dependent growth arrest (**Figure 2**) (41, 42). The functional status of p53, in combination with other molecular markers, such as oestrogen and progesterone receptors, B-cell lymphoma 2 (Bcl-2), BCL2-associated X (Bax), and human epidermal growth factor receptor 2 (HER-2), provides insight into tumour proliferative capacity and replicative potential, making it a critical mechanistic hub in breast cancer biology (43–45).

In mammary epithelial cells, Bcl-2 is present in non-pregnant women and during early pregnancy, yet it is undetectable during lactation (46). The family members of Bcl-2 can both be pro-survival; B-cell lymphoma-extra-large (Bcl-XL) and Bcl-2, and pro-apoptosis; Bcl-2 agonist of cell death (Bad), Bcl-2-associated X (Bax), and BH3 interacting-domain death agonist (Bid) (47, 48). Due to the importance of maintaining equilibrium in cell proliferation and apoptosis for the normal functioning and development of the breast, Bcl-2, which also expresses oestrogen receptor, is needed in adult mammary glands (49, 50). Although the Bcl-2 protein promotes cell survival, it does not stimulate cell replication or proliferation. By prolonging the cell cycle, breast cancer cells expressing Bcl-2 exhibit an increased doubling time, a reduced S-phase fraction, and accumulation in the G1/G0 phase, processes that are regulated by distinct genetic pathways (51, 52). The 26 kDa Bcl-2 oncoprotein associates with intracellular membranes via a hydrophobic region near its C-terminus. This localisation targets Bcl-2 to membranes of the endoplasmic reticulum, mitochondria, and perinuclear region, while the majority of the protein resides in the cytoplasm (53).

**Figure 3:**
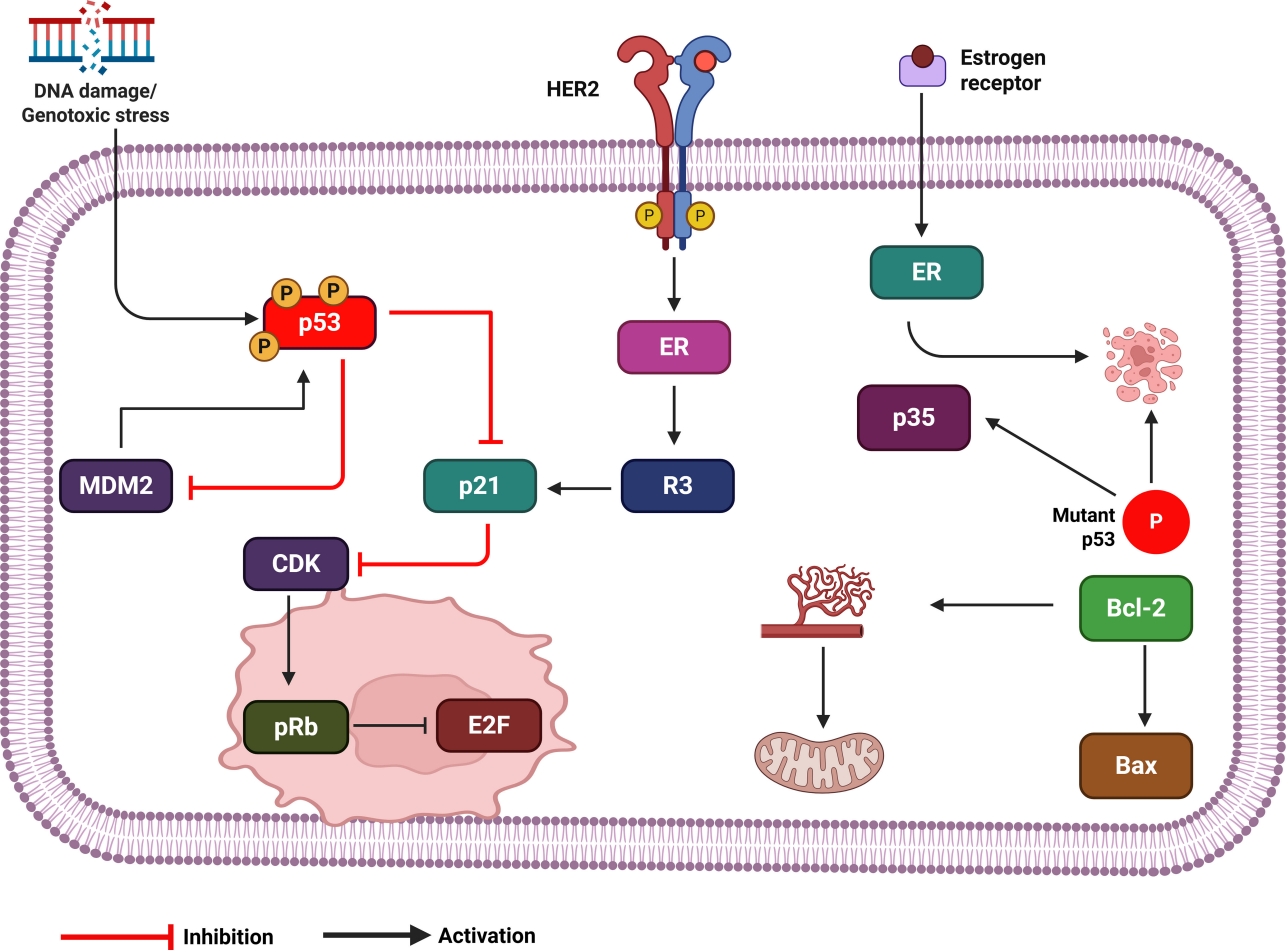
Molecular mechanisms involved in breast cancer development.

The expression of Bcl-2 has been shown to correlate with oestrogen receptor (ER) and progesterone receptor (PR) status in breast cancer. Exposure to 17β-estradiol upregulates the anti-apoptotic protein Bcl-2 while downregulating Bcl-XL, highlighting hormone-mediated modulation of apoptosis (54). Members of the Bcl-2 protein family are regulated through pathways influenced by estradiol, such that tumour cell expression of oestrogen receptors directly controls Bcl-2 levels (55, 56) Bcl-2-associated athanogene-1 (Bag-1), a Bcl-2-associated athanogene, functions as an anti-apoptotic protein by interacting with Bcl-2. It also binds hormone receptors, including ER, to inhibit hormone-induced apoptosis. This interaction is critical in breast cancer oncogenesis and progression, particularly as Bag-1 activity is enhanced by mutant p53, further promoting cell survival and resistance to apoptosis (**Figure 3**) (57, 58).

Human epidermal growth factor receptor 2/neuroglialoblastoma-derived oncogene (HER/2neu), a proto-oncogene located on chromosome 17q11.2-q12, is a well-recognised tumour-associated marker. It encodes a 185 kDa transmembrane glycoprotein with intrinsic tyrosine kinase activity and is a member of the epidermal growth factor receptor (EGFR) family (59) HER2/neu plays critical roles in cellular development, proliferation, and differentiation in tissues where it is expressed. Although no direct ligand has been identified for HER2/neu, it can be activated through overexpression or transactivation by ligands of other EGF family receptors (37, 60, 61) In addition, the phosphatase protein tyrosine phosphatase 1B (PTP1B) regulates tyrosine kinase signalling by dephosphorylating key kinases involved in breast tumourigenesis, including HER1/EGFR, proto-oncogene tyrosine-protein kinase (Src), janus kinase (JAK), and signal transducer and activator of transcription (STAT). Overexpression or mutation of PTP1B has been observed in breast cancer cells, suggesting its role in promoting oncogenic signalling and tumour development (62).

Cell-cycle, angiogenesis, metastasis and evasion are signalling pathways associated with the formation of cancerous cells (63). Numerous cell cycle regulating systems are usually impaired in cancerous cells, which is frequently observed in the phenotype of the malignant tumour cells (64). The retinoblastoma gene (Rb) encodes a tumour suppressor protein critical for controlling the G1 checkpoint of the cell cycle, which regulates the transition from G1 to S phase. The wild-type pRb protein binds to E2F transcription factors, preventing them from promoting DNA replication and cell cycle progression. However, mutant or inactivated pRb (mt-pRb) cannot bind E2F, allowing uncontrolled activation of E2F targets and enabling cells to progress through the cell cycle independently of normal regulatory cues. This loss of Rb function is observed in many cancers and permits tumour cell proliferation without external growth signals, representing one of the hallmarks of cancer described by Hanahan and Weinberg (**Figure 3**) (65, 66).

## Iron-driven redox vulnerabilities and ferroptosis pathways in breast cancer

Dysregulated iron metabolism is increasingly recognised as a hallmark of breast cancer, with particularly strong implications for Triple-negative breast cancer. Cancer cells reprogram iron handling to sustain rapid proliferation, leading to expansion of the intracellular labile iron pool (LIP), composed largely of redox-active ferrous iron (Fe^2+^) (67, 68). This pool fuels essential processes such as DNA synthesis and mitochondrial respiration, but simultaneously predisposes cells to oxidative stress through iron-catalysed ROS generation (69, 70). In breast tumours, elevated transferrin receptor (TFRC) expression and reduced ferroportin (SLC40A1) levels promote iron accumulation and correlate with poor prognosis (71–73) TNBC, in particular, displays heightened iron dependency, reflecting its aggressive phenotype and metabolic plasticity (74–76). At the biochemical level, iron-mediated oxidative stress is largely driven by the Fenton reaction, which converts hydrogen peroxide into highly reactive hydroxyl radicals capable of inducing widespread biomolecular damage.

### Fe^2+^ + H_2_O_2_ → Fe^3+^ + ·OH + OH^-^

**Figure 4:**
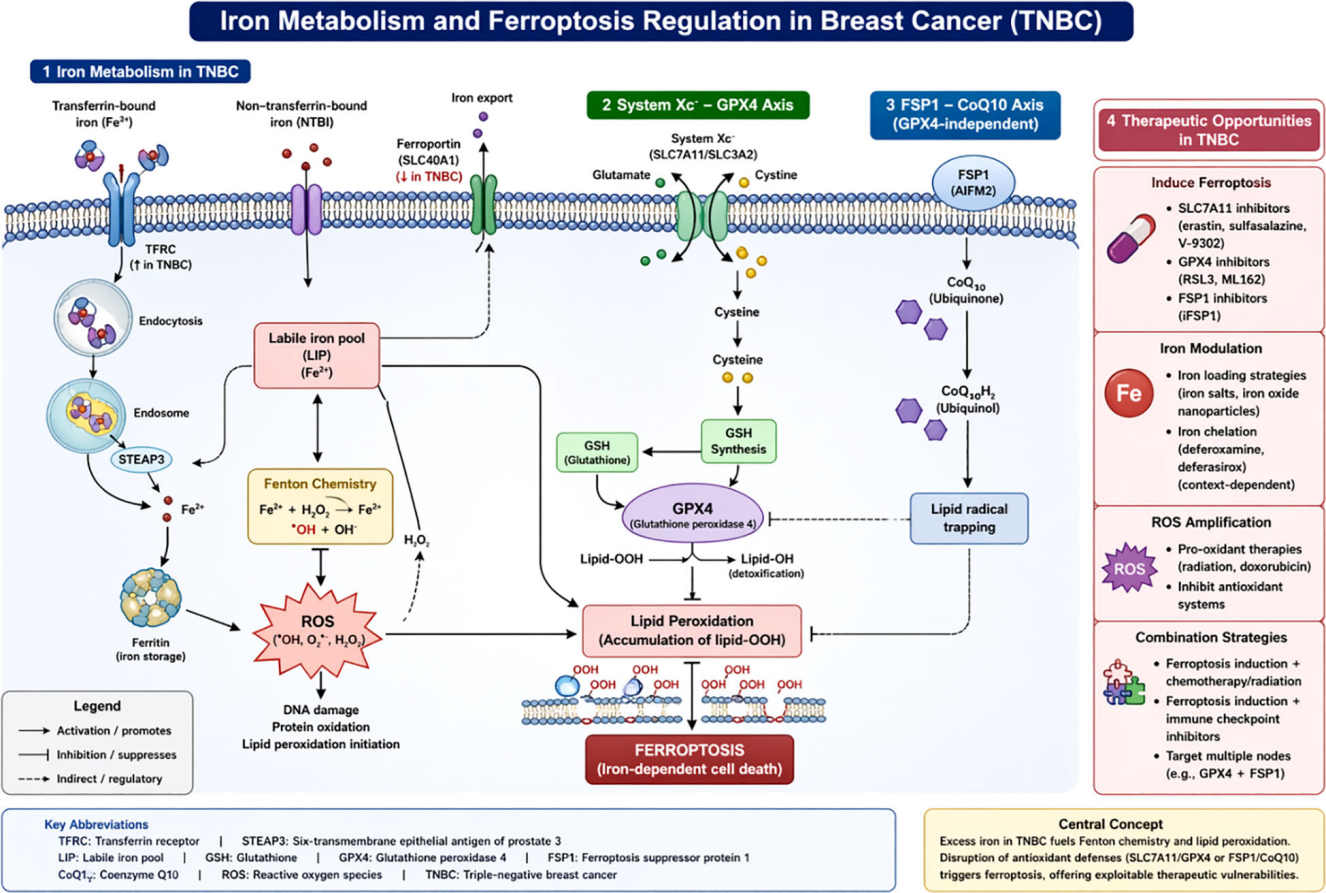
Iron metabolism and regulation of ferroptosis in breast cancer.

Hydroxyl radicals generated via this reaction initiate lipid peroxidation, particularly in membranes enriched with polyunsaturated fatty acids (PUFAs), thereby compromising membrane integrity and cellular viability (77, 78). In TNBC, where basal ROS levels are already elevated due to oncogenic signalling and mitochondrial dysfunction, the presence of excess catalytic iron creates a precarious redox balance (79). This condition places tumour cells near a threshold beyond which oxidative damage becomes lethal, offering a window for therapeutic exploitation. A critical outcome of iron-driven lipid peroxidation is the activation of ferroptosis, a regulated, non-apoptotic form of cell death characterised by the accumulation of lipid hydroperoxides (80, 81) Ferroptosis is morphologically and biochemically distinct from apoptosis, necrosis, and autophagy, and is uniquely dependent on intracellular iron availability and oxidative lipid damage (82–84). The execution of ferroptosis is governed by a tightly regulated network of antioxidant defence systems, among which the GPX4-GSH axis plays a central role. Glutathione peroxidase 4 (GPX4) detoxifies lipid hydroperoxides into lipid alcohols (85, 86), thereby preventing the propagation of lipid peroxidation chains. This process requires glutathione (GSH), whose synthesis depends on cysteine availability, primarily supplied through the Solute Carrier Family 7 Member 11 (SLC7A11) (system Xc^-^) cystine/glutamate antiporter (**Figure 4**) (87).

In parallel, an alternative ferroptosis defence pathway is mediated by ferroptosis suppressor protein 1 (FSP1), which operates independently of GPX4 by reducing coenzyme Q10 (CoQ10) to its active antioxidant form, ubiquinol. This pathway provides a lipid radical-trapping mechanism at the plasma membrane (88, 89), thereby limiting lipid peroxidation. Additional modulators, including Acyl-CoA Synthetase Long-Chain Family Member 4 (ACSL4), which promotes PUFA incorporation into membrane phospholipids, and lipoxygenases (LOXs), further influence ferroptosis sensitivity by regulating lipid composition and oxidation dynamics (90, 91). Notably, TNBC exhibits molecular and metabolic characteristics that enhance its susceptibility to ferroptosis. These include enrichment of mesenchymal-like states, increased PUFA-containing phospholipids, and elevated basal ROS levels (92). However, TNBC cells often upregulate ferroptosis defence mechanisms such as GPX4, SLC7A11, and FSP1 as adaptive responses, creating a state of “non-oncogene addiction” to antioxidant pathways (93–96). This dependency represents a critical vulnerability that can be therapeutically targeted. From a therapeutic perspective, targeting iron-dependent redox pathways offers multiple intervention points. Ferroptosis can be pharmacologically induced through inhibition of system Xc^-^ (e.g., erastin and its derivatives), direct inhibition of GPX4 (e.g., RSL3), or disruption of the FSP1-CoQ10 axis (97, 98). These approaches lead to unchecked lipid peroxidation and rapid cell death in susceptible cancer cells. Importantly, iron modulation strategies can further potentiate ferroptosis. Increasing intracellular iron levels enhances Fenton chemistry and ROS generation, whereas targeted iron delivery systems (e.g., iron oxide nanoparticles) can selectively amplify oxidative stress within tumours (99, 100).

Conversely, iron chelation strategies, using agents such as deferiprone, deferoxamine, and deferasirox, may be beneficial in early-stage or iron-addicted tumours by limiting proliferation and reducing oxidative damage to surrounding tissues (101–104). Thus, the therapeutic use of iron modulation must be context-dependent, guided by tumour stage, subtype, and metabolic profile. In addition, combination therapies represent a particularly promising avenue for maximising the efficacy of ferroptosis-based interventions. For instance, combining GPX4 inhibitors with chemotherapeutic agents or radiotherapy can enhance ROS accumulation and overwhelm cellular antioxidant defences (**Figure 4**) (105–107). Similarly, inhibition of SLC7A11 has been shown to sensitise TNBC cells to standard-of-care treatments by impairing glutathione synthesis and redox buffering capacity (108). Emerging evidence also suggests that ferroptosis induction can stimulate anti-tumour immunity by releasing damage-associated molecular patterns (DAMPs), thereby enhancing the efficacy of immune checkpoint inhibitors (109–112). Despite these advances, several challenges remain in translating ferroptosis-targeted therapies into clinical practice. Tumour heterogeneity, adaptive resistance mechanisms, and potential toxicity to normal tissues are key concerns. Identifying robust biomarkers, such as GPX4 expression, lipid peroxidation signatures, or iron metabolism markers, will be essential for patient stratification and therapeutic optimisation. Furthermore, the interplay between ferroptosis and other cell death pathways requires deeper investigation to design rational combination strategies.

## Breast cancer metastasis

Metastasis involves the spread of cancer cells from the site of tumorigenesis to nearby tissues or organs (113). It is a distinct characteristic of cancer disease and a significant obstacle in cancer treatment (114). It is the primary cause of mortality among different cancer types, as well as breast cancer (115). Approximately 90% of deaths from breast cancer are caused by breast cancer metastasis (116). Primary breast cancer cells normally spread through the blood vessels or lymph nodes to different organs like the liver, lungs and bones. The spread of breast cancer cells generally consists of the metastasis process, which is found in several solid tumours (117). They include;

- *Removal of breast cancer cells from extracellular matrix (ECM) and local invasion and migration initiation:* Disruption of the connection of the cell to ECM through cellular adhesion proteins such as integrins, resulting in the dissociation of cancer cells from adjacent cells and the basement membrane, describes how metastasis begins (118). Cells possessing this ability of enhanced invasion start to invade surrounding tissue with the support of proteolytic enzymes secreted to degrade the ECM and provide a route of invasion (119).
- *Intravasation or entry of cancerous cells into the circulation:* Cancer cells, after being fastened to the endothelial wall, invade and pass through the walls of lymph or blood vessels (120).
- *Circulation:* The blood or lymphatic circulation helps to spread tumour cells to other organs of the body. For the cells to be able to survive in an anchorage-independent manner, they must possess anoikis resistance (121).
- *Activities at the site of metastasis, which include arrest, adhesion, and extravasation:* Preceding the extravasation into the site of metastasis, tumour cells experience cell-cycle arrest and fasten themselves to capillary walls in target organs (120).
- *Metastatic tumour formation:* There will be proliferation and formation of small tumours by cancer cells with tumorigenic potential (122).

Since metastasis is a multi-step process, the metastatic cells will need some properties to overcome hindrances, and the ability to survive in detached conditions, to invade, and to form new tumours. Interrupting any of these steps will stop the process of cancer metastasis (123, 124). Oxidative stress in breast tumours can lead to metastasis through the activation of matrix metalloproteinases (MMPs) and the inhibition of antiproteases. Matrix metallopeptidase 2 (MMP-2) is a gelatinase believed to play a crucial role in breast cancer metastasis. Poor prognosis in breast cancer patients is associated with high levels of MMP-2 (125). ROS is observed in the activation of MMP-2, potentially by the reaction of oxygen radicals with thiol groups found in MMP-2 (126, 127). Approximately 90% of breast cancer-related deaths are linked to metastasis of the cancer cells (128, 129).

## Types and stages of breast cancer

**Figure 5:**
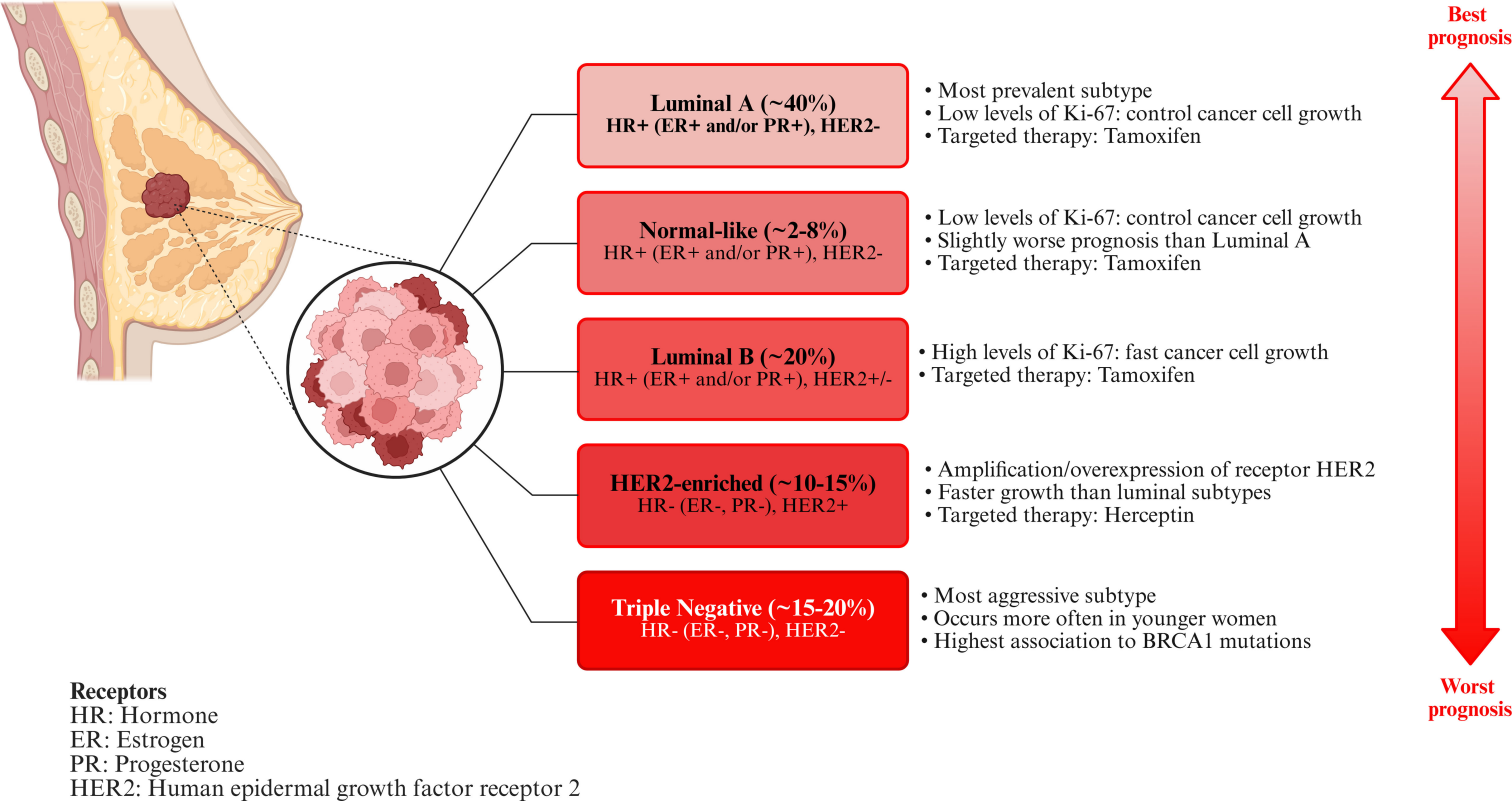
Breast cancer subtypes and their prognosis.

Breast cancer can be classified into non-invasive (carcinoma *in situ*) and invasive carcinoma. In non-invasive breast cancer, malignant cells remain confined to the ducts or lobules and do not invade the surrounding fatty or connective tissues, whereas invasive breast cancer is characterised by cancer cells breaching the ductal or lobular walls and infiltrating adjacent tissues (130) Molecular profiling has further divided breast cancer into four major subtypes: Luminal A, Luminal B, basal-like/triple negative, and HER2-enriched (131, 132) Luminal A tumours exhibit high expression of ER and PR, but low HER2 expression (133–135) Luminal B tumours show lower ER and PR levels, variable HER2 expression, and elevated expression of proliferation-associated genes (135, 136). The basal-like subtype, commonly referred to as triple negative, lacks ER, PR, and HER2 expression, accounts for approximately 15% of breast cancers, and is associated with poor prognosis (137–139) HER2-enriched tumours display high HER2 expression, increased proliferation, and are typically ER- and PR-negative (**Figure 5**) (135, 140, 141). More recently, a low-claudin subtype has been identified. Like basal-like tumours, it is triple negative (ER-, PR-, HER2-) but differs in having low Ki67 expression and elevated markers associated with epithelial-mesenchymal transition, indicating a distinct biological behaviour (142, 143).

**Figure 6:**
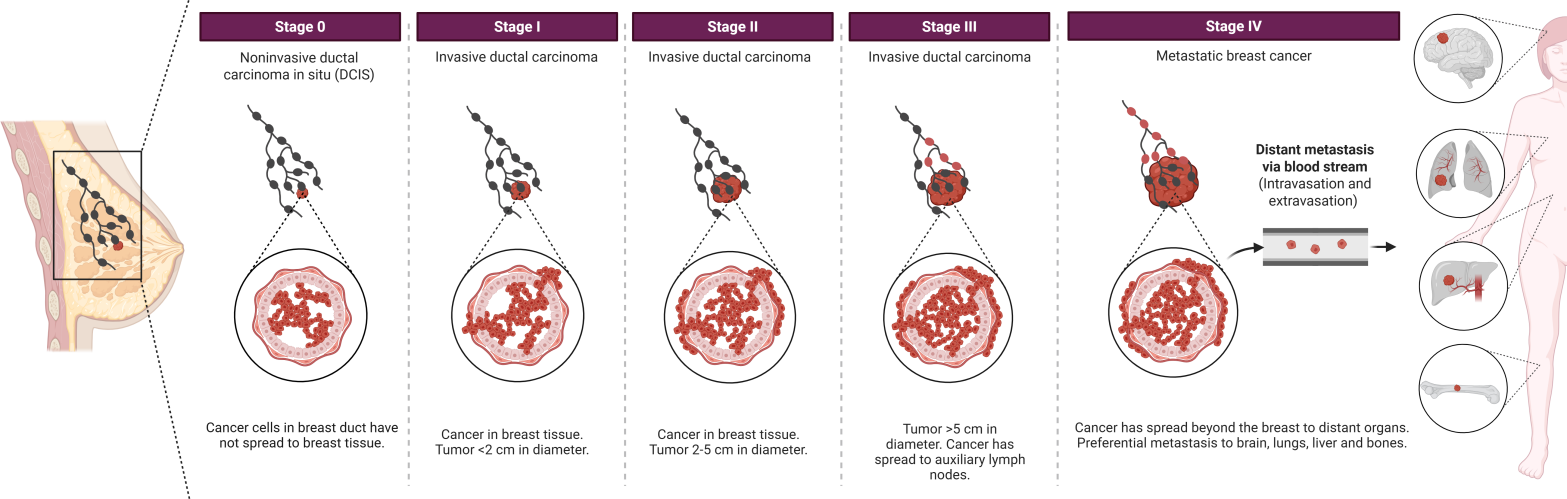
Stages of breast cancer.

With reference to the American Joint Committee on Cancer (AJCC), the stages of cancer can be classified into what is called the TNM system, where T is the breast tumour size, N is the extent of breast tumour spread to nearby lymph nodes, and M represents the extent of breast tumour metastasis to other parts or organs of the body (**Table 3**) (144–147). Stage 0 or carcinoma *in situ* is the earliest stage of cancer. Stage I indicates that the tumour is small (less than 2 cm in diameter in size) and is still within the patient’s breast. For stage II breast cancer, the tumour is within 2–5 cm in diameter and may be found in some axillary lymph nodes. For stage III, the breast tumour found may be of any size, but the axillary cancer will not be equal to that of stage II. In this stage, cancer has spread to the chest wall and/or to the breast skin, leading to dimpling, inflammation or breast skin change in colour (148). In stage 4 of breast cancer, the cancer has spread to farther organs like the brain, lungs, kidneys, and bone marrow (**Figure 6**; **Table 3**) (149).

**Table 3:** The TNM system stages of cancer from 0 to IV, detailing the type of breast cancers and corresponding five-year rate of survival (150).

Stage	Type	Five-year survival rate
0	Ductal carcinoma *in situ* or lobular carcinoma *in situ*.	92%
I	Invasive carcinoma 2 cm in size (including carcinoma *in situ* with micro invasion) without nodal involvement, but with movable axillary nodes and no distant metastasis.	87%
II	Invasive carcinoma <5 cm metastasis.	75%
III	Invasive carcinoma <5 cm in size with nodal involvement and fixed axillary nodes.	46%
IV	Any form of cancer with distant metastasis.	13%

## Breast cancer diagnostics

**Figure 7:**
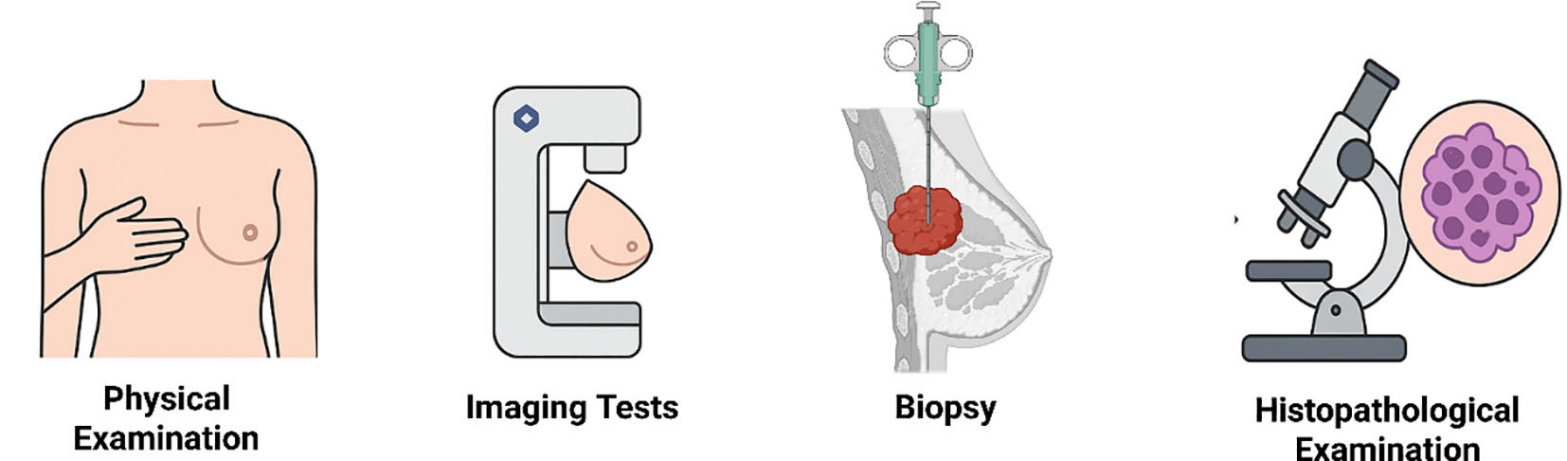
Diagnosis of breast cancer.

The diagnosis of breast cancer is usually made from screening or a symptom, such as pain or a palpable mass, that suggests a diagnostic examination (151). Breast cancer screening involves the observation of precancerous lesions and infiltrating tumours at early stages in females who do not present symptoms, with qualitative and economic methods. This screening process aims to lower mortality rates among breast cancer patients through early detection and prompt treatment. General recommendations of screening for females at normal risk of the disease include monthly breast examination, clinical breast examination, opportunistic screening, mammogram and ultrasound (**Figure 7**).

### Breast cancer examination

A woman needs to know the topography of her breasts in order to note changes in her breasts, if they should occur in the future. Breast cancer examination involves two basic steps, which are visual and tactile breast examinations. The visual examination is normally carried out with the aid of a mirror to notice any changes in the breast, like dimpling or puckering of the skin, a change in the direction of the nipple, breast contour changes like swelling, and “orange peel” appearance of the breast skin (150, 152). The tactile examination makes use of various palpation techniques, which include the vertical strip, wedge section and/or concentric circle detection methods to notice changes in texture or feel of the breast (150, 152, 153). The discovery of a lump or hard knot found in the breast or armpit, thickening or swelling of the breast, discharge of fluid other than breast milk without squeezing the nipple and any other unusual discovery should be reported to the physician. Although breast examination has been supported by many cancer organisations, it should not be used as a substitute for clinical breast examination and mammography (154, 155).

### Mammography vs ultrasound

Mammography remains the first-line and gold standard imaging modality for breast cancer screening, with proven evidence of mortality reduction at the population level. It makes use of low-energy X-rays (around 30kVp) to examine the breast (156, 157). The purpose of screening is to detect small (<1 cm) tumours through characteristic masses and/or microcalcification. From age forty, the American Cancer Society and the American College of Radiology recommend yearly mammograms. Early detection of breast cancer with smaller lesions via mammography can help to reduce mortality (158, 159). Mammography is also used to monitor the breast after breast-conserving surgery and external beam radiation therapy (160, 161). Ultrasound is not a replacement for mammography but serves as an important adjunct tool, particularly in women with dense breast tissue or in the evaluation of palpable abnormalities. However, ultrasound alone is not recommended for routine screening due to lower sensitivity for microcalcifications and higher false-positive rates, which may lead to unnecessary biopsies and follow-up imaging. Breast ultrasonography detects tumours by bouncing acoustic waves off breast tissues (162). To capture the structure of the breast, an ultrasound transducer is used to measure the acoustic waves that are reflected from the breast. Compared to mammography, it is less efficient (163, 164).

### Magnetic resonance image

Breast magnetic resonance imaging (MRI) has high diagnostic sensitivity (often exceeding 90%) and plays a critical role in selected clinical settings. It is recommended for high-risk screening populations (e.g., *BRCA* mutation carriers), preoperative staging, evaluation of treatment response, and problem-solving in inconclusive cases. Although MRI offers superior lesion detection, its use is limited by moderate specificity, higher cost, and increased likelihood of false-positive findings, which necessitate careful clinical correlation. MRI creates its image by applying a strong magnetic field with radiofrequency (RF) signals at different cross sections. For an increase in the resolution of an MRI image, a contrast agent can be applied (165, 166). It is more sensitive but less specific in detecting small tumours in people with high-risk cancer (167, 168).

### Microwave imaging techniques and emerging modalities

Microwave imaging and other emerging breast imaging techniques are currently regarded as investigational and non-standard modalities within clinical breast imaging practice. Passive MI uses radiometry to measure the differences in temperature between normal and cancerous tissues, while active MI is based on measuring dielectric properties (DPs) contrast between normal and cancerous tissues in the high-MHz and low-GHz regime (169). Active MI is a mammography technique for detecting breast cancer. Successful clinical trials of MI show that it may have the ability to be a low-risk alternative to complement the use of the mammography technique in diagnosing breast cancer (170). Although these techniques are based on promising biophysical principles, particularly the exploitation of differences in dielectric properties between malignant and normal breast tissues, their clinical translation remains limited. In experimental and early-phase clinical studies, MI has demonstrated potential in detecting tumour-associated variations in tissue conductivity and permittivity (169, 171–176), which may allow for differentiation between benign and malignant lesions under controlled conditions. However, despite these encouraging preliminary findings, the current body of evidence is constrained by small sample sizes, heterogeneous study designs, and a lack of standardised imaging protocols, which collectively limit reproducibility and generalisability. Furthermore, diagnostic performance metrics reported in early studies vary widely, and there is insufficient validation against established reference standards such as histopathology and multimodality imaging (mammography, ultrasound, and MRI) (177–179). Importantly, major international guideline bodies, including the ACR Appropriateness Criteria^®^, NCCN Clinical Practice Guidelines in Oncology, and EUSOBI recommendations, do not currently endorse microwave imaging for routine breast cancer screening, diagnostic work-up, or clinical decision-making. In addition, key technical and translational challenges remain, including limited spatial resolution, susceptibility to motion artefacts, variability in breast composition, and lack of standardised reconstruction algorithms, all of which hinder clinical implementation. Nevertheless, the MI technique still presents challenges that need to be addressed before it can be implemented in clinical trials (180).

## Tumour redox state, hypoxia, and acidosis imaging

Tumour microenvironmental characteristics such as hypoxia, altered redox balance, and extracellular acidosis have emerged as central determinants of tumour aggressiveness, metastatic potential, and therapeutic resistance (181, 182). These biological processes are tightly linked to metabolic reprogramming, particularly the shift toward glycolytic metabolism and impaired perfusion in rapidly proliferating tumours. As a result, there has been increasing interest in imaging strategies that move beyond structural assessment to capture functional and molecular features of tumour physiology *in vivo*. Optical imaging approaches using fluorescence- and bioluminescence-based probes have provided important experimental insights into tumour redox biology (183, 184). These probes can be designed to respond to intracellular ROS and redox-sensitive metabolic pathways, enabling visualisation of changes in glutathione balance, NADH/NAD^+^ ratios, and oxidative stress levels within tumour cells. Similarly, pH-sensitive optical probes have been used to map extracellular acidification associated with increased glycolytic flux and poor vascular perfusion (185, 186). Although these techniques offer high sensitivity and excellent molecular specificity, their clinical translation is limited by poor tissue penetration, dependence on exogenous tracers, and predominant use in preclinical or intraoperative experimental settings rather than routine diagnostic imaging.

Magnetic resonance-based techniques have gained greater translational relevance because they can be integrated into standard clinical imaging platforms without ionising radiation. Blood oxygen level-dependent (BOLD) MRI provides an indirect measure of tissue oxygenation by detecting variations in deoxyhaemoglobin concentration (187–189), making it a useful surrogate marker for tumour hypoxia. This is particularly relevant because hypoxic tumours are known to exhibit increased resistance to radiotherapy and certain systemic therapies (190, 191), and therefore, imaging hypoxia has potential implications for treatment planning and prognostic assessment. Chemical exchange saturation transfer (CEST) MRI, including amide proton transfer imaging, extends functional imaging capability by allowing indirect assessment of tissue pH and protein metabolism through proton exchange mechanisms (192, 193). These approaches provide spatially resolved information on tumour metabolic activity and acidosis, which reflect underlying cellular proliferation rates and microenvironmental stress. More recently, emerging MRI techniques aimed at directly estimating extracellular pH have further expanded the potential to non-invasively characterise tumour acidity (194, 195), although these remain largely in the early stages of clinical validation.

From a clinical perspective, functional imaging of hypoxia, redox imbalance, and acidosis is increasingly being explored as part of a precision oncology framework. These imaging biomarkers have shown potential in identifying tumours that are more likely to exhibit aggressive behaviour or reduced responsiveness to conventional therapies (196, 197). In particular, hypoxia imaging has been associated with radiotherapy resistance, while metabolic and acidosis-related imaging signals have been linked to tumour grade, proliferative activity, and early treatment response dynamics (198, 199). Such information may support more individualised treatment strategies, including dose adaptation in radiotherapy, early modification of systemic therapy regimens, or selection of patients for hypoxia-targeted or metabolism-modulating therapies. It is crucial to note that, despite these encouraging advancements, there is still little practical application of these functional imaging methods. Inconsistent acquisition techniques, a lack of established quantitative thresholds, and insufficient validation against long-term clinical outcomes still limit their widespread usage in oncology practice. Accordingly, current evidence supports their role primarily in research and specialised clinical trials rather than established diagnostic pathways (200–202). Major guideline bodies, including ACR, NCCN, and EUSOBI, currently do not include these techniques in routine breast imaging recommendations, reflecting the need for further multicentre prospective studies to establish reproducibility, clinical utility, and cost-effectiveness.

## Oxidative stress in breast cancer

High levels of ROS have been identified in virtually all cancers, and ROS promote tumour development and progression. ROS are molecules that possess a single unpaired electron in their outermost shell. This feature makes them highly reactive. ROS can be grouped into free oxygen radicals and non-radical ROS. Examples of free oxygen radicals are superoxide, nitric oxide, organic radicals, peroxyl radicals, hydroxyl radical and sulfonyl radicals, while examples of non-radical ROS include hydrogen peroxide, singlet oxygen, ozone and hypochlorite (126, 203). ROS can be produced exogenously by ionising radiation (204), as well as xenobiotics, which can be pharmaceutical or environmental chemicals (205, 206). In the mitochondria, superoxide is produced as a byproduct of oxidative phosphorylation (207). There are risk factors for the development of breast cancer and its progression that are implicated with ROS generation to some extent (208, 209).

**Figure 8:**
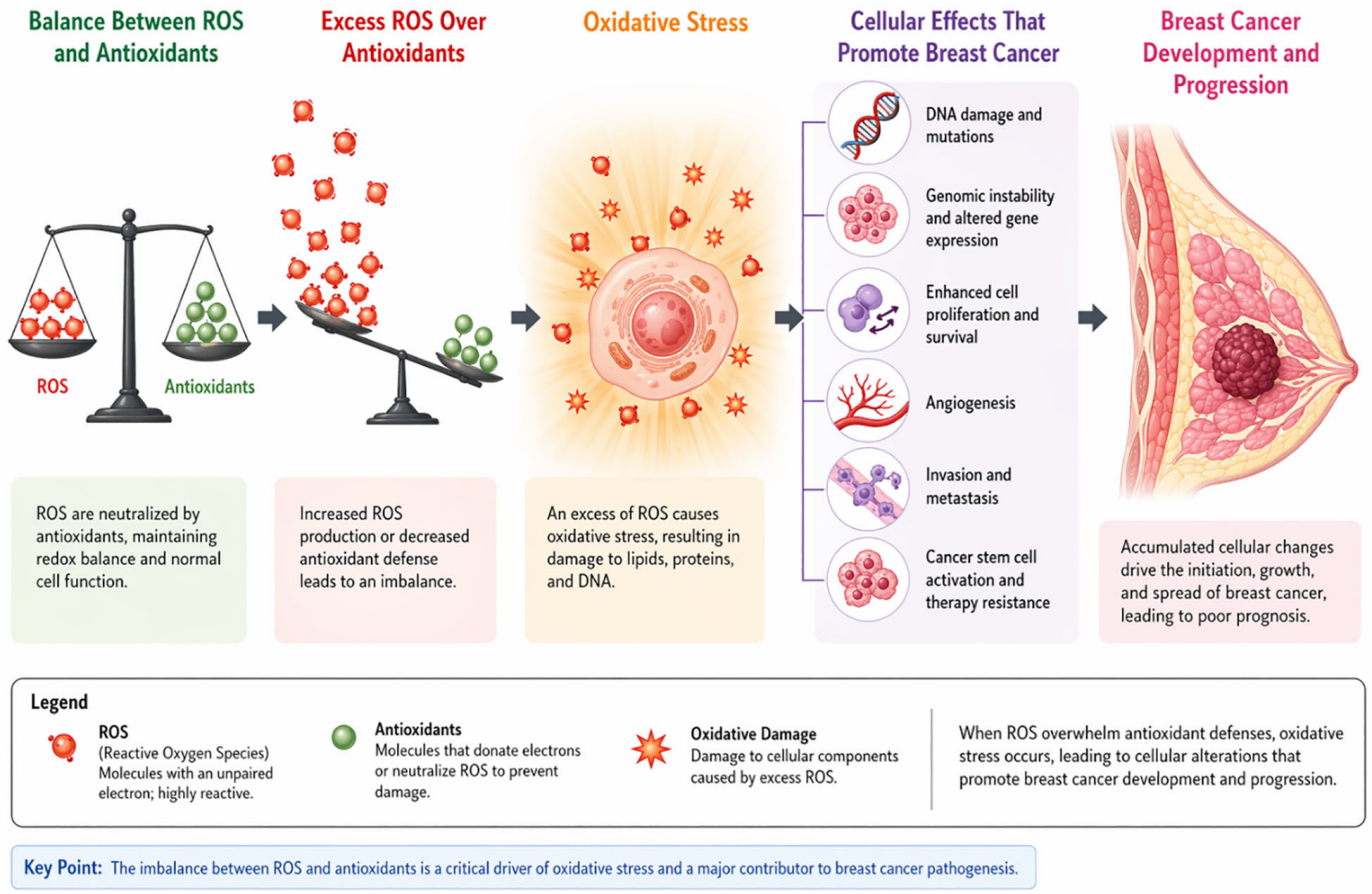
Variation between ROS and antioxidants leading to oxidative stress and breast cancer development.

The link between oxidative stress and both the initiation and progression of cancer has been shown via the ability of oxidative stress to induce DNA damage, genome instability, and cell proliferation (210, 211). An increase in oxidative stress can reduce the body’s antioxidant defence against angiogenesis and metastasis. Compounds like malondialdehyde (MDA) and hydroxyguanosine, which are created from free radicals, can be used as indicators of cancer (212, 213). Oxidative stress could lead to carcinogenesis through damage to lipid, protein or nuclear components of the cell, altered signalling pathways, genomic changes and oncogenic activation (214, 215). ROS can directly be involved in carcinogenesis through oxidation, halogenation, and nitration of nuclear DNA, lipids and ribonucleic acid (RNA) (216),indirectly by the activation of different signalling pathways (**Figure 8**) (217, 218).

Many different genes responsible for growth factors, regulatory molecules of the cell cycle, cytokines, inflammatory cytokines and anti-inflammatory molecules can be expressed through oxidatived stress induced activation of several transcription factors. They include nuclear factor kappa-light-chain-enhancer of activated B cells (NF-kB), activator protein 1 (AP-1), p53, and nuclear factor erythroid 2-related factor 2 (NRF2) (219, 220). These signalling pathways are important in sustaining cancer cells proliferation and are involved in the dissemination of inter- and intracellular information (221–223). The mitogen-activated protein (MAP) kinase/AP-1 and NF- kB pathways are highly significant pathways affected by oxidants (224, 225). Extracellular signal-regulated kinase (ERK), a MAP kinase family that modifies gene expression through the phosphorylation of a variety of transcription factors, is the most frequent pathway associated with the control of cell proliferation. Observations of the activation of ERK as a response to a change in cellular redox homeostasis have been made (226–228). Redox state can also influence NF-kB, which is linked to the control of some genes that contribute to cell transformation, proliferation and angiogenesis (229).

A principal transcription factor, NRF2, which stimulates the expression of a broad set of genes that act towards the restoration of redox balance and reduce the damage from ROS, in a large part regulates the response to oxidative stress (230, 231). Kelch-like ECH-associated protein 1 (KEAP1), an E3 ubiquitin ligase, controls NRF2 activity post-translationally. The KEAP1 protein detects oxidative stress by going through a conformational change that prevents association with NRF2, leading to NRF2 accumulation and translocation to the nucleus, which then induces transcription through antioxidant response elements found in a broad set of genes involved in redox homeostasis and correcting oxidative damage. In oxidative homeostasis, this pathway seems to be central in different cells (232–234). Somatic mutation of the NRF2 pathway genes plays a role in some human cancers (235, 236). Wild-type BRCA1 influence the transcription of some genes in the antioxidant response pathway, giving relative resistance to oxidative stress. Proof of this was shown, pointing towards increased activity of NRF2; nevertheless, NRF2 was not an obvious transcriptional target of BRCA1 (230, 237, 238). BRCA1 can interact with NRF2, and this prohibits binding and ubiquitination by KEAP1 (230, 239, 240), thus making NRF2 stable and causing downstream activation of target genes and the anti-stress response. It is important to note that in BRCA1-deficient mammary cells, activation of NRF2 via small interfering RNA inhibition of KEAP1 rescued the survival defect linked with loss of BRCA1 and caused ROS levels restoration, indicating that this mechanism is important (237, 240).

Cellular lesions occur through the oxidative modification of protein and lipid peroxidation of cell membranes (241). Lipid peroxidation, in particular, can be associated with nuclear DNA and mitochondrial injuries, ensuring an outcome of genomic alteration (242–244). Cross-linking, which causes changes in deoxyribose, is induced by the reactions of DNA with hydroxyl radicals. Thymine glycol and 8-hydroxyl guanosine (8-OHdG) are metabolites resulting from the oxidative damage of DNA. 8-hydroxy guanosine (8-OHdG) is also a main marker of oxidative stress, and its accumulation is seen in breast tumour cells; hence, it can be a tumour marker (245, 246). Thymidine phosphorylase enzyme is important in the production and promotion of ROS in carcinoma. It is greatly expressed in most breast cancer cells (247). Thymidine phosphorylase converts thymidine to thymine and 2-deoxy-D-ribose phosphate. The main cause of oxidative stress in breast cancer patients is a rise in the gene expression of thymidine phosphorylase (248, 249). Furthermore, the activity of ROS in breast cancer may not be limited to the mutagenic activity or reactions that lead to the initiation and progression of breast cancer alone. Breast cancer cells *in vivo* and *in vitro* are normally under persistent stress (250, 251). Genetic instability caused by persistent cancer cell oxidative stress will therefore lead to the increase of the malignant potential of the tumour, due to the inactivation of additional tumour suppressor genes in tumour cells, or via increasing expression of proto-oncogenes (252, 253).

## Antioxidants

An antioxidant is a stable molecule capable of donating an electron to a free radical, thereby neutralising it and reducing its potential to cause cellular damage. The protective effect of antioxidants is largely attributed to their ability to scavenge free radicals (126, 243, 254). By interacting with these reactive species, antioxidants can interrupt chain reactions before essential biomolecules are harmed (255) The human body possesses an antioxidant defence system that functions to prevent or limit oxidative damage mediated by free radicals. This system operates through several mechanisms, including direct scavenging of reactive species, metal chelation, and enzymatic processes that rapidly neutralise reactive molecules following their formation (126, 243, 256).

Enzymatic antioxidants are generally endogenous, although certain non-enzymatic antioxidants, such as coenzyme Q10, are also produced within the body. In contrast, many antioxidants are exogenous and must be obtained from the diet, as their synthesis does not occur in eukaryotic cells (215). The principal antioxidant enzymes target major ROS generated during the incomplete reduction of molecular oxygen, notably superoxide anion (O_2_^-^) and hydrogen peroxide (H_2_O_2_).

Superoxide dismutase (SOD) catalyses the conversion of superoxide radicals, while catalase (CAT) and glutathione peroxidase (GPx) detoxify hydrogen peroxide. SOD is a metalloenzyme that catalyses the dismutation of superoxide anions into hydrogen peroxide and molecular oxygen and is primarily located in the mitochondria (257, 258). Catalase, a tetrameric ferriheme oxidoreductase, facilitates the breakdown of hydrogen peroxide into water and oxygen (259) Glutathione peroxidase, a selenium-dependent oxidoreductase, utilises hydrogen peroxide or organic hydroperoxides as substrates, with reduced glutathione (GSH) acting as the electron donor (260) The reactions include: H_2_O_2_ + 2GSH → 2H_2_O + GS–SG and ROOH + 2GSH → ROH + H_2_O + GS–SG (261, 262). The GPx family consists of eight isoenzymes (GPx1-GPx8), each exhibiting distinct structural and functional characteristics (263).

Secondary antioxidant enzymes contribute indirectly by supporting the activity of primary antioxidants (264). For instance, glucose-6-phosphate dehydrogenase plays a key role in generating nicotinamide adenine dinucleotide phosphate (NADPH), which is essential for the functioning of primary antioxidant enzymes. The activity of these enzymes depends on a continuous supply of reduced molecules, particularly GSH and thioredoxin (Trx). These reductants are regenerated by NADPH-dependent enzymes such as glutathione reductase and thioredoxin reductase. In turn, these reductases rely on a steady supply of NADPH to maintain redox balance. Consequently, enzymes involved in NADPH regeneration can be considered secondary antioxidants, as their dysfunction may disrupt ROS homeostasis. Non-enzymatic antioxidants act through non-catalytic mechanisms and may be either endogenous or obtained exogenously from the diet. Endogenous examples include glutathione (γ-glutamyl-cysteinyl-glycine) and thioredoxin, both of which can effectively scavenge ROS such as hydroxyl radicals (•OH), hydrogen peroxide, and peroxynitrite (ONOO^-^) without enzymatic assistance. Dietary antioxidants include polyphenols, vitamins such as vitamins C and E, and carotenoids (261, 262).

While antioxidant systems can mitigate oxidative injury to healthy tissues during breast cancer treatment, indiscriminate use of exogenous antioxidant supplements during ROS-dependent therapies such as anthracycline chemotherapy and radiotherapy may theoretically reduce treatment efficacy by protecting malignant cells from oxidative damage. Current American Society of Clinical Oncology - Society for Integrative Oncology (ASCO-SIO) and European Society for Medical Oncology (ESMO) guidance does not support routine antioxidant supplementation during active treatment due to insufficient evidence of benefit and potential for harm. Instead, guideline-supported integrative approaches, including exercise, acupuncture, mindfulness, and nutritional optimisation, are preferred for symptom management. Antioxidant interventions may have greater relevance in survivorship or deficiency correction settings, underscoring the need for individualised, therapy-specific redox management.

## Dietary antioxidants

Nutrition is an important key in regulating oxidative stress. Inadequate and excessive intake of nutrients can disrupt ROS homeostasis in cells, affect the signalling pathways and cellular pathways of cells (265, 266). Certain nutrients or dietary components can have links with oxidative stress and even carcinogenesis. For example, alcohol causes the malfunctioning of biological signalling molecules by increasing the amount of ROS, while decreasing the levels of cellular antioxidants (267), leading to the accumulation of acetaldehyde (268) and causing cell apoptosis as a result of it inducing mitochondrial dysfunction (269). While a high carbohydrate meal can lead to an increase in oxidative stress, and caloric intake of lipids that exceeds energy expenditure leads to high production of ROS, essential fatty acids (EFAs) of the omega-3 family, in contrast to other lipids, play a defensive role from oxidative stress (270, 271) Eating fibre-rich foods may protect against oxidative stress, slightly improving the indices of inflammation and oxidative stress (272). Flavonoids modulate cellular stress by scavenging ROS through O_2_- radical inactivation and hydrogenation or complexing with oxidant species to stabilise free radicals (254, 273).

For protein consumption, there are conflicting statements. Some research findings show that high-protein intake can cause oxidative stress, which may lead to increased risk of chronic disease, including cancer, while other findings did not show any correlation between high-protein diets and long-term increase in ROS levels (271, 274, 275). Natural substances and products have long been utilised in the treatment of various diseases and have increasingly become a major focus of cancer drug discovery research. Extensive studies have demonstrated that many natural products possess anticancer properties, exerting their effects by disrupting key processes involved in carcinogenesis (**Table 4**). These processes include cancer initiation, promotion, and progression, and are regulated through mechanisms such as cell proliferation, differentiation, apoptosis, angiogenesis, and metastasis. Much attention is being paid to the usage of natural products because they can be an inexpensive option or alternative to the treatment of cancer. For example, scientific evidence has shown that the daily intake of a fruit and vegetable-rich diet decreases the risk of cancer (276). The anticancer properties of phytochemicals were shown to be associated with the activity of antioxidants (277). Apart from the direct anti-tumour properties of dietary phytochemicals, these substances can also enhance the therapeutic qualities of chemotherapeutic agents (278).

**Table 4:** Antioxidants in breast cancer: evidence, mechanisms, and therapeutic implications.

Antioxidant	Breast cancer subtype(s)	Study type	Mechanism of action	Source	References
Curcumin	General BC (*in vitro*)	Preclinical (cell, animal)	Modulates catalase and other antioxidant enzymes; induces apoptosis, cell-cycle arrest, redox signalling	Turmeric (*Curcuma longa*)	(304)
	BC stem cells; general BC	Preclinical (cell, animal); systematic reviews	Modulates NF-κB, PI3K/Akt, MAPK, JAK/STAT; induces apoptosis, anti-angiogenesis	Turmeric (*Curcuma longa*)	(305, 306)
Vitamin B6	Postmenopausal BC	Observational studies, nested case-control	Involved in amino acid metabolism; higher plasma PLP levels reduce risk	Fish, liver, potatoes, bananas	(307)
Vitamin C (ascorbate)	BC cell lines, tamoxifen/DOX/docetaxel-resistant MCF-7	Preclinical + Clinical	At high pharmacological doses, it produces H_2_O_2_ to selective oxidative stress in cancer cells; enhances chemo efficacy; reduces DOX liver toxicity	Fruits & vegetables	(308–310)
	Invasive BC (pre- and post-diagnosis)	Observational, meta-analyses	Antioxidant, reduces oxidative DNA damage; dietary intake improves survival	Citrus fruits, kiwi, strawberries, and cruciferous vegetables	(311, 312)
Vitamin D	ER+/PR+; DCIS; postmenopausal BC	RCTs (CaD trial), meta-analyses, case-control	Anti-proliferative, pro-apoptotic, modulation via VDR; deficiency linked to worse survival	Fatty fish, fish oils, fortified foods	(313–315)
Epigallocatechin-3-gallate (EGCG)	General BC; recurrence	Case-control; meta-analyses	Antioxidant, induces apoptosis, suppresses tumorigenesis	Green tea	(316, 317)
Vitamin E (tocopherols)	BC patients (cardiotoxicity prevention)	Clinical (prospective, prophylaxis); preclinical (DOX models)	Lipid-peroxide scavenger; reduces DOX-induced kidney toxicity and chemo/radio side effects	Nuts, seeds, oils	(318, 319)
Folate (vitamin B9)	HR-negative BC; *BRCA1* carriers	Observational meta-analyses; case-control; prospective cohort	DNA synthesis and methylation reduce risk in moderate alcohol consumers and *BRCA1* carriers	Dark leafy greens, legumes, nuts, liver	(320, 321)
Lactobacillus (probiotics)	Menopausal BC; general BC	Animal and human observational studies	Modulates immune response; impacts oestrogen metabolism via microbiota	Yoghurt, kefir, fermented foods	(322, 323)
β-carotene (vitamin A precursor)	Postmenopausal BC risk	Observational/Epidemiologic	Quenches singlet oxygen, a radical scavenger	Carrots, leafy greens	(324)
	General BC; survival outcomes	Observational, cohort, pooled analyses	Antioxidant, induces apoptosis, cell-cycle arrest; precursor to vitamin A	Carrots, spinach, mangoes	(325–327)
Coenzyme Q10 (CoQ10)	DOX toxicity models	Preclinical (mice, gastric mucosa); clinical discussions	Suppresses NF-κB/TNF-α signalling; reduces DOX oxidative stress injury	Endogenous + meat, fish	(328)
Sulforaphane	Hormone-dependent BC; ER+	Preclinical, epidemiological meta-analysis	Epigenetic regulation, apoptosis, and anti-inflammatory	Broccoli, kale, cabbage	(329, 330)
Indole-3-carbinol	Hormone-dependent BC	Preclinical (mouse, cell)	Alters oestrogen metabolism; induces apoptosis	Cruciferous vegetables	(331, 332)
Flavonoids/polyphenols (quercetin, flavanones, etc.)	TNBC (ROS-high), other BC	Preclinical (cell, animal)	ROS scavenging, NF-κB/Nrf2 modulation, apoptosis; dose-dependent (antioxidant at low, pro-oxidant at high)	Fruits, tea, herbs	(333, 334)
	Postmenopausal BC	Meta-analyses, case-control	Flavonol has antioxidant, anti-inflammatory, and anti-estrogenic effects	Onions, grapes, berries, broccoli	(335, 336)
n-3 Polyunsaturated fatty acids (PUFAs)	ER+, PR+; premenopausal BC	Case-control, meta-analyses	Competes with n-6 PUFAs; inhibits tumour growth and angiogenesis	Marine fish, fish oils	(337, 338)
N-acetylcysteine (NAC)	Experimental BC adjunct	Preclinical (cell)	Precursor to GSH; modulates redox	Synthetic	(339)
Lycopene	General BC	*In vitro*, observational meta-analysis	Regulates oxidative and inflammatory processes; apoptosis; inhibits angiogenesis/metastasis	Tomatoes, watermelon	(340, 341)
Piperine	Synergistic with curcumin	Preclinical (*in vitro*)	Enhances curcumin bioavailability; antioxidant; anti-proliferative	Black pepper (*Piper nigrum*)	(342)
Pristimerin (triterpenoid)	BC stem cells	Preclinical (*in vitro* + xenograft)	Apoptosis + defective autophagy in CSCs	*Celastraceae* family plants	(343)

General breast cancer (BC) refers to breast cancer studies that do not focus on a specific molecular subtype (such as ER+, HER2+, or TNBC) but instead investigate breast cancer broadly across multiple cell lines, patient groups, or experimental models. BC, breast cancer; NF-κB, nuclear factor kappa B; PLP, pyridoxal 5′-phosphate; MCF-7, Michigan Cancer Foundation-7; H_2_O_2_, hydrogen peroxide; DOX, doxorubicin; DNA, deoxyribonucleic acid; ER+, oestrogen receptor-positive; PR+, progesterone receptor-positive; DCIS, ductal carcinoma *in situ*; RCTs, randomised controlled trials; CaD, calcium plus vitamin D; VDR, vitamin D receptor; *BRCA1*, breast cancer gene 1; TNBC, triple-negative breast cancer; ROS, reactive oxygen species; Nrf2, nuclear factor erythroid 2-related factor 2; PUFAs, polyunsaturated fatty acids; GSH, glutathione; CSCs, cancer stem cells; TNF-α, tumour necrosis factor-alpha; PI3K/Akt, phosphoinositide 3-kinase/protein kinase B; MAPK, mitogen-activated protein kinase; JAK/STAT, Janus kinase/signal transducer and activator of transcription.

## Sources of dietary antioxidants

Plants produce secondary metabolites that are being investigated by scientists for anti-cancer properties that would be used to develop novel anti-cancer drugs. Previous successes in discovering anti-cancer compounds in plants that have been incorporated in cancer treatment have led to the need to use emerging technologies for further development of the area (279–281). Nanotechnology-based nanoparticles are employed to enhance the anticancer efficacy of plant-derived drugs by controlling drug release and enabling novel delivery strategies (282). Since antiquity, plants have been widely used in disease treatment due to their inherent antiseptic and therapeutic properties. Hence, research is necessary in discovering and investigating the potential properties in plants for the preparation of nanomaterial anti-cancer drugs.

### Polyphenols

Flavonoids, curcumin, tannins, resveratrol, and gallatechins are polyphenolic compounds considered to be anti-cancer agents (283). Polyphenols are natural antioxidants that can help reduce the risk of cancer. Gallatechins are found in green tea, and resveratrol is found in grapes, red wine and peanuts (284–286). Numerous studies have demonstrated that polyphenols exert cytotoxic effects on various cancer cell lines and possess significant antioxidant activity (287, 288). These compounds are believed to induce apoptosis, thereby contributing to their anticancer effects. One proposed mechanism involves the regulation of chromatin-bound copper ions, leading to oxidative DNA damage and fragmentation that triggers apoptotic pathways (289, 290). In this context, resveratrol has been shown to promote DNA degradation in the presence of Cu(II) ions (291).

Flavonoids, which represent a major class of polyphenols, constitute a large family of plant-derived secondary metabolites with approximately 10,000 identified structures. Extensive research has been conducted on flavonoid-rich plants to evaluate their anticancer potential (292–294). Purified flavonoids have been reported to exhibit significant anticancer activity, particularly against breast cancer cell lines such as Michigan Cancer Foundation-7 (MCF-7) (295, 296) Curcumin, another well-known polyphenol, is the principal bioactive constituent of *Curcuma longa* (turmeric). Extracted from the plant’s rhizome, curcumin has been widely investigated for its potential role in cancer prevention and therapy. Experimental evidence indicates that curcumin can modulate cell-cycle progression, suppress cellular proliferation, and induce apoptosis in cancer cells (297–299).

### Brassinosteroids

BRs are important for plant growth and development, and have also demonstrated promising therapeutic potential in cancer treatment. Several studies have shown that 28-homocastasterone (28-homoCS) and 24-epibrassinolide (24-epiBL), both natural brassinosteroids, display anticancer properties in diverse cancer cell models (300–303). BRs have been shown to be effective even in micromolar concentrations. They induce responses that influence apoptosis by communicating with the cell cycle [kl]. In breast cancer therapy, key molecular targets include the ER, EGFR, and HER-2, which are highly expressed in breast cancer cell lines such as MCF-7, MD Anderson-metastatic breast-468 (MDA-MB-468), T-47D, and MD Anderson-metastatic breast-231 (MDA-MB-231). BRs have been shown to interact with these receptor proteins, thereby inhibiting the proliferation of both hormone-dependent and hormone-independent breast cancer cells (140, 302). Treatment of breast cancer cell lines with 28-homocastasterone (28-homoCS) and 24-epibrassinolide (24-epiBL) has been associated with reduced expression of cyclins involved in the G1 phase of the cell cycle. The G1 phase represents a critical checkpoint at which cells either undergo repair or initiate apoptosis. While cancer cells typically evade apoptosis at this stage under untreated conditions, BRs are capable of triggering apoptotic processes during the G1 phase (302).

## Clinical evidence from randomised trials of antioxidant supplementation

Despite strong preclinical evidence linking oxidative stress and ROS to breast cancer initiation and progression, clinical translation of antioxidant supplementation has not demonstrated consistent therapeutic or preventive benefit. Evidence from large randomised controlled trials and well-conducted controlled studies indicates that systemic antioxidant supplementation does not reduce breast cancer incidence, progression, or mortality (**Table 5**). The largest prevention trials, including the Women’s Antioxidant Cardiovascular Study (WACS), evaluated long-term supplementation with vitamin C (500 mg/day), vitamin E (600 IU every other day), and β-carotene (50 mg every other day) in over 7,000 women. Across extended follow-up periods, no significant reduction in breast cancer incidence or mortality was observed, with hazard ratios approximating unity (344). These findings are consistent with earlier randomised studies, such as the Physicians’ Health Study (345) and Women’s Health Initiative dietary supplement trials (Ca/Vit D arms with cancer endpoints) (346), which similarly failed to demonstrate a protective effect against breast cancer development. Similarly, pooled analyses from cardiovascular and cancer prevention trials reported relative risks close to unity (RR = 0.94-1.04), confirming a lack of protective effect from antioxidant supplementation in the general population (345). The SU.VI.MAX study further reinforced this observation, showing no overall benefit for cancer prevention with a combination of antioxidants and trace elements. However, a sex-specific effect was observed, with reduced cancer incidence and mortality in men but not in women, potentially linked to lower baseline antioxidant status in men, particularly β-carotene levels (347).

**Table 5:** Randomised and controlled clinical trials evaluating antioxidant supplementation in breast cancer prevention, adjuvant therapy, and supportive care.

Population	Intervention	Setting (prevention/adjuvant/supportive)	Effect on breast cancer outcomes	Effect size	Safety outcomes	References
Women’s antioxidant cardiovascular study (WACS) (n=7,627 women free of cancer)	Vitamin C (500 mg/day), Vitamin E (600 IU every other day), β-carotene (50 mg every other day)	Primary prevention	No reduction in breast or total cancer incidence or mortality	HR ~1.00 (no significant effect across agents).	Generally well tolerated; no major toxicity signals reported	Lin et al. (344)
Physician’s health study/combined antioxidant RCT meta-analyses	Vitamin E, Vitamin C, β-carotene	Primary prevention	No reduction in breast cancer incidence	RR ~0.94–1.04 across antioxidants	Generally safe, but no clinical benefit	Bardia et al. (345)
Wake forest university community Clinical oncology program research base	Coenzyme Q10	Primary prevention	No effect on breast cancer incidence	Supplementation with conventional doses of CoQ10 led to sustained increases in plasma CoQ10 levels but did not result in improved self-reported fatigue or QOL after 24 weeks of treatment.	Generally safe	Lesser et al. (352)
Prospective evaluation of vitamin E for hot flashes in breast cancer survivors.	vitamin E (800 IU daily)	Prevention	Vitamin E produced a statistically significant reduction in hot flashes compared with placebo, although the clinical benefit was minimal.	Patients showed no clear preference for vitamin E over placebo (32% vs. 29%, respectively).	Generally safe, no toxicity	Barton et al. (350)
Women’s health initiative dietary supplement trials (Ca/Vit D arms with cancer endpoints)	Vitamin D + calcium (not pure antioxidants but relevant dietary supplements often co-studied)	Prevention	No effect on breast cancer incidence	RR ~1.0 (no effect)	Mild GI side effects; no cancer-specific toxicity	Manson et al. (346)
Multivitamins do not improve radiation therapy-related fatigue (results of a double-blind randomised crossover trial)	Multivitamins	Prevention	No significant changes were elicited with the use of multivitamins.	Both groups experienced decreases in general (*P* = 0.009; *P* = 0.001) and physical fatigue scores (*P* = 0.031; *P* = 0.029) at the end of the course of placebo treatment compared with the assessment prior to this treatment.	Multivitamins do not improve radiation-related fatigue in patients with breast cancer.	de Souza Fêde et al. (351)
SWOG S0221 (breast cancer patients on chemotherapy)	Multivitamins + antioxidant use (observational within RCT framework)	Adjuvant/supportive care	Antioxidant use during chemotherapy is associated with worse survival trends in some analyses	HR >1 for recurrence/mortality trends (non-significant or adverse direction depending on subgroup)	Potential concern: antioxidants may interfere with ROS-mediated chemotherapy effects	Ambrosone et al. (348)
Neoadjuvant breast cancer RCT (vitamin D adjunct studies)	Vitamin D supplementation (metabolic modulation context)	Adjuvant therapy	Improved pathological complete response (pCR) in breast cancer patients.	Vitamin D supplementation does not affect axillary pCR; a near-significant association has been observed.	Generally safe; no major toxicity reported	Özkurt et al. (349)
The SU.VI.MAX Study: a randomised, placebo-controlled trial of the health effects of antioxidant vitamins and minerals	120 mg of ascorbic acid, 30 mg of vitamin E, 6 mg of beta carotene, 100 mug of selenium, and 20 mg of zinc, or a placebo.	Primary prevention	No benefit for breast cancer prevention.	After 7.5 years, low-dose antioxidant supplementation reduced overall cancer incidence and all-cause mortality in men, but not women, likely due to men’s lower baseline antioxidant levels, particularly beta-carotene.		Hercberg et al. (347)
Women’s health initiative CaD trial	Calcium (1000 mg) plus vitamin D (400 IU) supplementation or a placebo daily	Prevention	No benefit for breast cancer prevention	The intervention group showed a reduced risk of DCIS during overall follow-up (HR = 0.82, 95% CI: 0.70–0.96) and in the postintervention phase (HR = 0.76, 95% CI: 0.61–0.94).	CaD supplementation in postmenopausal women was associated with reduced risk of DCIS, raising the possibility that consistent use of these supplements might provide long-term benefits for the prevention of DCIS.	Peila et al. (315)

WACS, Women’s Antioxidant Cardiovascular Study; RCT, randomised controlled trial; HR, hazard ratio; RR, relative risk; CI, confidence interval; CoQ10, coenzyme Q10; QOL, quality of life; GI, gastrointestinal; SWOG, Southwest Oncology Group; ROS, reactive oxygen species; pCR, pathological complete response; NST, neoadjuvant systemic therapy; DCIS, ductal carcinoma *in situ*; CaD, calcium plus vitamin D; IU, international units; mg, milligram; and μg (mug), microgram.

In addition to prevention studies, evidence from cancer-specific and mixed-population trials has raised important considerations regarding antioxidant use during active treatment. In the SWOG S0221 trial and related analyses, the use of antioxidant supplements during chemotherapy was associated with potential trends toward worse clinical outcomes, including reduced disease-free and overall survival in exploratory analyses (348). Although not definitively causal, these findings support the biological concern that exogenous antioxidants may attenuate ROS-mediated cytotoxic mechanisms induced by chemotherapy and radiotherapy, potentially reducing treatment efficacy. In contrast, certain antioxidants and related micronutrients show context-dependent or subtype-specific effects. Vitamin D supplementation, particularly in neoadjuvant settings, has demonstrated improvements in pathological complete response (pCR) rates in breast cancer patients, although effects on axillary pCR remain inconclusive (349). Likewise, the Women’s Health Initiative calcium plus vitamin D (CaD) trial reported no overall reduction in breast cancer incidence but observed a modest reduction in ductal carcinoma *in situ* (DCIS), suggesting potential long-term preventive effects under sustained supplementation (315).

Supportive care outcomes also present mixed findings. Vitamin E supplementation in breast cancer survivors produced a statistically significant reduction in hot flashes (350); however, the clinical effect was marginal and patient preference did not differ from placebo. Multivitamin supplementation similarly showed no improvement in radiation-related fatigue (351), while observational data from chemotherapy cohorts (e.g., SWOG S0221) raised concerns that concurrent antioxidant use during treatment may negatively influence survival, potentially by attenuating ROS-mediated cytotoxicity of chemotherapy (348) Coenzyme Q10, despite increasing plasma antioxidant levels, did not improve fatigue or quality of life outcomes, further highlighting the disconnect between biochemical antioxidant enhancement and clinical benefit (352) Although antioxidants exhibit important biological activity, supplementation has not consistently demonstrated significant benefits in breast cancer prevention or treatment, with outcomes varying according to clinical context, timing of use, and patient subgroup.

## Mechanisms through which dietary antioxidants counteract oxidative stress

**Figure 9:**
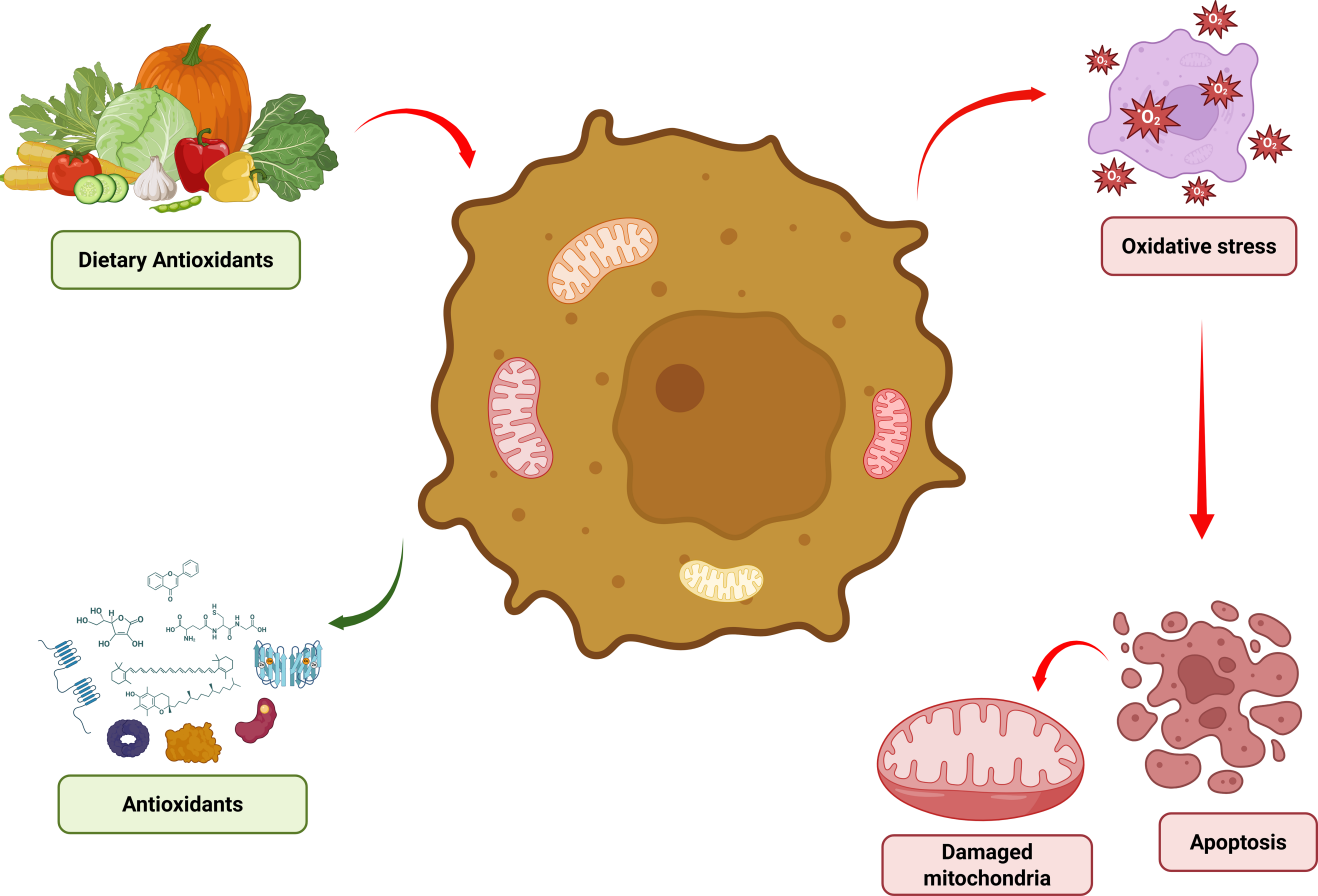
Mechanisms through which dietary antioxidants counteract oxidative stress in the cancerous state.

The maintenance of an adequate level of antioxidants in the body of an organism can be obtained from consuming dietary antioxidants (126, 353). Antioxidants are more effective and specific in shielding cells from radicals than DNA repair enzymes. Glutathione, a major endogenous antioxidant, helps in the protection of cells from ROS such as peroxides (354). Cellular integrity under normal physiological conditions depends on the tight regulation of intracellular ROS levels. This redox balance is preserved through the rapid detection and neutralisation of ROS by non-enzymatic antioxidants such as glutathione, dietary antioxidants including flavonoids and vitamins A, C, and E, as well as antioxidant enzymes that target specific ROS species (355). Antioxidants are generally thought to function through two principal mechanisms (356) The first is a chain-breaking mechanism, in which a primary antioxidant donates an electron to stabilise a free radical. The second involves secondary antioxidants, which act by eliminating reactive oxygen or nitrogen species initiators through quenching of chain-initiating catalysts, thereby preventing the propagation of oxidative reactions (**Figure 9**) (126, 356, 357).

## Exploration of studies linking dietary antioxidants to breast cancer prevention

Bioactive molecules obtained from plants have received attention in recent times, given their therapeutic importance in preventing and treating illnesses. Daily intake of a wide variety of phytochemicals has been shown to possess chemopreventive properties (358) Chemoprevention of cancer involves the inhibition of carcinogenesis by the administration of synthetic and natural agents, and these chemopreventive agents can be broadly grouped into three groups (126). They include;

- In this group, chemopreventive agents prevent the formation of procarcinogens from the precursor components. This is illustrated in vitamin C, which does not allow the formation of nitroso compounds (359).
- In this group, chemopreventive agents act as blocking agents that prevent carcinogenic compounds from interacting with the cellular target. They include isothiocyanates, flavones and phenols. Blocking by these agents can be achieved by restricting the activation of carcinogens to their carcinogenic form, inducing the enzyme system that can detoxify carcinogens, and by reacting with carcinogens and stopping their reaction with the cellular targets (359, 360).
- In this group, chemopreventive agents act as suppressing agents that prevent carcinogenesis by their ability to suppress the process; examples include protease inhibitors and retinoic acid (361).

Pomegranate *(Punica granatum* L.) is a fruit known to contain high amounts of antioxidants, and as a result, may be used as a prophylactic and therapy for cancer. In light of this, Ferrante et al. (362) investigated the biological effects of pomegranate on cellular redox state, proliferation and metabolism in the breast cancer cell line MDA-MB-231, and since it is known that the production of ROS and regulation of oxidative metabolism in the mitochondria play a part in the formation of tumours. Experimental findings demonstrated that treatment with fresh pomegranate juice significantly reduced intracellular ROS levels, even at the lowest concentration tested. In addition, pomegranate juice enhanced mitochondrial respiration and, at higher concentrations, suppressed glycolytic activity while simultaneously inhibiting cell proliferation. Given the seasonal nature of pomegranate, optimal storage conditions capable of preserving its bioactive properties were also evaluated (363). Storage under a controlled atmosphere for 30 days was found to maintain the ability of pomegranate juice to enhance mitochondrial respiration to a level comparable with that of freshly prepared juice. In contrast, freezing, although effective in preserving antioxidant activity and anti-proliferative effects, produced metabolic responses opposite to those observed with the fresh extract. These findings support the preventive and therapeutic potential of pomegranate juice in cancer management (364).

The strong antioxidant activity of pomegranate juice has been largely attributed to ellagitannins, particularly punicalagin, whose concentrations are approximately threefold higher than those found in green tea infusions or red wine (365). Pomegranate-derived compounds have been shown to modulate a wide range of molecular targets, including transcription factors, pro- and anti-apoptotic proteins, cell cycle regulators, protein kinases, cell adhesion molecules, pro-inflammatory mediators, and growth factors across multiple cancer types, such as skin, breast, prostate, colon, and lung cancers (366, 367). Recent studies further indicate that pomegranate can attenuate aggressive cancer phenotypes by reducing breast cancer cell invasion and motility (366, 368) diminishing cancer-associated inflammation (369)and suppressing cellular proliferation, effects that are likely linked to its antioxidant capacity (370).

## Examination of the potential protective effects of specific antioxidants

Oxidative stress-mediated regulation of PTP1B has been implicated in breast cancer development through its role in pro-oncogenic signalling pathways. Green tea-derived catechins, specifically epigallocatechin and epigallocatechin gallate, have been shown to suppress PTP1B activity and inhibit the proliferation of MCF-7 breast cancer cells (371). These findings suggest that such catechins may be exploited to enhance the effectiveness of systemic anticancer therapies. Additionally, they hold promise as supplementary agents for mitigating oxidative stress-related side effects associated with conventional anticancer drugs (371).

## Discussion of conflicting research and areas requiring future investigations

Many chemotherapeutic approaches are designed to elevate intracellular ROS levels to induce irreparable cellular damage, ultimately triggering apoptosis in tumour cells (372). However, the use of antioxidants in combination with such therapies may be counterproductive when the anticancer mechanism relies on ROS-mediated cell death. In contrast, combining antioxidants with treatments that induce apoptosis through ROS-independent pathways may provide synergistic benefits and improve therapeutic outcomes. Depending on the strategy to be used to design an anticancer treatment, there is a need for further studies in this area to clearly identify antioxidants that produce the appropriate biological activity against cancer cells with regard to oxidative stress (373).

## Emerging trends that may influence the better delivery of breast cancer drugs to the target sites

Nanotechnology uses nanoparticles to deliver drugs to target sites. Bromelain from *Ananas comosus* demonstrates greater anticancer efficacy when formulated within nanoparticles than when administered in its unmodified, free form (374). Encapsulated nanoparticles can help to solve the bioavailability limitation in biological systems, which occurs when sufficient concentrations of these compounds are not reached in the systemic circulation after eating or ingesting the compounds to express an antitumor effect (280, 375).

## Conclusion

Among women around the world, breast cancer accounts for the highest number of cancer-related deaths. Oxidative stress, as a result of ROS imbalance, has been discovered in many studies to be responsible for the initiation and progression of carcinogenesis. ROS may cause carcinogenesis by inducing DNA degradation, angiogenesis, cell cycle disruption, and metastasis. Although various forms of treatments that include mastectomy, chemotherapy, and radiotherapy have been used in the treatment of breast cancer, researchers found a need to develop treatment alternatives with better efficacy and fewer side effects. This quest led a lot of researchers to explore antioxidants, which can mop up the effects of ROS and are found as natural products in fruits, vegetables and medicinal plants. Various antioxidants like ellagitanin and punicalagin from pomegranate, gallatechins from green tea and curcumin from turmeric have been observed to have positive effects against breast cancer. Since there is evidence of counterproductive effects of certain antioxidants, there is a need to understand why some antioxidants are effective against breast cancer cells and why others do not seem to have any effect regarding oxidative stress. Due to the anti-cancer preventive properties of dietary antioxidants, diets like fruits and vegetables should be promoted among breast cancer patients.

## References

[1] Zhang Y Ji Y Liu S Li J Wu J Jin Q . Global burden of female breast cancer: new estimates in 2022, temporal trend and future projections up to 2050 based on the latest release from GLOBOCAN. J Natl Cancer Center. (2025) 5:287–96. doi: 10.1016/J.JNCC.2025.02.002 40693239 PMC12276554

[2] Bray F Laversanne M Sung H Ferlay J Siegel RL Soerjomataram I . Global cancer statistics 2022: GLOBOCAN estimates of incidence and mortality worldwide for 36 cancers in 185 countries. CA Cancer J Clin. (2024) 74:229–63. doi: 10.3322/CAAC.21834 38572751

[3] Jezierska-Drutel A Rosenzweig SA Neumann CA . Role of oxidative stress and the microenvironment in breast cancer development and progression. (2013) 119:107–25. doi: 10.1016/B978-0-12-407190-2.00003-4 PMC3950899 23870510

[4] Sariego J . Breast cancer in the young patient. Am Surg. (2010) 76:1397–400. doi: 10.1177/000313481007601226 21265355

[5] Antoniou AC Easton DF . Models of genetic susceptibility to breast cancer. Oncogene. (2006) 25:5898–905. doi: 10.1038/sj.onc.1209879 16998504

[6] Lee E McKean-Cowdin R Ma H Spicer DV Van Den Berg D Bernstein L . Characteristics of triple-negative breast cancer in patients with a BRCA1 mutation: results from a population-based study of young women. J Clin Oncol. (2011) 29:4373–80. doi: 10.1200/JCO.2010.33.6446 22010008 PMC3221522

[7] McPherson K Steel CM Dixon JM . ABC of breast diseases. Breast cancer-epidemiology, risk factors, and genetics. Bmj. (2000) 321:624–8. doi: 10.1136/bmj.321.7261.624 10977847 PMC1118507

[8] Wang J Wu S-G . Breast cancer: an overview of current therapeutic strategies, challenge, and perspectives. Breast Cancer (Dove Med Press). (2023) 15:721–30. doi: 10.2147/BCTT.S432526 37881514 PMC10596062

[9] Goldwyn RM . Breast reconstruction after mastectomy. N Engl J Med. (1987) 317:1711–4. doi: 10.1056/NEJM198712313172706 3320749

[10] Medina M Quesada A . Dietary proteins and angiogenesis. Nutrients. (2014) 6:371–81. doi: 10.3390/nu6010371 24445377 PMC3916867

[11] Wild CP Weiderpass E Stewart BW . World Cancer Report: Cancer research for cancer prevention. Lyon, France: International Agency for Research on Cancer (2020). 39432694

[12] Sung H Ferlay J Siegel RL Laversanne M Soerjomataram I Jemal A . Global cancer statistics 2020: GLOBOCAN estimates of incidence and mortality worldwide for 36 cancers in 185 countries. CA Cancer J Clin. (2021) 71:209–49. doi: 10.3322/caac.21660 33538338

[13] Ferlay J Colombet M Soerjomataram I Parkin DM Piñeros M Znaor A . Cancer statistics for the year 2020: an overview. Int J Cancer. (2021) 149:778–89. doi: 10.1002/ijc.33588 33818764

[14] World Health Organization . Breast cancer (2025). Available online at: https://www.who.int/news-room/fact-sheets/detail/breast-cancer (Accessed August 27, 2025).

[15] Arnold M Morgan E Rumgay H Mafra A Singh D Laversanne M . Current and future burden of breast cancer: global statistics for 2020 and 2040. Breast. (2022) 66:15–23. doi: 10.1016/j.breast.2022.08.010 36084384 PMC9465273

[16] World Health Organisation . Global cancer burden growing, amidst mounting need for services (2024). Available online at: https://www.who.int/news/item/01-02-2024-global-cancer-burden-growing--amidst-mounting-need-for-services (Accessed May 4, 2026).

[17] Kalita M M D Saha I Chakrabarti A . Global burden of cancer pattern in 2020 & prediction to 2040 among older adults. Indian J Med Res. (2024) 160:397–406. doi: 10.25259/ijmr_1729_23 39737507 PMC11683504

[18] Wilkinson L Gathani T . Understanding breast cancer as a global health concern. Br J Radiol. (2022) 95:20211033. doi: 10.1259/bjr.20211033 34905391 PMC8822551

[19] Greenwald P . Role of dietary fat in the causation of breast cancer: point. Cancer Epidemiol Biomarkers Prev. (1999) 8:3–7. 9950233

[20] Mentella MC Scaldaferri F Ricci C Gasbarrini A Miggiano GAD . Cancer and Mediterranean diet: a review. Nutrients. (2019) 11:2059. doi: 10.3390/nu11092059 31480794 PMC6770822

[21] Colditz GA Atwood KA Emmons K Monson RR Willett WC Trichopoulos D . Harvard report on cancer prevention volume 4: Harvard cancer risk index. Cancer Causes Control. (2000) 11:477–88. doi: 10.1023/A:1008984432272 10880030

[22] Caprara G Pallavi R Sanyal S Pelicci PG . Dietary restrictions and cancer prevention: state of the art. Nutrients. (2025) 17:503. doi: 10.3390/nu17030503 39940361 PMC11820753

[23] Mittelman SD . The role of diet in cancer prevention and chemotherapy efficacy. Annu Rev Nutr. (2020) 40:273–97. doi: 10.1146/annurev-nutr-013120-041149 32543948 PMC8546934

[24] Singh S Sharma B Kanwar SS Kumar A . Lead phytochemicals for anticancer drug development. Front Plant Sci. (2016) 7. doi: 10.3389/fpls.2016.01667 27877185 PMC5099879

[25] Mollaei M Hassan ZM Khorshidi F Langroudi L . Chemotherapeutic drugs: cell death- and resistance-related signaling pathways. Are they really as smart as the tumor cells? Transl Oncol. (2021) 14:101056. doi: 10.1016/j.tranon.2021.101056 33684837 PMC7938256

[26] Wang S Guo S Guo J Du Q Wu C Wu Y . Cell death pathways: molecular mechanisms and therapeutic targets for cancer. Medcomm (Beijing). (2024) 5(9):e693. doi: 10.1002/mco2.693 39239068 PMC11374700

[27] Maser RS DePinho RA . Connecting chromosomes, crisis, and cancer. Sci (1979). (2002) 297:565–9. doi: 10.1126/science.297.5581.565 12142527

[28] Dakal TC Dhabhai B Pant A Moar K Chaudhary K Yadav V . Oncogenes and tumor suppressor genes: Functions and roles in cancers. MedComm. (2024) 5(6):e582. doi: 10.1002/mco2.582 38827026 PMC11141506

[29] Zhu L Lu Z Zhao H . Antitumor mechanisms when pRb and p53 are genetically inactivated. Oncogene. (2014) 34:4547. doi: 10.1038/ONC.2014.399 25486431 PMC4459916

[30] Joyce C Rayi A Kasi A . Tumor-suppressor genes. In: Statpearls. Treasure Island (Florida): StatPearls Publishing (2023). Available online at: https://www.ncbi.nlm.nih.gov/books/NBK532243/ (Accessed August 31, 2025). 30335276

[31] Hernández Borrero LJ El-Deiry WS . Tumor suppressor p53: biology, signaling pathways, and therapeutic targeting. Biochim Biophys Acta (BBA) - Rev Cancer. (2021) 1876:188556. doi: 10.1016/J.BBCAN.2021.188556 33932560 PMC8730328

[32] Wang H Guo M Wei H Chen Y . Targeting p53 pathways: mechanisms, structures and advances in therapy. Signal Transduction Targeted Ther. (2023) 8:1–35. doi: 10.1038/s41392-023-01347-1 36859359 PMC9977964

[33] Farooq Z Wani S Ragunathrao VAB Kochhar R Anwar M Farooq Z . p53 tumor suppressor: functional regulation and role in gene therapy. In: p53 - A Guardian of the Genome and Beyond. United Kingdom (London): IntechOpen (2022). doi: 10.5772/INTECHOPEN.105029

[34] Marei HE Althani A Afifi N Hasan A Caceci T Pozzoli G . p53 signaling in cancer progression and therapy. Cancer Cell Int. (2021) 21:1–15. doi: 10.1186/S12935-021-02396-8 34952583 PMC8709944

[35] Tonnessen-Murray CA Lozano G Jackson JG . The regulation of cellular functions by the p53 protein: cellular senescence. Cold Spring Harb Perspect Med. (2017) 7:a026112. doi: 10.1101/CSHPERSPECT.A026112 27881444 PMC5287062

[36] Abuetabh Y Wu HH Chai C Al Yousef H Persad S Sergi CM . DNA damage response revisited: the p53 family and its regulators provide endless cancer therapy opportunities. Exp Mol Med. (2022) 54:1658–69. doi: 10.1038/s12276-022-00863-4 36207426 PMC9636249

[37] Levine AJ . The many faces of p53: something for everyone. J Mol Cell Biol. (2019) 11:524. doi: 10.1093/JMCB/MJZ026 30925588 PMC6736316

[38] Freed-Pastor WA Prives C . Mutant p53: one name, many proteins. Genes Dev. (2012) 26:1268. doi: 10.1101/GAD.190678.112 22713868 PMC3387655

[39] Marvalim C Datta A Lee SC . Role of p53 in breast cancer progression: an insight into p53 targeted therapy. Theranostics. (2023) 13:1421. doi: 10.7150/THNO.81847 36923534 PMC10008729

[40] Yousuf A Khan NU . Targeting MDM2-p53 interaction for breast cancer therapy. Oncol Res. (2025) 33:851. doi: 10.32604/OR.2025.058956 40191734 PMC11964874

[41] Wang L Han H Dong L Wang Z Qin Y . Function of p21 and its therapeutic effects in esophageal cancer (review). Oncol Lett. (2020) 21:136. doi: 10.3892/ol.2020.12397 33552255 PMC7798030

[42] Al Bitar S Gali-Muhtasib H . The role of the cyclin dependent kinase inhibitor p21cip1/waf1 in targeting cancer: molecular mechanisms and novel therapeutics. Cancers (Basel). (2019) 11:1475. doi: 10.3390/CANCERS11101475 31575057 PMC6826572

[43] Chimento A De Luca A Avena P De Amicis F Casaburi I Sirianni R . Estrogen receptors-mediated apoptosis in hormone-dependent cancers. Int J Mol Sci. (2022) 23:1242. doi: 10.3390/IJMS23031242 35163166 PMC8835409

[44] Coates AS Millar EKA O’Toole SA Molloy TJ Viale G Goldhirsch A . Prognostic interaction between expression of p53 and estrogen receptor in patients with node-negative breast cancer: results from IBCSG Trials VIII and IX. Breast Cancer Res. (2012) 14:1–12. doi: 10.1186/bcr3348 PMC4053129 23127292

[45] Ali T Kamath SM . Correlation of P53 expression with clinicopathological parameters, hormone receptors and HER 2 Neu status in breast carcinoma. Asian Pacific J Cancer Biol. (2022) 7:307–14. doi: 10.31557/APJCB.2022.7.4.307-314

[46] Corral D Ansaldo E Delaleu J Pichler AC Kabat J Oguz C . Mammary intraepithelial lymphocytes promote lactogenesis and offspring fitness. Cell. (2025) 188:1662–1680.e24. doi: 10.1016/J.CELL.2025.01.028 39954680 PMC12117524

[47] Vogler M Braun Y Smith VM Westhoff MA Pereira RS Pieper NM . The BCL2 family: from apoptosis mechanisms to new advances in targeted therapy. Signal Transduction Targeted Ther. (2025) 10:1–31. doi: 10.1038/s41392-025-02176-0 40113751 PMC11926181

[48] Kaloni D Diepstraten ST Strasser A Kelly GL . BCL-2 protein family: attractive targets for cancer therapy. Apoptosis. (2022) 28:20. doi: 10.1007/S10495-022-01780-7 36342579 PMC9950219

[49] Williams MM Cook RS . Bcl-2 family proteins in breast development and cancer: could Mcl-1 targeting overcome therapeutic resistance? Oncotarget. (2015) 6:3519. doi: 10.18632/ONCOTARGET.2792 25784482 PMC4414133

[50] Medh RD Thompson EB . Hormonal regulation of physiological cell turnover and apoptosis. Cell Tissue Res. (2000) 301:101. doi: 10.1007/S004419900159 10928284 PMC2763512

[51] Limana F Urbanek K Chimenti S Quaini F Leri A Kajstura J . bcl-2 overexpression promotes myocyte proliferation. Proc Natl Acad Sci USA. (2002) 99:6257. doi: 10.1073/PNAS.092672899 11983915 PMC122936

[52] García-Sáez AJ . The secrets of the Bcl-2 family. Cell Death Differ. (2012) 19:1733. doi: 10.1038/CDD.2012.105 22935609 PMC3469065

[53] Qian S Wei Z Yang W Huang J Yang Y Wang J . The role of BCL-2 family proteins in regulating apoptosis and cancer therapy. Front Oncol. (2022) 12:985363. doi: 10.3389/FONC.2022.985363 36313628 PMC9597512

[54] Yu X Zhang X Dhakal IB Beggs M Kadlubar S Luo D . Induction of cell proliferation and survival genes by estradiol-repressed microRNAs in breast cancer cells. BMC Cancer. (2012) 12:29. doi: 10.1186/1471-2407-12-29 22260523 PMC3274428

[55] Akl H Vervloessem T Kiviluoto S Bittremieux M Parys JB De Smedt H . A dual role for the anti-apoptotic Bcl-2 protein in cancer: Mitochondria versus endoplasmic reticulum. Biochim Biophys Acta (BBA) - Mol Cell Res. (2014) 1843:2240–52. doi: 10.1016/J.BBAMCR.2014.04.017 24768714

[56] Kawiak A Kostecka A . Regulation of Bcl-2 family proteins in estrogen receptor-positive breast cancer and their implications in endocrine therapy. Cancers (Basel). (2022) 14:279. doi: 10.3390/CANCERS14020279 35053443 PMC8773933

[57] Aeri A Sunil Kumar BV Gupta K Kumar A Sethi RS . Expression of BCL2 associated athanogene-1 is up-regulated in canine Malignant mammary tumors. Discover Anim. (2025) 2:1–12. doi: 10.1007/S44338-025-00060-3 30311153

[58] Kizilboga T Baskale EA Yildiz J Akcay IM Zemheri E Can ND . Bag-1 stimulates Bad phosphorylation through activation of Akt and Raf kinases to mediate cell survival in breast cancer. BMC Cancer. (2019) 19(1):1254. doi: 10.1186/s12885-019-6477-4 31883527 PMC6935482

[59] Piccart MJ Di Leo A Hamilton A . HER2. a 'predictive factor' ready to use in the daily management ofbreast cancer patients? Eur J Cancer. (2000) 36(14):1755–61. doi: 10.1016/s0959-8049(00)00162-3 10974622

[60] Cheng X . A comprehensive review of HER2 in cancer biology and therapeutics. Genes (Basel). (2024) 15:903. doi: 10.3390/GENES15070903 39062682 PMC11275319

[61] Pan L Li J Xu Q Gao Z Yang M Wu X . HER2/PI3K/AKT pathway in HER2-positive breast cancer a review. Med (United States). (2024) 103:e38508. doi: 10.1097/MD.0000000000038508 38875362 PMC11175886

[62] Sivaganesh V Sivaganesh V Scanlon C Iskander A Maher S Lê T . Protein tyrosine phosphatases: Mechanisms in cancer. Int J Mol Sci. (2021) 22:12865. doi: 10.3390/IJMS222312865 34884670 PMC8657787

[63] Walston H Iness AN Litovchick L . DREAM on: Cell cycle control in development and disease. Annu Rev Genet. (2021) 55:309–29. doi: 10.1146/ANNUREV-GENET-071819-103836 34496610

[64] Liu T Song S Wang X Hao J . Small-molecule inhibitors of breast cancer-related targets: Potential therapeutic agents for breast cancer. Eur J Med Chem. (2021) 210:112954. doi: 10.1016/j.ejmech.2020.112954 33158576

[65] Ouellette MM Zhou S Yan Y . Cell signaling pathways that promote radioresistance of cancer cells. Diagnostics. (2022) 12:656. doi: 10.3390/DIAGNOSTICS12030656 35328212 PMC8947583

[66] Pesch AM Hirsh NH Michmerhuizen AR Jungles KM Wilder-Romans K Chandler BC . RB expression confers sensitivity to CDK4/6 inhibitor–mediated radiosensitization across breast cancer subtypes. JCI Insight. (2022) 7(3):e154402. doi: 10.1172/jci.insight.154402 34932500 PMC8855810

[67] Jung M Mertens C Tomat E Brüne B . Iron as a central player and promising target in cancer progression. Int J Mol Sci. (2019) 20:273. doi: 10.3390/IJMS20020273 30641920 PMC6359419

[68] Bayanbold K Singhania M Fath MA Searby CC Stolwijk JM Henrich JB . Depletion of labile iron induces replication stress and enhances responses to chemoradiation in non-small-cell lung cancer. Antioxidants. (2023) 12(11):2005. doi: 10.3390/antiox12112005 38001858 PMC10669787

[69] Lv H Shang P . The significance, trafficking and determination of labile iron in cytosol, mitochondria and lysosomes. Metallomics. (2018) 10:899–916. doi: 10.1039/C8MT00048D 29923582

[70] Imoto S Sawamura T Shibuya Y Kono M Ohbuchi A Suzuki T . Labile iron, ROS, and cell death are prominently induced by haemin, but not by non-transferrin-bound iron. Transfus Apheresis Sci. (2022) 61(2):103319. doi: 10.1016/j.transci.2021.103319 34801431

[71] Pinnix ZK Miller LD Wang W D’Agostino R Kute T Willingham MC . Ferroportin and iron regulation in breast cancer progression and prognosis. Sci Transl Med. (2010) 2:43ra56. doi: 10.1126/SCISIGNAL.3001127 PMC3734848 20686179

[72] Marques O Porto G Rêma A Faria F Cruz Paula A Gomez-Lazaro M . Local iron homeostasis in the breast ductal carcinoma microenvironment. BMC Cancer. (2016) 16:187. doi: 10.1186/s12885-016-2228-y 26944411 PMC4779214

[73] Fontana F Esser AK Egbulefu C Karmakar P Su X Allen JS . Transferrin receptor in primary and metastatic breast cancer: Evaluation of expression and experimental modulation to improve molecular targeting. PloS One. (2023) 18:e0293700. doi: 10.1371/journal.pone.0293700 38117806 PMC10732420

[74] Gong Y Ji P Yang Y-S Xie S Yu T-J Xiao Y . Metabolic-pathway-based subtyping of triple-negative breast cancer reveals potential therapeutic targets. Cell Metab. (2021) 33:51–64.e9. doi: 10.1016/j.cmet.2020.10.012 33181091

[75] Wang Y Sun Y Wang F Wang H Hu J . Ferroptosis induction via targeting metabolic alterations in triple-negative breast cancer. Biomedicine Pharmacotherapy. (2023) 169:115866. doi: 10.1016/j.biopha.2023.115866 37951026

[76] Cruz-Gregorio A . Mitochondrial redox vulnerabilities in triple-negative breast cancer: Integrative perspectives and emerging therapeutic strategies. Metabolites. (2026) 16:60. doi: 10.3390/metabo16010060 41590668 PMC12844444

[77] Ayala A Muñoz MF Argüelles S . Lipid peroxidation: Production, metabolism, and signaling mechanisms of malondialdehyde and 4-hydroxy-2-nonenal. Oxid Med Cell Longev. (2014) 2014:360438. doi: 10.1155/2014/360438 24999379 PMC4066722

[78] Cao H Xiong S-F Dong L-L Dai Z-T . Study on the mechanism of lipid peroxidation induced by carbonate radicals. Molecules. (2024) 29:1125. doi: 10.3390/molecules29051125 38474637 PMC10934600

[79] Montero-León L Cruz-Gregorio A Hernández-Cruz EY Sciutto E Fragoso G Pedraza-Chaverri J . Unlocking ferroptosis: A novel link between triple-negative breast cancer and immune regulation. Arch Biochem Biophys. (2026) 779:110760. doi: 10.1016/j.abb.2026.110760 41662919

[80] Xie L-H Fefelova N Pamarthi SH Gwathmey JK . Molecular mechanisms of ferroptosis and relevance to cardiovascular disease. Cells. (2022) 11:2726. doi: 10.3390/cells11172726 36078133 PMC9454912

[81] Chen F Kang R Tang D Liu J . Ferroptosis: Principles and significance in health and disease. J Hematol Oncol. (2024) 17:41. doi: 10.1186/s13045-024-01564-3 38844964 PMC11157757

[82] Dixon SJ Lemberg KM Lamprecht MR Skouta R Zaitsev EM Gleason CE . Ferroptosis: An iron-dependent form of nonapoptotic cell death. Cell. (2012) 149:1060–72. doi: 10.1016/j.cell.2012.03.042 22632970 PMC3367386

[83] Yang WS Stockwell BR . Ferroptosis: Death by lipid peroxidation. Trends Cell Biol. (2016) 26:165–76. doi: 10.1016/j.tcb.2015.10.014 26653790 PMC4764384

[84] Kang R Tang D . Autophagy and ferroptosis—what is the connection? Curr Pathobiol Rep. (2017) 5:153–9. doi: 10.1007/s40139-017-0139-5 29038744 PMC5640172

[85] Hu C Nydes M Shanley KL Morales Pantoja IE Howard TA Bizzozero OA . Reduced expression of the ferroptosis inhibitor glutathione peroxidase‐4 in multiple sclerosis and experimental autoimmune encephalomyelitis. J Neurochem. (2019) 148:426–39. doi: 10.1111/jnc.14604 30289974 PMC6347488

[86] Wei J Zhu L . The role of glutathione peroxidase 4 in the progression, drug resistance, and targeted therapy of non-small cell lung cancer. Oncol Res. (2025) 33:863–72. doi: 10.32604/or.2024.054201 40191731 PMC11964886

[87] Lee J Roh JL . SLC7A11 as a gateway of metabolic perturbation and ferroptosis vulnerability in cancer. Antioxidants. (2022) 11:2444. doi: 10.3390/ANTIOX11122444 36552652 PMC9774303

[88] Bersuker K Hendricks JM Li Z Magtanong L Ford B Tang PH . The CoQ oxidoreductase FSP1 acts parallel to GPX4 to inhibit ferroptosis. Nature. (2019) 575:688–92. doi: 10.1038/S41586-019-1705-2 31634900 PMC6883167

[89] Doll S Freitas FP Shah R Aldrovandi M da Silva MC Ingold I . FSP1 is a glutathione-independent ferroptosis suppressor. Nature. (2019) 575:693–8. doi: 10.1038/s41586-019-1707-0 31634899

[90] Jiang Y Zhang M Sun M . ACSL4 at the helm of the lipid peroxidation ship: a deep-sea exploration towards ferroptosis. Front Pharmacol. (2025) 16:1594419. doi: 10.3389/FPHAR.2025.1594419 40932861 PMC12417525

[91] Lee J Seo Y Roh J-L . Membrane stress and ferroptosis: Lipid dynamics in cancer. Int J Mol Sci. (2026) 27:690. doi: 10.3390/IJMS27020690 41596341 PMC12840980

[92] Viswanathan VS Ryan MJ Dhruv HD Gill S Eichhoff OM Seashore-Ludlow B . Dependency of a therapy-resistant state of cancer cells on a lipid peroxidase pathway. Nature. (2017) 547:453–7. doi: 10.1038/NATURE23007 28678785 PMC5667900

[93] Fang K Xu Z Jiang S Yan C Tang D Huang Y . Integrated profiling uncovers prognostic, immunological, and pharmacogenomic features of ferroptosis in triple-negative breast cancer. Front Immunol. (2022) 13. doi: 10.3389/fimmu.2022.985861 36505498 PMC9732280

[94] Desterke C Xiang Y Elhage R Duruel C Chang Y Hamaï A . Ferroptosis inducers upregulate PD-L1 in recurrent triple-negative breast cancer. Cancers (Basel). (2023) 16:155. doi: 10.3390/cancers16010155 38201582 PMC10778345

[95] Bottoni L Minetti A Realini G Pio E Giustarini D Rossi R . NRF2 activation by cysteine as a survival mechanism for triple-negative breast cancer cells. Oncogene. (2024) 43:1701–13. doi: 10.1038/s41388-024-03025-0 38600165 PMC11136656

[96] Antmen SE Yalaza C Tuncel F Berkesoglu M Erden Erturk S Canacankatan N . Triple-negative breast cancer and ferroptosis: Expression profiling of key regulatory genes. Arch Med Sci. (2025) 21(5):2079–87. doi: 10.5114/aoms/208615 41403634 PMC12703477

[97] Wang L Chen X Yan C . Ferroptosis: An emerging therapeutic opportunity for cancer. Genes Dis. (2020) 9:334. doi: 10.1016/J.GENDIS.2020.09.005 35224150 PMC8843872

[98] Guo F Zong S Zhang X Ren Z Shao H Li J . Ferroptosis and metastasis: Molecular checkpoints, microenvironmental dynamics, and therapeutic opportunities. Mol Cancer. (2026) 25:45. doi: 10.1186/S12943-025-02544-Y 41535945 PMC12947519

[99] Yu S Zhang H Zhang S Zhong M Fan H . Ferrite nanoparticles-based reactive oxygen species-mediated cancer therapy. Front Chem. (2021) 9:651053. doi: 10.3389/FCHEM.2021.651053 33987168 PMC8110829

[100] Shestovskaya MV Luss AL Bezborodova OA Makarov VV Keskinov AA . Iron oxide nanoparticles in cancer treatment: Cell responses and the potency to improve radiosensitivity. Pharmaceutics. (2023) 15:2406. doi: 10.3390/PHARMACEUTICS15102406 37896166 PMC10610190

[101] Kontoghiorghes GJ Kontoghiorghe CN . Efficacy and safety of iron-chelation therapy with deferoxamine, deferiprone, and deferasirox for the treatment of iron-loaded patients with non-transfusion-dependent thalassemia syndromes. Drug Des Devel Ther. (2016) 10:465–81. doi: 10.2147/DDDT.S79458 26893541 PMC4745840

[102] You H Wang D Wei L Chen J Li H Liu Y . Deferoxamine inhibits acute lymphoblastic leukemia progression through repression of ROS/HIF-1α, Wnt/β-catenin, and p38MAPK/ERK pathways. J Oncol. (2022) 2022:1–10. doi: 10.1155/2022/8281267 35237325 PMC8885176

[103] Obeagu EI Ngwoke AO Malunga G . Iron chelators in breast cancer therapy: Mechanisms and clinical applications – a narrative review. Ann Med Surg. (2025) 87:3556–65. doi: 10.1097/MS9.0000000000003296 40486569 PMC12140700

[104] Vidanapathirana G Islam MS Gamage S Lam AK Gopalan V . The role of iron chelation therapy in colorectal cancer: A systematic review on its mechanisms and therapeutic potential. Cancer Med. (2025) 14(13):e71019. doi: 10.1002/cam4.71019 40607631 PMC12224056

[105] Lu Z Xiao B Chen W Tang T Zhuo Q Chen X . The potential of ferroptosis combined with radiotherapy in cancer treatment. Front Oncol. (2023) 13. doi: 10.3389/fonc.2023.1085581 37007068 PMC10064444

[106] Lee J Roh J-L . Dihydroorotate dehydrogenase in mitochondrial ferroptosis and cancer therapy. Cells. (2025) 14:1889. doi: 10.3390/cells14231889 41369379 PMC12691135

[107] Chen X Chen S Yu D Zhao W . Inducing ferroptosis: Sensitization strategy for radiotherapy and its application. Antioxidants. (2026) 15:237. doi: 10.3390/antiox15020237 41750617 PMC12938210

[108] Azizi R Ahmed HH Kareem RA Waam WMT Alwan M Jawad MJ . SLC7A11 inhibitors represent a promising therapeutic target by facilitating the induction of ferroptosis in breast cancer. Int J Mol Cell Med. (2025) 14:496–516. 40123584 10.22088/IJMCM.BUMS.14.1.496 PMC11927159

[109] Deng J Zhou M Liao T Kuang W Xia H Yin Z . Targeting cancer cell ferroptosis to reverse immune checkpoint inhibitor therapy resistance. Front Cell Dev Biol. (2022) 10. doi: 10.3389/fcell.2022.818453 35399527 PMC8988234

[110] Li X Li Y Tuerxun H Zhao Y Liu X Zhao Y . Firing up “cold” tumors: Ferroptosis causes immune activation by improving T cell infiltration. Biomedicine Pharmacotherapy. (2024) 179:117298. doi: 10.1016/j.biopha.2024.117298 39151313

[111] Shi L Liu Y Li M Luo Z . Emerging roles of ferroptosis in the tumor immune landscape: From danger signals to anti-tumor immunity. FEBS J. (2022) 289:3655–65. doi: 10.1111/FEBS.16034 34042258

[112] Zhai X Lin Y Zhu L Wang Y Zhang J Liu J . Ferroptosis in cancer immunity and immunotherapy: Multifaceted interplay and clinical implications. Cytokine Growth Factor Rev. (2024) 75:101–9. doi: 10.1016/J.CYTOGFR.2023.08.004 37658030

[113] Tungsukruthai S Petpiroon N Chanvorachote P . Molecular mechanisms of breast cancer metastasis and potential anti-metastatic compounds. Anticancer Res. (2018) 38:2607–18. doi: 10.21873/ANTICANRES.12502 29715080

[114] Anders CK Carey LA . Biology, metastatic patterns, and treatment of patients with triple-negative breast cancer. Clin Breast Cancer. (2009) 9:S73. doi: 10.3816/CBC.2009.S.008 19596646 PMC2919761

[115] Weigelt B Peterse JL Van’t Veer LJ . Breast cancer metastasis: Markers and models. Nat Rev Cancer. (2005) 5:591–602. doi: 10.1038/NRC1670 16056258

[116] Wang Y Zhou BP . Epithelial-mesenchymal transition in breast cancer progression and metastasis. Chin J Cancer. (2011) 30:603–11. doi: 10.5732/CJC.011.10226 21880181 PMC3702729

[117] Nathanson SD Detmar M Padera TP Yates LR Welch DR Beadnell TC . Mechanisms of breast cancer metastasis. Clin Exp Metastasis. (2021) 39:117. doi: 10.1007/S10585-021-10090-2 33950409 PMC8568733

[118] Van Zijl F Krupitza G Mikulits W . Initial steps of metastasis: Cell invasion and endothelial transmigration. Mutat Res Rev Mutat Res. (2011) 728:23–34. doi: 10.1016/j.mrrev.2011.05.002 21605699 PMC4028085

[119] Valastyan S Weinberg RA . Tumor metastasis: Molecular insights and evolving paradigms. Cell. (2011) 147:275–92. doi: 10.1016/j.cell.2011.09.024 22000009 PMC3261217

[120] Azevedo AS Follain G Patthabhiraman S Harlepp S Goetz JG . Metastasis of circulating tumor cells: Favorable soil or suitable biomechanics, or both? Cell Adh Migr. (2015) 9:345–56. doi: 10.1080/19336918.2015.1059563 26312653 PMC4955369

[121] Jenning S Pham T Ireland SK Ruoslahti E Biliran H . Bit1 in anoikis resistance and tumor metastasis. Cancer Lett. (2013) 333:147. doi: 10.1016/J.CANLET.2013.01.043 23376255 PMC3651913

[122] Castaneda M den Hollander P Kuburich NA Rosen JM Mani SA . Mechanisms of cancer metastasis. Semin Cancer Biol. (2022) 87:17–31. doi: 10.1016/J.SEMCANCER.2022.10.006 36354098

[123] El-Tanani M Rabbani SA Babiker R Rangraze I Kapre S Palakurthi SS . Unraveling the tumor microenvironment: Insights into cancer metastasis and therapeutic strategies. Cancer Lett. (2024) 591:216894. doi: 10.1016/J.CANLET.2024.216894 38626856

[124] Li Y Liu F Cai Q Deng L Ouyang Q Zhang XHF . Invasion and metastasis in cancer: Molecular insights and therapeutic targets. Signal Transduct Target Ther. (2025) 10:57. doi: 10.1038/S41392-025-02148-4 39979279 PMC11842613

[125] Fouad FA Khali MA Moaz I Elmasry H Gheta N Abdeen A . Prognostic impact of matrix metalloproteinase 2 (MMP-2) and matrix metalloproteinase 9 (MMP-9) in Egyptian breast cancer patients. Int J Immunopathol Pharmacol. (2024) 38:3946320241304911. doi: 10.1177/03946320241304911 39644100 PMC11624540

[126] Chaudhary P Janmeda P Docea AO Yeskaliyeva B Abdull Razis AF Modu B . Oxidative stress, free radicals and antioxidants: Potential crosstalk in the pathophysiology of human diseases. Front Chem. (2023) 11:1158198. doi: 10.3389/FCHEM.2023.1158198 37234200 PMC10206224

[127] Mukherjee A Das B . The role of inflammatory mediators and matrix metalloproteinases (MMPs) in the progression of osteoarthritis. Biomater Biosyst. (2024) 13:100090. doi: 10.1016/J.BBIOSY.2024.100090 38440290 PMC10910010

[128] Park M Kim D Ko S Kim A Mo K Yoon H . Breast cancer metastasis: Mechanisms and therapeutic implications. Int J Mol Sci. (2022) 23:6806. doi: 10.3390/IJMS23126806 35743249 PMC9224686

[129] Yeeravalli R Das A . Molecular mediators of breast cancer metastasis. Hematol Oncol Stem Cell Ther. (2021) 14:275–89. doi: 10.1016/J.HEMONC.2021.02.002 33744312

[130] Simon A Robb K . Cancer: breast. In: Ayers S Baum A McManus C Newman S Wallston K Weinman J West R , editors. Cambridge Handbook of Psychology, Health and Medicine. Cambridge: Cambridge University Press (2007). 577–580. (Accessed September 7, 2026).

[131] Carvalho E Canberk S Schmitt F Vale N . Molecular subtypes and mechanisms of breast cancer: Precision medicine approaches for targeted therapies. Cancers. (2025) 17:1102. doi: 10.3390/CANCERS17071102 40227634 PMC11987866

[132] Arpita J Priyanka G Ranjana S . A study of molecular subtypes of carcinoma breast by immunohistochemistry at tertiary care center, Jaipur. Asian Pacific J Cancer Biol. (2022) 7:219–23. doi: 10.31557/APJCB.2022.7.3.219-223

[133] Miah S Bagu E Goel R Ogunbolude Y Dai C Ward A . Estrogen receptor signaling regulates the expression of the breast tumor kinase in breast cancer cells. BMC Cancer. (2019) 19(1):78. doi: 10.1186/S12885-018-5186-8 30651078 PMC6335685

[134] Höller A Nguyen-Sträuli BD Frauchiger-Heuer H Ring A . Diagnostic and prognostic biomarkers of luminal breast cancer: Where are we now? Breast Cancer : Targets Ther. (2023) 15:525. doi: 10.2147/BCTT.S340741 37533589 PMC10392911

[135] Orrantia-Borunda E Anchondo-Nuñez P Acuña-Aguilar LE Gómez-Valles FO Ramírez-Valdespino CA . Subtypes of breast cancer. In: Mayrovitz HN , editor. Breast cancer. Brisbane (AU): Exon Publications. doi: 10.36255/exon-publications-breast-cancer (Accessed September 7, 2026). 36122153

[136] Cancello G Maisonneuve P Rotmensz N Viale G Mastropasqua MG Pruneri G . Progesterone receptor loss identifies luminal B breast cancer subgroups at higher risk of relapse. Ann Oncol. (2013) 24:661–8. doi: 10.1093/ANNONC/MDS430 23022996

[137] Geyer FC Pareja F Weigelt B Rakha E Ellis IO Schnitt SJ . The spectrum of triple-negative breast disease: High- and low-grade lesions. Am J Pathol. (2017) 187:2139–51. doi: 10.1016/J.AJPATH.2017.03.016 28736315 PMC5809519

[138] Yin L Duan JJ Bian XW Yu SC . Triple-negative breast cancer molecular subtyping and treatment progress. Breast Cancer Res. (2020) 22:1–13. doi: 10.1186/S13058-020-01296-5 32517735 PMC7285581

[139] Almansour NM . Triple-negative breast cancer: A brief review about epidemiology, risk factors, signaling pathways, treatment and role of artificial intelligence. Front Mol Biosci. (2022) 9:836417. doi: 10.3389/FMOLB.2022.836417 35145999 PMC8824427

[140] Pegram M Jackisch C Johnston SRD . Estrogen/HER2 receptor crosstalk in breast cancer: Combination therapies to improve outcomes for patients with hormone receptor-positive/HER2-positive breast cancer. NPJ Breast Cancer. (2023) 9:45. doi: 10.1038/S41523-023-00533-2 37258523 PMC10232442

[141] Godoy-Ortiz A Sanchez-Muñoz A Parrado MRC Álvarez M Ribelles N Dominguez AR . Deciphering HER2 breast cancer disease: Biological and clinical implications. Front Oncol. (2019) 9:1124. doi: 10.3389/FONC.2019.01124 31737566 PMC6828840

[142] Turova P Kushnarev V Baranov O Butusova A Menshikova S Yong ST . The breast cancer classifier refines molecular breast cancer classification to delineate the HER2-low subtype. NPJ Breast Cancer. (2025) 11:1–16. doi: 10.1038/s41523-025-00723-0 39979291 PMC11842814

[143] Kim S Moon BI Lim W Park S Cho MS Sung SH . Feasibility of classification of triple negative breast cancer by immunohistochemical surrogate markers. Clin Breast Cancer. (2018) 18:e1123–32. doi: 10.1016/J.CLBC.2018.03.012 29754847

[144] Erickson LA Mete O Juhlin CC Perren A Gill AJ . Overview of the 2022 WHO classification of parathyroid tumors. Endocr Pathol. (2022) 33(1):64–89. doi: 10.1007/s12022-022-09709-1 35175514

[145] Amin MB Greene FL Edge SB Compton CC Gershenwald JE Brookland RK . The eighth edition AJCC cancer staging manual: Continuing to build a bridge from a population‐based to a more “personalized” approach to cancer staging. CA Cancer J Clin. (2017) 67:93–9. doi: 10.3322/CAAC.21388 28094848

[146] Plichta JK Campbell BM Mittendorf EA Hwang ES . Anatomy and breast cancer staging: Is it still relevant? Surg Oncol Clin N Am. (2018) 27:51–67. doi: 10.1016/J.SOC.2017.07.010 29132565

[147] Zhu H Doğan BE . American Joint Committee on Cancer’s staging system for breast cancer, eighth edition: Summary for clinicians. Eur J Breast Health. (2021) 17:234. doi: 10.4274/EJBH.GALENOS.2021.2021-4-3 34263150 PMC8246053

[148] Canadian Cancer Society . Stages of breast cancer (2023). Available online at: https://cancer.ca/en/cancer-information/cancer-types/breast/staging (Accessed September 7, 2025).

[149] National Breast Cancer Foundation (NBCF) Team . Stage 4 (IV) Breast Cancer: Survival Rates, Treatment & Prognosis (2025). Available online at: https://www.nationalbreastcancer.org/breast-cancer-stage-4/ (Accessed September 7, 2025).

[150] Khatib OMN Modjtabai A . Guidelines for the early detection and screening of breast cancer editors. In: Khatib OMN Modjtabai A , editors.Guidelines for the early detection and screening of breast cancer editors. World Health Organization, Cairo 11371, Egypt (2006). p. 1–57.

[151] Fuller MS Lee CI Elmore JG . Breast cancer screening: An evidence-based update. Med Clinics North America. (2015) 99:451–68. doi: 10.1016/j.mcna.2015.01.002 25841594 PMC5064844

[152] Henderson JA Duffee D Ferguson T . Breast Examination Techniques (2023). Available online at: https://www.ncbi.nlm.nih.gov/books/NBK459179/ (Accessed September 07, 2025).

[153] Ding Y Sun C Zhou Q Cheng C Yan C Wang B . Use of palpation imaging in diagnosis of breast diseases: A way to improve the detection rate. Med Sci Monit. (2020) 26:e927553-1. doi: 10.12659/MSM.927553 33247894 PMC7709466

[154] Ohaeri B Aderigbigbe M . Knowledge and use of breast self-examination and mammogram among women of reproductive age in Oyo State Secretariat, Ibadan, Oyo State, Nigeria. Eur J Midwifery. (2019) 3:7. doi: 10.18332/ejm/105858 33537586 PMC7839162

[155] Huang N Chen L He J Nguyen QD . The efficacy of clinical breast exams and breast self-exams in detecting Malignancy or positive ultrasound findings. Cureus. (2022) 14:e22464. doi: 10.7759/CUREUS.22464 35371742 PMC8942605

[156] Iranmakani S Mortezazadeh T Sajadian F Ghaziani MF Ghafari A Khezerloo D . A review of various modalities in breast imaging: Technical aspects and clinical outcomes. Egypt J Radiol Nucl Med. (2020) 51:1–22. doi: 10.1186/s43055-020-00175-5

[157] Fico N Di Grezia G Cuccurullo V Salvia AAH Iacomino A Sciarra A . Breast imaging physics in mammography (Part I). Diagnostics. (2023) 13:3227. doi: 10.3390/DIAGNOSTICS13203227 37892053 PMC10606465

[158] Nicholson WK Silverstein M Wong JB Barry MJ Chelmow D Coker TR . Screening for breast cancer: US Preventive Services Task Force recommendation statement. JAMA. (2024) 331:1918–30. doi: 10.1001/JAMA.2024.5534 38687503

[159] Tomlinson-Hansen SE Budh DP Sapra A . Breast Cancer Screening in the Average-Risk Patient (2024). Available online at: https://www.ncbi.nlm.nih.gov/books/NBK556050/ (Accessed September 07, 2025). 32310510

[160] Ramani SK Rastogi A Mahajan A Nair N Shet T Thakur MH . Imaging of the treated breast post breast conservation surgery/oncoplasty: Pictorial review. World J Radiol. (2017) 9:321. doi: 10.4329/WJR.V9.I8.321 28932361 PMC5583527

[161] Hasan S Abel S Simpson-Camp LS Witten M Aguilera L Teng L . Short-term follow-up mammography in breast conservation therapy likely leads to unnecessary downstream workup: A longitudinal study. Int J Radiat Oncol Biol Phys. (2018) 102:1489–95. doi: 10.1016/J.IJROBP.2017.09.031 29102277

[162] Hooley RJ Scoutt LM Philpotts LE . Breast ultrasonography: State of the art. Radiology. (2013) 268:642–59. doi: 10.1148/RADIOL.13121606 23970509

[163] Ontario HQ . Ultrasound as an adjunct to mammography for breast cancer screening: A health technology assessment (2016). Available online at: https://pmc.ncbi.nlm.nih.gov/articles/PMC4947971/ (Accessed September 07, 2025). PMC4947971 27468326

[164] Gao LY Gu Y Xu W Tian JW Yin LX Ran HT . Can combined screening of ultrasound and elastography improve breast cancer identification compared with MRI in women with dense breasts-a multicenter prospective study. J Cancer. (2020) 11:3903–9. doi: 10.7150/JCA.43326 32328194 PMC7171498

[165] Radhakrishna S Agarwal S Parikh PM Kaur K Panwar S Sharma S . Role of magnetic resonance imaging in breast cancer management. South Asian J Cancer. (2018) 7:69. doi: 10.4103/SAJC.SAJC_104_18 29721466 PMC5909298

[166] Mann RM Cho N Moy L . Breast MRI: state of the art. Radiology. (2019) 292:520–36. doi: 10.1148/RADIOL.2019182947 31361209

[167] Washington I Palm RF White J Rosenberg SA Ataya D . The role of MRI in breast cancer and breast conservation therapy. Cancers 2024 Vol 16 Page 2122. (2024) 16:2122. doi: 10.3390/CANCERS16112122 38893241 PMC11171236

[168] Zhang Y Qian Y Zhang Y Qian Y . Application of magnetic resonance imaging in breast cancer patients. (2025). doi: 10.5772/INTECHOPEN.1008509

[169] Sánchez-Bayuela DÁ Castellano R Angulo A Giovanetti González PM Cruz Hernández R Ruiz LM . Dielectric characterization of breast biopsied tissues as pre-pathological aid in early cancer detection: A blinded feasibility study. Diagnostics. (2023) 13:3015. doi: 10.3390/DIAGNOSTICS13183015 37761382 PMC10527865

[170] Khan AQ Touseeq M Rehman S Tahir M Ashfaq M Jaffar E . Advances in breast cancer diagnosis: A comprehensive review of imaging, biosensors, and emerging wearable technologies. Front Oncol. (2025) 15:1587517. doi: 10.3389/FONC.2025.1587517 40606998 PMC12213879

[171] Cheng Y Fu M . Dielectric properties for non‐invasive detection of normal, benign, and Malignant breast tissues using microwave theories. Thorac Cancer. (2018) 9:459–65. doi: 10.1111/1759-7714.12605 29465782 PMC5879051

[172] Janjic A Cayoren M Akduman I Yilmaz T Onemli E Bugdayci O . SAFE: A novel microwave imaging system design for breast cancer screening and early detection—Clinical evaluation. Diagnostics. (2021) 11:533. doi: 10.3390/diagnostics11030533 33809770 PMC8002280

[173] Rana SP Dey M Loretoni R Duranti M Sani L Vispa A . Radial basis function for breast lesion detection from MammoWave clinical data. Diagnostics. (2021) 11:1930. doi: 10.3390/DIAGNOSTICS11101930 34679628 PMC8534354

[174] Fernández-Aranzamendi EG Castillo-Araníbar PR San Román Castillo EG Oller BS Ventura-Zaa L Eguiluz-Rodriguez G . Dielectric characterization of ex-vivo breast tissues: Differentiation of tumor types through permittivity measurements. Cancers (Basel). (2024) 16:793. doi: 10.3390/cancers16040793 38398184 PMC10886458

[175] Chen Q Rao X Cao D Huang Q Xia X Wang S . Real-time differentiation between benign and Malignant breast tumors and other tissues using dielectric properties. Med Sci Monit. (2025) 31:e947531. doi: 10.12659/MSM.947531 40526582 PMC12181975

[176] Chen S Lu Z Huang Q Zhao G Sun Z Zhou J . An optimized sliding rail-assisted micrometer system for sensing volume measurement of open-ended coaxial probes in breast cancer dielectric property analysis. Front Bioeng Biotechnol. (2025) 13. doi: 10.3389/fbioe.2025.1575142 40948978 PMC12426283

[177] Arravalli T Chadaga K Muralikrishna H Sampathila N Cenitta D Chadaga R . Detection of breast cancer using machine learning and explainable artificial intelligence. Sci Rep. (2025) 15:26931. doi: 10.1038/s41598-025-12644-w 40707590 PMC12290098

[178] Wang Y Wei X Wu M . Diagnostic efficacy of multimodal imaging in non-mass-like breast lesions and correlation with pathological findings. Front Oncol. (2025) 15. doi: 10.3389/fonc.2025.1719528 41458587 PMC12740895

[179] Alotaibi SA Gareeballah A Sami Ur Rahman J Faqihi WA Ali S Elbashir M . Evaluation of the diagnostic accuracy of mammography and ultrasonography for breast cancer diagnosis using histopathology as the reference standard: A cross-sectional study. Ann Med Surg. (2026) 88:159–66. doi: 10.1097/MS9.0000000000004369 41497117 PMC12768151

[180] Schneble EJ Graham LJ Shupe MP Flynt FL Banks KP Kirkpatrick AD . Future directions for the early detection of recurrent breast cancer. J Cancer. (2014) 5:291–300. doi: 10.7150/JCA.8017 24790657 PMC3982042

[181] Ahuja S Sureka N Zaheer S . Unraveling the intricacies of cancer‐associated fibroblasts: A comprehensive review on metabolic reprogramming and tumor microenvironment crosstalk. APMIS. (2024) 132:906–27. doi: 10.1111/apm.13447 38873945

[182] Yang M Mu Y Yu X Gao D Zhang W Li Y . Survival strategies: How tumor hypoxia microenvironment orchestrates angiogenesis. Biomedicine Pharmacotherapy. (2024) 176:116783. doi: 10.1016/j.biopha.2024.116783 38796970

[183] Greenwood HE Witney TH . Latest advances in imaging oxidative stress in cancer. J Nucl Med. (2021) 62:1506–10. doi: 10.2967/jnumed.120.256974 34353871 PMC7611938

[184] Sun T Zhao H Hu L Shao X Lu Z Wang Y . Enhanced optical imaging and fluorescent labeling for visualizing drug molecules within living organisms. Acta Pharm Sin B. (2024) 14:2428–46. doi: 10.1016/j.apsb.2024.01.018 38828150 PMC11143489

[185] Berezin MY Guo K Akers W Northdurft RE Culver JP Teng B . Near-infrared fluorescence lifetime pH-sensitive probes. Biophys J. (2011) 100:2063–72. doi: 10.1016/j.bpj.2011.02.050 21504743 PMC3077686

[186] Rizzo R Onesto V Morello G Iuele H Scalera F Forciniti S . pH-sensing hybrid hydrogels for non-invasive metabolism monitoring in tumor spheroids. Mater Today Bio. (2023) 20:100655. doi: 10.1016/j.mtbio.2023.100655 37234366 PMC10205545

[187] Wang ZJ Kumar R Banerjee S Hsu C . Blood oxygen level‐dependent (BOLD) MRI of diabetic nephropathy: Preliminary experience. J Magn Reson Imaging. (2011) 33:655–60. doi: 10.1002/jmri.22501 21563249 PMC3573698

[188] Neugarten J Golestaneh L . Blood oxygenation level-dependent MRI for assessment of renal oxygenation. Int J Nephrol Renovasc Dis. (2014) 7:421–35. doi: 10.2147/IJNRD.S42924 25473304 PMC4247132

[189] Weintraub A Whyte J . Blood oxygen level dependent (BOLD). In: Encyclopedia of Clinical Neuropsychology. Springer International Publishing, Cham (2018). p. 599–601. doi: 10.1007/978-3-319-57111-9_10

[190] Bouleftour W Rowinski E Louati S Sotton S Wozny A-S Moreno-Acosta P . A review of the role of hypoxia in radioresistance in cancer therapy. Med Sci Monit. (2021) 27:e934116. doi: 10.12659/MSM.934116 34728593 PMC8573967

[191] Hill RM Rocha S Parsons JL . Overcoming the impact of hypoxia in driving radiotherapy resistance in head and neck squamous cell carcinoma. Cancers (Basel). (2022) 14:4130. doi: 10.3390/cancers14174130 36077667 PMC9454974

[192] Mensah EA Faiyaz A Schifitto G Uddin MN . Chemical exchange saturation transfer imaging in neuroinflammation: Methods, challenges, and recommendations. Int J Mol Sci. (2025) 26:11059. doi: 10.3390/ijms262211059 41303541 PMC12652846

[193] Dubey S . Chemical exchange saturation transfer magnetic resonance imaging (CEST MRI) in lung cancer: A narrative review of translational challenges and clinical potential. Cureus. (2026) 18(1): e102263. doi: 10.7759/cureus.102263 41743005 PMC12931733

[194] Anemone A Consolino L Conti L Irrera P Hsu MY Villano D . Tumour acidosis evaluated *in vivo* by MRI-CEST pH imaging reveals breast cancer metastatic potential. Br J Cancer. (2021) 124:207–16. doi: 10.1038/s41416-020-01173-0 33257841 PMC7782702

[195] Mu M Zhang M Liu J Ren K Liu H Yip L . Real-time magnetic resonance visualization of tumor acidosis as a precognition indicator of therapeutic efficacy. Bioact Mater. (2025) 52:63–72. doi: 10.1016/j.bioactmat.2025.05.033 40530412 PMC12172170

[196] Malik MMUD Alqahtani MM Hadadi I Kanbayti I Alawaji Z Aloufi BA . Molecular imaging biomarkers for early cancer detection: A systematic review of emerging technologies and clinical applications. Diagnostics. (2024) 14:2459. doi: 10.3390/diagnostics14212459 39518426 PMC11545511

[197] Stefanes NM Cunha-Silva ME de Oliveira Silva L Walter LO Santos-Silva MC Gartia MR . Circulating biomarkers for diagnosis and response to therapies in cancer patients. (2025) 391:1–41. doi: 10.1016/bs.ircmb.2024.08.007 PMC11969414 39939074

[198] Grimes DR Warren DR Warren S . Hypoxia imaging and radiotherapy: bridging the resolution gap. Br J Radiol. (2017) 90:20160939. doi: 10.1259/bjr.20160939 28540739 PMC5603947

[199] Bai Y Osmundson EC Donahue MJ De Vis JB . Magnetic resonance imaging to detect tumor hypoxia in brain Malignant disease: a systematic review of validation studies. Clin Transl Radiat Oncol. (2025) 52:100940. doi: 10.1016/j.ctro.2025.100940 40093743 PMC11908384

[200] Junaid SB Imam AA Balogun AO De Silva LC Surakat YA Kumar G . Recent advancements in emerging technologies for healthcare management systems: a survey. Healthcare. (2022) 10:1940. doi: 10.3390/healthcare10101940 36292387 PMC9601636

[201] Costa V Custodio MG Gefen E Fregni F . The relevance of the real-world evidence in research, clinical, and regulatory decision making. Front Public Health. (2025) 13. doi: 10.3389/fpubh.2025.1512429 40041193 PMC11878099

[202] Fahim YA Hasani IW Kabba S Ragab WM . Artificial intelligence in healthcare and medicine: clinical applications, therapeutic advances, and future perspectives. Eur J Med Res. (2025) 30:848. doi: 10.1186/s40001-025-03196-w 40988064 PMC12455834

[203] Martemucci G Costagliola C Mariano M D’andrea L Napolitano P D’Alessandro AG . Free radical properties, source and targets, antioxidant consumption and health. Oxygen. (2022) 2:48–78. doi: 10.3390/OXYGEN2020006 30654563

[204] Dong S Lyu X Yuan S Wang S Li W Chen Z . Oxidative stress: a critical hint in ionizing radiation induced pyroptosis. Radiat Med Prot. (2020) 1:179–85. doi: 10.1016/J.RADMP.2020.10.001 38826717

[205] Vicidomini C Palumbo R Moccia M Roviello GN . Oxidative processes and xenobiotic metabolism in plants: mechanisms of defense and potential therapeutic implications. J Xenobiot. (2024) 14:1541. doi: 10.3390/JOX14040084 39449425 PMC11503355

[206] Juan CA de la Lastra JMP Plou FJ Pérez-Lebeña E . The chemistry of reactive oxygen species (ROS) revisited: outlining their role in biological macromolecules (DNA, lipids and proteins) and induced pathologies. Int J Mol Sci. (2021) 22:4642. doi: 10.3390/IJMS22094642 33924958 PMC8125527

[207] Kowalczyk P Sulejczak D Kleczkowska P Bukowska-Ośko I Kucia M Popiel M . Mitochondrial oxidative stress—a causative factor and therapeutic target in many diseases. Int J Mol Sci. (2021) 22:13384. doi: 10.3390/IJMS222413384 34948180 PMC8707347

[208] Klaunig JE . Oxidative stress and cancer. Curr Pharm Des. (2019) 24:4771–8. doi: 10.2174/1381612825666190215121712 30767733

[209] Allegra A Tonacci A Giordano L Musolino C Gangemi S . Targeting redox regulation as a therapeutic opportunity against acute leukemia: pro-oxidant strategy or antioxidant approach? Antioxidants. (2022) 11:1696. doi: 10.3390/ANTIOX11091696 36139768 PMC9495346

[210] Klaunig JE Wang Z . Oxidative stress in carcinogenesis. Curr Opin Toxicol. (2018) 7:116–21. doi: 10.1016/J.COTOX.2017.11.014 38826717

[211] Davalli P Marverti G Lauriola A D’Arca D . Targeting oxidatively induced DNA damage response in cancer: opportunities for novel cancer therapies. Oxid Med Cell Longev. (2018) 2018:2389523. doi: 10.1155/2018/2389523 29770165 PMC5892224

[212] Jelic MD Mandic AD Maricic SM Srdjenovic BU . Oxidative stress and its role in cancer. J Cancer Res Ther. (2021) 17:22–8. doi: 10.4103/JCRT.JCRT_862_16 33723127

[213] Janion K Strzelczyk JK Walkiewicz KW Biernacki K Copija A Szczepańska E . Evaluation of malondialdehyde level, total oxidant/antioxidant status and oxidative stress index in colorectal cancer patients. Metabolites. (2022) 12:1118. doi: 10.3390/METABO12111118 36422258 PMC9695970

[214] Pizzino G Irrera N Cucinotta M Pallio G Mannino F Arcoraci V . Oxidative stress: harms and benefits for human health. Oxid Med Cell Longev. (2017) 2017:8416763. doi: 10.1155/2017/8416763 28819546 PMC5551541

[215] Sharifi-Rad M Anil Kumar NV Zucca P Varoni EM Dini L Panzarini E . Lifestyle, oxidative stress, and antioxidants: back and forth in the pathophysiology of chronic diseases. Front Physiol. (2020) 11:694. doi: 10.3389/FPHYS.2020.00694 32714204 PMC7347016

[216] Wang M Xiao Y Miao J Zhang X Liu M Zhu L . Oxidative stress and inflammation: drivers of tumorigenesis and therapeutic opportunities. Antioxidants. (2025) 14:735. doi: 10.3390/ANTIOX14060735 40563367 PMC12189506

[217] Arfin S Jha NK Jha SK Kesari KK Ruokolainen J Roychoudhury S . Oxidative stress in cancer cell metabolism. Antioxidants. (2021) 10:642. doi: 10.3390/ANTIOX10050642 33922139 PMC8143540

[218] Iqbal MJ Kabeer A Abbas Z Siddiqui HA Calina D Sharifi-Rad J . Interplay of oxidative stress, cellular communication and signaling pathways in cancer. Cell Commun Signal. (2024) 22(1):7. doi: 10.1186/s12964-023-01398-5 38167159 PMC10763046

[219] De Almeida AJPO De Oliveira JCPL Da Silva Pontes LV De Souza Júnior JF Gonçalves TAF Dantas SH . ROS: basic concepts, sources, cellular signaling, and its implications in aging pathways. Oxid Med Cell Longev. (2022) 2022:1225578. doi: 10.1155/2022/1225578 36312897 PMC9605829

[220] Ansari WA Srivastava K Nasibullah M Khan MF . Reactive oxygen species (ROS): sources, generation, disease pathophysiology, and antioxidants. Discover Chem. (2025) 2:1–33. doi: 10.1007/S44371-025-00275-Z 30311153

[221] Reuter S Gupta SC Chaturvedi MM Aggarwal BB . Oxidative stress, inflammation, and cancer: how are they linked? Free Radic Biol Med. (2010) 49:1603–16. doi: 10.1016/J.FREERADBIOMED.2010.09.006 20840865 PMC2990475

[222] Chavda V Chaurasia B Garg K Deora H Umana GE Palmisciano P . Molecular mechanisms of oxidative stress in stroke and cancer. Brain Disord. (2022) 5:100029. doi: 10.1016/J.DSCB.2021.100029 38826717

[223] Hong Y Boiti A Vallone D Foulkes NS . Reactive oxygen species signaling and oxidative stress: transcriptional regulation and evolution. Antioxidants. (2024) 13:312. doi: 10.3390/ANTIOX13030312 38539845 PMC10967436

[224] Kim EK Choi EJ . Pathological roles of MAPK signaling pathways in human diseases. Biochim Biophys Acta (BBA) - Mol Basis Dis. (2010) 1802:396–405. doi: 10.1016/J.BBADIS.2009.12.009 20079433

[225] Ng GYQ Loh Z-L Fann DY Mallilankaraman K Arumugam TV Hande MP . Role of mitogen-activated protein (MAP) kinase pathways in metabolic diseases. Genome Integr. (2024) 15:e20230003. doi: 10.14293/GENINT.14.1.004 38770527 PMC11102075

[226] Cook SJ Stuart K Gilley R Sale MJ . Control of cell death and mitochondrial fission by ERK1/2 MAP kinase signalling. FEBS J. (2017) 284:4177–95. doi: 10.1111/FEBS.14122 28548464 PMC6193418

[227] Kong T Liu M Ji B Bai B Cheng B Wang C . Role of the extracellular signal-regulated kinase 1/2 signaling pathway in ischemia-reperfusion injury. Front Physiol. (2019) 10:469697. doi: 10.3389/FPHYS.2019.01038 31474876 PMC6702336

[228] Guo Y-J Pan W-W Liu S-B Shen Z-F Xu Y Hu L-L . ERK/MAPK signalling pathway and tumorigenesis. Exp Ther Med. (2020) 19:1997. doi: 10.3892/ETM.2020.8454 32104259 PMC7027163

[229] Marengo B Nitti M Furfaro AL Colla R Ciucis CD Marinari UM . Redox homeostasis and cellular antioxidant systems: crucial players in cancer growth and therapy. Oxid Med Cell Longev. (2016) 2016:6235641. doi: 10.1155/2016/6235641 27418953 PMC4932173

[230] Ngo V Duennwald ML . Nrf2 and oxidative stress: a general overview of mechanisms and implications in human disease. Antioxidants. (2022) 11:2345. doi: 10.3390/ANTIOX11122345 36552553 PMC9774434

[231] Chen F Xiao M Hu S Wang M . Keap1-Nrf2 pathway: a key mechanism in the occurrence and development of cancer. Front Oncol. (2024) 14:1381467. doi: 10.3389/FONC.2024.1381467 38634043 PMC11021590

[232] Yu C Xiao JH . The Keap1-Nrf2 system: a mediator between oxidative stress and aging. Oxid Med Cell Longev. (2021) 2021:6635460. doi: 10.1155/2021/6635460 34012501 PMC8106771

[233] Hammad M Raftari M Cesário R Salma R Godoy P Emami SN . Roles of oxidative stress and Nrf2 signaling in pathogenic and non-pathogenic cells: a possible general mechanism of resistance to therapy. Antioxidants. (2023) 12:1371. doi: 10.3390/ANTIOX12071371 37507911 PMC10376708

[234] Jenkins T Gouge J . Nrf2 in cancer, detoxifying enzymes and cell death programs. Antioxidants. (2021) 10:1030. doi: 10.3390/ANTIOX10071030 34202320 PMC8300779

[235] Schmidlin CJ Shakya A Dodson M Chapman E Zhang DD . The intricacies of NRF2 regulation in cancer. Semin Cancer Biol. (2021) 76:110. doi: 10.1016/J.SEMCANCER.2021.05.016 34020028 PMC8599504

[236] Taguchi K Yamamoto M . The KEAP1–NRF2 system in cancer. Front Oncol. (2017) 7:85. doi: 10.3389/FONC.2017.00085 28523248 PMC5415577

[237] Marks JR . Refining the role of BRCA1 in combating oxidative stress. Breast Cancer Res. (2013) 15:320. doi: 10.1186/BCR3583 24314328 PMC3978477

[238] Yi YW Kang HJ Bae I . BRCA1 and oxidative stress. Cancers (Basel). (2014) 6:771. doi: 10.3390/CANCERS6020771 24704793 PMC4074803

[239] Rojo de la Vega M Chapman E Zhang DD . NRF2 and the hallmarks of cancer. Cancer Cell. (2018) 34:21–43. doi: 10.1016/J.CCELL.2018.03.022 29731393 PMC6039250

[240] Gorrini C Gang BP Bassi C Wakeham A Baniasadi SP Hao Z . Estrogen controls the survival of BRCA1-deficient cells via a PI3K-NRF2-regulated pathway. Proc Natl Acad Sci USA. (2014) 111:4472–7. doi: 10.1073/PNAS.1324136111 24567396 PMC3970526

[241] Su LJ Zhang JH Gomez H Murugan R Hong X Xu D . Reactive oxygen species-induced lipid peroxidation in apoptosis, autophagy, and ferroptosis. Oxid Med Cell Longev. (2019) 2019:5080843. doi: 10.1155/2019/5080843 31737171 PMC6815535

[242] Chen S Li Q Shi H Li F Duan Y Guo Q . New insights into the role of mitochondrial dynamics in oxidative stress-induced diseases. Biomedicine Pharmacotherapy. (2024) 178:117084. doi: 10.1016/J.BIOPHA.2024.117084 39088967

[243] Chandimali N Bak SG Park EH Lim HJ Won YS Kim EK . Free radicals and their impact on health and antioxidant defenses: a review. Cell Death Discov. (2025) 11:1–17. doi: 10.1038/s41420-024-02278-8 39856066 PMC11760946

[244] Zheng Y Sun J Luo Z Li Y Huang Y . Emerging mechanisms of lipid peroxidation in regulated cell death and its physiological implications. Cell Death Dis. (2024) 15:859. doi: 10.1038/S41419-024-07244-X 39587094 PMC11589755

[245] Al-Aubaidy HA Jelinek HF . 8-Hydroxy-2-deoxy-guanosine identifies oxidative DNA damage in a rural prediabetes cohort. Redox Rep. (2010) 15:155–60. doi: 10.1179/174329210X12650506623681 20663291 PMC7067313

[246] Korkmaz KS Butuner BD Roggenbuck D . Detection of 8-OHdG as a diagnostic biomarker. J Lab Precis Med. (2018) 3:95–. doi: 10.21037/JLPM.2018.11.01

[247] Bronckaers A Gago F Balzarini J Liekens S . The dual role of thymidine phosphorylase in cancer development and chemotherapy. Med Res Rev. (2009) 29:903–53. doi: 10.1002/MED.20159 19434693 PMC7168469

[248] Li W Yue H . Thymidine phosphorylase: a potential new target for treating cardiovascular disease. Trends Cardiovasc Med. (2017) 28:157. doi: 10.1016/J.TCM.2017.10.003 29108898 PMC5856583

[249] Shahzad SA Sarfraz A Yar M Khan ZA Naqvi SAR Naz S . Synthesis, evaluation of thymidine phosphorylase and angiogenic inhibitory potential of ciprofloxacin analogues: Repositioning of ciprofloxacin from antibiotic to future anticancer drugs. Bioorg Chem. (2020) 100:103876. doi: 10.1016/J.BIOORG.2020.103876 32388426

[250] Liu H Ma L Li C Cao B Jiang Y Han L . The molecular mechanism of chronic stress affecting the occurrence and development of breast cancer and potential drug therapy. Transl Oncol. (2022) 15:101281. doi: 10.1016/J.TRANON.2021.101281 34875482 PMC8652015

[251] Haghmorad D Razavi FT Eivazzadeh Y Yazdanpanah E Orooji N . Therapeutic challenges in breast cancer: Navigating the impact of oxidative stress on treatment efficacy and toxicity. Biomedicine Pharmacotherapy. (2025) 190:118364. doi: 10.1016/J.BIOPHA.2025.118364 40683207

[252] Li K Deng Z Lei C Ding X Li J Wang C . The role of oxidative stress in tumorigenesis and progression. Cells. (2024) 13:441. doi: 10.3390/CELLS13050441 38474405 PMC10931308

[253] Tiwari R Mondal Y Bharadwaj K Mahajan M Mondal S Sarkar A . Reactive oxygen species (ROS) and their profound influence on regulating diverse aspects of cancer: A concise review. Drug Dev Res. (2025) 86:e70107. doi: 10.1002/DDR.70107 40439342

[254] Usin SG Daramola OO . Phytochemical analysis and evaluation of ethanol and aqueous extracts of Piliostigma thonningii leaf for *in vitro* antioxidant activities. Nigerian J Biochem Mol Biol. (2022) 37:72–81.

[255] Tumilaar SG Hardianto A Dohi H Kurnia D . A comprehensive review of free radicals, oxidative stress, and antioxidants: Overview, clinical applications, global perspectives, future directions, and mechanisms of antioxidant activity of flavonoid compounds. J Chem. (2024) 2024:5594386. doi: 10.1155/2024/5594386 40201959

[256] Ighodaro OM Akinloye OA . First line defence antioxidants-superoxide dismutase (SOD), catalase (CAT) and glutathione peroxidase (GPX): Their fundamental role in the entire antioxidant defence grid. Alexandria J Med. (2018) 54:287–93. doi: 10.1016/J.AJME.2017.09.001 38826717

[257] Singh YP Patel RN Singh Y Butcher RJ Vishakarma PK Singh RKB . Structure and antioxidant superoxide dismutase activity of copper(II) hydrazone complexes. Polyhedron. (2017) 122:1–15. doi: 10.1016/j.poly.2016.11.013 38826717

[258] Perera NCN Godahewa GI Lee S Kim M-J Hwang JY Kwon MG . Manganese-superoxide dismutase (MnSOD), a role player in seahorse (Hippocampus abdominalis) antioxidant defense system and adaptive immune system. Fish Shellfish Immunol. (2017) 68:435–42. doi: 10.1016/j.fsi.2017.07.049 28743628

[259] Grigoras AG . Catalase immobilization—a review. Biochem Eng J. (2017) 117:1–20. doi: 10.1016/j.bej.2016.10.021 38826717

[260] Cardoso BR Hare DJ Bush AI Roberts BR . Glutathione peroxidase 4: a new player in neurodegeneration? Mol Psychiatry. (2017) 22:328–35. doi: 10.1038/mp.2016.196 27777421

[261] Stone WL Basit H Mohiuddin SS . Biochemistry, antioxidants. In: StatPearls. Treasure Island (Florida): StatPearls Publishing (2023). Available online at: https://www.ncbi.nlm.nih.gov/sites/books/NBK541064/ (Accessed September 08, 2025).

[262] Pei J Pan X Wei G Hua Y . Research progress of glutathione peroxidase family (GPX) in redoxidation. Front Pharmacol. (2023) 14:1147414. doi: 10.3389/FPHAR.2023.1147414 36937839 PMC10017475

[263] Amir Aslani B Ghobadi S . Studies on oxidants and antioxidants with a brief glance at their relevance to the immune system. Life Sci. (2016) 146:163–73. doi: 10.1016/j.lfs.2016.01.014 26792059

[264] Saha SK Lee SB Won J Choi HY Kim K Yang GM . Correlation between oxidative stress, nutrition, and cancer initiation. Int J Mol Sci. (2017) 18(7):1544. doi: 10.3390/IJMS18071544 28714931 PMC5536032

[265] Diamanti-Kandarakis E Papalou O Kandaraki EA Kassi G . Nutrition as a mediator of oxidative stress in metabolic and reproductive disorders in women. Eur J Endocrinol. (2017) 176:R79–99. doi: 10.1530/EJE-16-0616 27678478

[266] Cai Y Yang F Huang X . Oxidative stress and acute pancreatitis (review). BioMed Rep. (2024) 21:124. doi: 10.3892/BR.2024.1812 39006508 PMC11240254

[267] Kandi S Deshpande N Bharath V Pinnelli K Devaki R Rao P . Alcoholism and its role in the development of oxidative stress and DNA damage: An insight. Am J Med Sci Med. (2014) 2:64–6. doi: 10.12691/AJMSM-2-3-3

[268] Osna NA Rasineni K Ganesan M Donohue TM Kharbanda KK . Pathogenesis of alcohol-associated liver disease. J Clin Exp Hepatol. (2022) 12:1492. doi: 10.1016/J.JCEH.2022.05.004 36340300 PMC9630031

[269] Li RL Wang LY Duan HX Zhang Q Guo X Wu C . Regulation of mitochondrial dysfunction induced cell apoptosis is a potential therapeutic strategy for herbal medicine to treat neurodegenerative diseases. Front Pharmacol. (2022) 13:937289. doi: 10.3389/FPHAR.2022.937289/FULL 36210852 PMC9535092

[270] Amador-Licona N Díaz-Murillo TA Gabriel-Ortiz G Pacheco-Moises FP Pereyra-Nobara TA Guízar-Mendoza JM . Omega 3 fatty acids supplementation and oxidative stress in HIV-seropositive patients. A clinical trial. PloS One. (2016) 11(3):e0151637. doi: 10.1371/journal.pone.0151637 27015634 PMC4807787

[271] Cancemi G Cicero N Allegra A Gangemi S . Effect of diet and oxidative stress in the pathogenesis of lymphoproliferative disorders. Antioxidants. (2023) 12:1674. doi: 10.3390/ANTIOX12091674 37759977 PMC10525385

[272] Nance SA Duncan AV Gwathmey TM Hairston KG . Soluble dietary fiber in obesity-associated inflammation and oxidative stress in African American women. FASEB J. (2017) 31:434.2–2. doi: 10.1096/FASEBJ.31.1_SUPPLEMENT.434.2

[273] Usin SG Okon UE Daramola AE Emmanuel PD . Evaluation of phytochemical constituents and elemental profiling of selected medicinal plants in South-West, Nigeria. Asian J Biol Sci. (2024) 17:145–55. doi: 10.3923/AJBS.2024.145.155

[274] Carvalho A Oliveira A Loureiro AM Gattás GJF Fisberg RM Marchioni DM . Arginine intake is associated with oxidative stress in a general population. Nutrition. (2017) 33:211–5. doi: 10.1016/j.nut.2016.07.005 27641673

[275] Bragagna L Maqboul L Baron R Harloff M Spasova M Noori S . A high-protein diet with and without strength training shows no negative effects on oxidative stress markers in older adults. Redox Biol. (2025) 85:103707. doi: 10.1016/J.REDOX.2025.103707 40541063 PMC12219370

[276] Wu S Fisher-Hoch SP Reininger BM Lee M McCormick JB . Fruit and vegetable intake is inversely associated with cancer risk in Mexican-Americans. Nutr Cancer. (2019) 71:1254. doi: 10.1080/01635581.2019.1603315 31017487 PMC7173711

[277] Youssef AMM Maaty DAM Al-Saraireh YM . Phytochemical analysis and profiling of antioxidants and anticancer compounds from Tephrosia purpurea (L.) subsp. apollinea family Fabaceae. Molecules. (2023) 28:3939. doi: 10.3390/MOLECULES28093939 37175349 PMC10180520

[278] Yüksel B Dumlu Bilgin G Kavsara HK . Exploring the relationship between dietary phytochemical index and chemotherapy‐related symptoms: Insights from a cross‐sectional study. Food Sci Nutr. (2024) 12:10306. doi: 10.1002/FSN3.4568 39723091 PMC11666823

[279] Zheng Y Ma Y Xiong Q Zhu K Weng N Zhu Q . The role of artificial intelligence in the development of anticancer therapeutics from natural polyphenols: Current advances and future prospects. Pharmacol Res. (2024) 208:107381. doi: 10.1016/J.PHRS.2024.107381 39218422

[280] Dehelean CA Marcovici I Soica C Mioc M Coricovac D Iurciuc S . Plant-derived anticancer compounds as new perspectives in drug discovery and alternative therapy. Molecules. (2021) 26:1109. doi: 10.3390/MOLECULES26041109 33669817 PMC7922180

[281] Choudhari AS Mandave PC Deshpande M Ranjekar P Prakash O . Phytochemicals in cancer treatment: From preclinical studies to clinical practice. Front Pharmacol. (2020) 10:1614. doi: 10.3389/FPHAR.2019.01614 32116665 PMC7025531

[282] Banudevi S Swaminathan S Maheswari KU . Pleiotropic role of dietary phytochemicals in cancer: Emerging perspectives for combinational therapy. Nutr Cancer. (2015) 67:1021–48. doi: 10.1080/01635581.2015.1073762 26359767

[283] Roszkowski S . Application of polyphenols and flavonoids in oncological therapy. Molecules. (2023) 28:4080. doi: 10.3390/MOLECULES28104080 37241819 PMC10220832

[284] Bhosale PB Ha SE Vetrivel P Kim HH Kim SM Kim GS . Functions of polyphenols and its anticancer properties in biomedical research: a narrative review. Transl Cancer Res. (2020) 9:7619. doi: 10.21037/TCR-20-2359 35117361 PMC8798728

[285] Farhan M Rizvi A . The pharmacological properties of red grape polyphenol resveratrol: Clinical trials and obstacles in drug development. Nutrients. (2023) 15:4486. doi: 10.3390/NU15204486 37892561 PMC10610408

[286] Apostolou A Stagos D Galitsiou E Spyrou A Haroutounian S Portesis N . Assessment of polyphenolic content, antioxidant activity, protection against ROS-induced DNA damage and anticancer activity of Vitis vinifera stem extracts. Food Chem Toxicol. (2013) 61:60–8. doi: 10.1016/j.fct.2013.01.029 23380202

[287] Mileo AM Miccadei S . Polyphenols as modulator of oxidative stress in cancer disease: New therapeutic strategies. Oxid Med Cell Longev. (2015) 2016:6475624. doi: 10.1155/2016/6475624 26649142 PMC4663347

[288] Greenwell M Rahman PKSM . Medicinal plants: Their use in anticancer treatment. Int J Pharm Sci Res. (2015) 6:4103–12. doi: 10.13040/IJPSR.0975-8232.6(10).4103-12 26594645 PMC4650206

[289] Sichetti M Giuseffi M Giglio E Marino G Mecca M . Effect of natural polyphenols on breast cancer chemoprevention and treatment. Mol Nutr Food Res. (2025) 69:e70055. doi: 10.1002/MNFR.70055 40190185 PMC12371390

[290] Rudrapal M Khairnar SJ Khan J Dukhyil AB Ansari MA Alomary MN . Dietary polyphenols and their role in oxidative stress-induced human diseases: Insights into protective effects, antioxidant potentials and mechanism(s) of action. Front Pharmacol. (2022) 13:806470. doi: 10.3389/FPHAR.2022.806470 35237163 PMC8882865

[291] Khanvilkar S Mittra I . Copper imparts a new therapeutic property to resveratrol by generating ROS to deactivate cell-free chromatin. Pharmaceuticals. (2025) 18:132. doi: 10.3390/PH18010132/S1 39861193 PMC11769567

[292] Ullah A Munir S Badshah SL Khan N Ghani L Poulson BG . Important flavonoids and their role as a therapeutic agent. Molecules. (2020) 25:5243. doi: 10.3390/MOLECULES25225243 33187049 PMC7697716

[293] Safe S Jayaraman A Chapkin RS Howard M Mohankumar K Shrestha R . Flavonoids: structure–function and mechanisms of action and opportunities for drug development. Toxicol Res. (2021) 37:147. doi: 10.1007/S43188-020-00080-Z 33868973 PMC8007671

[294] Cao J Xia X Chen X Xiao J Wang Q . Characterization of flavonoids from Dryopteris erythrosora and evaluation of their antioxidant, anticancer and acetylcholinesterase inhibition activities. Food Chem Toxicol. (2013) 51:242–50. doi: 10.1016/j.fct.2012.09.039 23063594

[295] Batra P Sharma AK . Anti-cancer potential of flavonoids: recent trends and future perspectives. 3 Biotech. (2013) 3:439. doi: 10.1007/S13205-013-0117-5 28324424 PMC3824783

[296] Wen L Wu D Jiang Y Prasad KN Lin S Jiang G . Identification of flavonoids in litchi (Litchi chinensis Sonn.) leaf and evaluation of anticancer activities. J Funct Foods. (2009) 6:555–63. doi: 10.1016/J.JFF.2013.11.022 38826717

[297] Hu S Xu Y Meng L Huang L Sun H . Curcumin inhibits proliferation and promotes apoptosis of breast cancer cells. Exp Ther Med. (2018) 16:1266. doi: 10.3892/ETM.2018.6345 30116377 PMC6090267

[298] Karimi M Parsania M Motakef Kazemi N Qomi M Hadipour Jahromy M . Curcumin nanoemulsion suppresses HPV oncogenes and inhibits cervical cancer progression: *in vitro* and *in vivo* study. Virol J. (2025) 22:1–8. doi: 10.1186/S12985-025-02738-2 40426123 PMC12117926

[299] Endo H Inoue I Masunaka K Tanaka M Yano M . Curcumin induces apoptosis in lung cancer cells by 14-3-3 protein-mediated activation of Bad. Biosci Biotechnol Biochem. (2020) 84:2440–7. doi: 10.1080/09168451.2020.1808443 32841581

[300] Kvasnica M Buchtova K Budesinsky M Beres T Rarova L Strnad M . Synthesis, characterization and antiproliferative activity of seco analogues of brassinosteroids. Steroids. (2019) 146:1–13. doi: 10.1016/J.STEROIDS.2019.03.004 30885649

[301] Malíková J Swaczynová J Kolář Z Strnad M . Anticancer and antiproliferative activity of natural brassinosteroids. Phytochemistry. (2008) 69:418–26. doi: 10.1016/J.PHYTOCHEM.2007.07.028 17869317

[302] Steigerová J Oklestkova J Levková M Rárová L Kolář Z Strnad M . Brassinosteroids cause cell cycle arrest and apoptosis of human breast cancer cells. Chem Biol Interact. (2010) 188:487–96. doi: 10.1016/j.cbi.2010.09.006 20833159

[303] Steigerová J Rárová L Oklešt’ková J Křížová K Levková M Šváchová M . Mechanisms of natural brassinosteroid-induced apoptosis of prostate cancer cells. Food Chem Toxicol. (2012) 50:4068–76. doi: 10.1016/j.fct.2012.08.031 22939933

[304] Griñan-Lison C Blaya-Cánovas JL López-Tejada A Ávalos-Moreno M Navarro-Ocón A Cara FE . Antioxidants for the treatment of breast cancer: Are we there yet? Antioxidants. (2021) 10:205. doi: 10.3390/antiox10020205 33572626 PMC7911462

[305] Ávila‐Gálvez MÁ González‐Sarrías A Martínez‐Díaz F Abellán B Martínez‐Torrano AJ Fernández‐López AJ . Disposition of dietary polyphenols in breast cancer patients’ tumors, and their associated anticancer activity: The particular case of curcumin. Mol Nutr Food Res. (2021) 65(12):e2100163. doi: 10.1002/mnfr.202100163 33939887

[306] Barcelos KA Mendonça CR Noll M Botelho AF Francischini CRD Silva MAM . Antitumor properties of curcumin in breast cancer based on preclinical studies: A systematic review. Cancers (Basel). (2022) 14(9):2165. doi: 10.3390/cancers14092165 35565294 PMC9099919

[307] Wu W Kang S Zhang D . Association of vitamin B6, vitamin B12 and methionine with risk of breast cancer: a dose-response meta-analysis. Br J Cancer. (2013) 109:1926–44. doi: 10.1038/bjc.2013.438 23907430 PMC3790153

[308] Lee SJ Jeong J-H Lee IH Lee J Jung JH Park HY . Effect of high-dose vitamin C combined with anti-cancer treatment on breast cancer cells. Anticancer Res. (2019) 39:751–8. doi: 10.21873/anticanres.13172 30711954

[309] Codini M . Why vitamin C could be an excellent complementary remedy to conventional therapies for breast cancer. Int J Mol Sci. (2020) 21:8397. doi: 10.3390/ijms21218397 33182353 PMC7664876

[310] González-Montero J Chichiarelli S Eufemi M Altieri F Saso L Rodrigo R . Ascorbate as a bioactive compound in cancer therapy: The old classic strikes back. Molecules. (2022) 27:3818. doi: 10.3390/molecules27123818 35744943 PMC9229419

[311] Harris HR Orsini N Wolk A . Vitamin C and survival among women with breast cancer: A meta-analysis. Eur J Cancer. (2014) 50:1223–31. doi: 10.1016/j.ejca.2014.02.013 24613622

[312] Zhang D Xu P Li Y Wei B Yang S Zheng Y . Association of vitamin C intake with breast cancer risk and mortality: a meta-analysis of observational studies. Aging (Albany NY). (2020) 12:18415. doi: 10.18632/AGING.103769 32991322 PMC7585084

[313] Shamsi U Khan S Azam I Habib Khan A Maqbool A Hanif M . A multicenter case control study of association of vitamin D with breast cancer among women in Karachi, Pakistan. PloS One. (2020) 15:e0225402. doi: 10.1371/journal.pone.0225402 31967989 PMC6975526

[314] Li Z Wu L Zhang J Huang X Thabane L Li G . Effect of vitamin D supplementation on risk of breast cancer: A systematic review and meta-analysis of randomized controlled trials. Front Nutr. (2021) 8. doi: 10.3389/fnut.2021.655727 33869269 PMC8049142

[315] Peila R Xue X Cauley JA Chlebowski R Manson JE Nassir R . A randomized trial of calcium plus vitamin D supplementation and risk of ductal carcinoma in situ of the breast. JNCI Cancer Spectr. (2021) 5(4):pkab072. doi: 10.1093/jncics/pkab072 34476342 PMC8406436

[316] Ogunleye AA Xue F Michels KB . Green tea consumption and breast cancer risk or recurrence: a meta-analysis. Breast Cancer Res Treat. (2010) 119:477–84. doi: 10.1007/s10549-009-0415-0 19437116

[317] Wang Y Zhao Y Chong F Song M Sun Q Li T . A dose-response meta-analysis of green tea consumption and breast cancer risk. Int J Food Sci Nutr. (2020) 71:656–67. doi: 10.1080/09637486.2020.1715353 31959020

[318] Moustafa I Connolly C Anis M Mustafa H Oosthuizen F Viljoen M . A prospective study to evaluate the efficacy and safety of vitamin E and levocarnitine prophylaxis against doxorubicin-induced cardiotoxicity in adult breast cancer patients. J Oncol Pharm Pract. (2024) 30:354–66. doi: 10.1177/10781552231171114 37157803

[319] Afsar T Razak S Almajwal A Al-Disi D . Doxorubicin-induced alterations in kidney functioning, oxidative stress, DNA damage, and renal tissue morphology; Improvement by Acacia hydaspica tannin-rich ethyl acetate fraction. Saudi J Biol Sci. (2020) 27:2251–60. doi: 10.1016/j.sjbs.2020.07.011 32884406 PMC7451730

[320] Kim SJ Zhang CXW Demsky R Armel S Kim Y-I Narod SA . Folic acid supplement use and breast cancer risk in BRCA1 and BRCA2 mutation carriers: a case-control study. Breast Cancer Res Treat. (2019) 174:741–8. doi: 10.1007/s10549-018-05118-3 30603998

[321] Zeng J Wang K Ye F Lei L Zhou Y Chen J . Folate intake and the risk of breast cancer: an up-to-date meta-analysis of prospective studies. Eur J Clin Nutr. (2019) 73:1657–60. doi: 10.1038/s41430-019-0394-0 30647438

[322] Maroof H Hassan ZM Mobarez AM Mohamadabadi MA . Lactobacillus acidophilus could modulate the immune response against breast cancer in murine model. J ClinImmunol. (2012) 32:1353–9. doi: 10.1007/s10875-012-9708-x 22711009

[323] German R Marino N Hemmerich C Podicheti R Rusch DB Stiemsma LT . Exploring breast tissue microbial composition and the association with breast cancer risk factors. Breast Cancer Res. (2023) 25:82. doi: 10.1186/s13058-023-01677-6 37430354 PMC10332106

[324] Zou L . Significant role of antioxidants in the treatment of breast cancer. Oxid Antioxid Med Sci. (2022) 11(6):1. doi: 10.5455/oams.220622.gt02

[325] Eliassen AH Hendrickson SJ Brinton LA Buring JE Campos H Dai Q . Circulating carotenoids and risk of breast cancer: Pooled analysis of eight prospective studies. JNCI: J Natl Cancer Institute. (2012) 104:1905–16. doi: 10.1093/jnci/djs461 23221879 PMC3525817

[326] Gloria NF Soares N Brand C Oliveira FL Borojevic R Teodoro AJ . Lycopene and beta-carotene induce cell-cycle arrest and apoptosis in human breast cancer cell lines. Anticancer Res. (2014) 34:1377–86. 24596385

[327] He J Gu Y Zhang S . Vitamin A and breast cancer survival: A systematic review and meta-analysis. Clin Breast Cancer. (2018) 18:e1389–400. doi: 10.1016/j.clbc.2018.07.025 30190194

[328] Herdiana Y Sriwidodo S Sofian FF Wilar G Diantini A . Nanoparticle-based antioxidants in stress signaling and programmed cell death in breast cancer treatment. Molecules. (2023) 28:5305. doi: 10.3390/molecules28145305 37513179 PMC10384004

[329] Atwell LL Beaver LM Shannon J Williams DE Dashwood RH Ho E . Epigenetic regulation by sulforaphane: Opportunities for breast and prostate cancer chemoprevention. Curr Pharmacol Rep. (2015) 1:102–11. doi: 10.1007/s40495-014-0002-x 26042194 PMC4450146

[330] Li Y Buckhaults P Li S Tollefsbol T . Temporal efficacy of a sulforaphane-based broccoli sprout diet in prevention of breast cancer through modulation of epigenetic mechanisms. Cancer Prev Res. (2018) 11:451–64. doi: 10.1158/1940-6207.CAPR-17-0423 29764806 PMC6072582

[331] Rahman KW Aranha OP Sarkar FH . Indole-3-carbinol (I3C) induces apoptosis in tumorigenic but not in nontumorigenic breast epithelial cells. Nutr Cancer. (2003) 45:101–12. doi: 10.1207/S15327914NC4501_12 12791510

[332] Liu X Lv K . Cruciferous vegetables intake is inversely associated with risk of breast cancer: A meta-analysis. Breast. (2013) 22:309–13. doi: 10.1016/j.breast.2012.07.013 22877795

[333] Alqarni AA Alamoudi AA Allam RM Ajabnoor GM Harakeh SM Al-Abd AM . The influence of antioxidant dietary-derived polyphenolic combination on breast cancer: Molecular study. Biomedicine Pharmacotherapy. (2022) 149:112835. doi: 10.1016/j.biopha.2022.112835 35325850

[334] Smeriglio A Iraci N Denaro M Mandalari G Giofrè SV Trombetta D . Synergistic combination of citrus flavanones as strong antioxidant and COX-inhibitor agent. Antioxidants. (2023) 12:972. doi: 10.3390/antiox12040972 37107347 PMC10136195

[335] Rossi M Bosetti C Negri E Lagiou P Vecchia CL . Flavonoids, proanthocyanidins, and cancer risk: A network of case-control studies from Italy. Nutr Cancer. (2010) 62:871–7. doi: 10.1080/01635581.2010.509534 20924962

[336] Hui C Qi X Qianyong Z Xiaoli P Jundong Z Mantian M . Flavonoids, flavonoid subclasses and breast cancer risk: A meta-analysis of epidemiologic studies. PloS One. (2013) 8:e54318. doi: 10.1371/journal.pone.0054318 23349849 PMC3548848

[337] Liu J Li X Hou J Sun J Guo N Wang Z . Dietary intake of n-3 and n-6 polyunsaturated fatty acids and risk of cancer: Meta-analysis of data from 32 studies. Nutr Cancer. (2021) 73:901–13. doi: 10.1080/01635581.2020.1779321 32530319

[338] Zhang Z-L Ho SC Liu K-Y Mo X-F Feng X-L Li L . Association of dietary intake of n-3 polyunsaturated fatty acids with breast cancer risk in pre- and postmenopausal Chinese women. Menopause. (2022) 29:932–43. doi: 10.1097/GME.0000000000001990 35881925

[339] Benhar M . Oxidants, antioxidants and thiol redox switches in the control of regulated cell death pathways. Antioxidants. (2020) 9:309. doi: 10.3390/antiox9040309 32290499 PMC7222211

[340] Kapała A Szlendak M Motacka E . The anti-cancer activity of lycopene: A systematic review of human and animal studies. Nutrients. (2022) 14:5152. doi: 10.3390/nu14235152 36501182 PMC9741066

[341] Dehnavi MK Ebrahimpour-Koujan S Lotfi K Azadbakht L . The association between circulating carotenoids and risk of breast cancer: A systematic review and dose–response meta-analysis of prospective studies. Adv Nutr. (2024) 15:100135. doi: 10.1016/j.advnut.2023.10.007 38436219 PMC10694674

[342] Zheng J Zhou Y Li Y Xu D-P Li S Li H-B . Spices for prevention and treatment of cancers. Nutrients. (2016) 8:495. doi: 10.3390/nu8080495 27529277 PMC4997408

[343] Cevatemre B Erkısa M Aztopal N Karakas D Alper P Tsimplouli C . A promising natural product, pristimerin, results in cytotoxicity against breast cancer stem cells *in vitro* and xenografts *in vivo* through apoptosis and an incomplete autopaghy in breast cancer. Pharmacol Res. (2018) 129:500–14. doi: 10.1016/J.PHRS.2017.11.027 29197639

[344] Lin J Cook NR Albert C Zaharris E Gaziano JM Van Denburgh M . Vitamins C and E and beta carotene supplementation and cancer risk: A randomized controlled trial. JNCI J Natl Cancer Institute. (2009) 101:14–23. doi: 10.1093/jnci/djn438 19116389 PMC2615459

[345] Bardia A Tleyjeh IM Cerhan JR Sood AK Limburg PJ Erwin PJ . Efficacy of antioxidant supplementation in reducing primary cancer incidence and mortality: Systematic review and meta-analysis. Mayo Clin Proc. (2008) 83:23–34. doi: 10.4065/83.1.23 18173999

[346] Manson JE Crandall CJ Rossouw JE Chlebowski RT Anderson GL Stefanick ML . The Women’s Health Initiative randomized trials and clinical practice. Jama. (2024) 331:1748. doi: 10.1001/jama.2024.6542 38691368

[347] Hercberg S Galan P Preziosi P Bertrais S Mennen L Malvy D . The SU.VI.MAX study. Arch Intern Med. (2004) 164:2335. doi: 10.1001/archinte.164.21.2335 15557412

[348] Ambrosone CB Zirpoli GR Hutson AD McCann WE McCann SE Barlow WE . Dietary supplement use during chemotherapy and survival outcomes of patients with breast cancer enrolled in a cooperative group clinical trial (SWOG S0221). J Clin Oncol. (2020) 38:804–14. doi: 10.1200/JCO.19.01203 31855498 PMC7062457

[349] Özkurt E Ordu C Özmen T Ilgun AS Soybir G Celebi F . Vitamin D supplementation during neoadjuvant chemotherapy for breast cancer improves pathological complete response: A prospective randomized clinical trial. World J Surg. (2025) 49:1396–405. doi: 10.1002/wjs.12587 40229998 PMC12134193

[350] Barton DL Loprinzi CL Quella SK Sloan JA Veeder MH Egner JR . Prospective evaluation of vitamin E for hot flashes in breast cancer survivors. J Clin Oncol. (1998) 16:495–500. doi: 10.1200/JCO.1998.16.2.495 9469333

[351] de Souza Fêde ÂB Bensi CG Trufelli DC de Oliveira Campos MP Pecoroni PG Ranzatti RP . Multivitamins do not improve radiation therapy-related fatigue. Am J Clin Oncol. (2007) 30:432–6. doi: 10.1097/COC.0b013e31804b40d9 17762445

[352] Lesser GJ Case D Stark N Williford S Giguere J Garino LA . A randomized, double-blind, placebo-controlled study of oral coenzyme Q10 to relieve self-reported treatment-related fatigue in newly diagnosed patients with breast cancer. J Support Oncol. (2012) 11(1):31–42. doi: 10.1016/j.suponc.2012.03.003 22682875 PMC3501550

[353] Gulcin İ . Antioxidants: a comprehensive review. Arch Toxicol. (2025) 99:1893. doi: 10.1007/S00204-025-03997-2 40232392 PMC12085410

[354] Averill-Bates DA . The antioxidant glutathione. Vitam Horm. (2023) 121:109–41. doi: 10.1016/BS.VH.2022.09.002 36707132

[355] Kozlov AV Javadov S Sommer N . Cellular ROS and antioxidants: physiological and pathological role. Antioxidants. (2024) 13:602. doi: 10.3390/ANTIOX13050602 38790707 PMC11117742

[356] Siddeeg A AlKehayez NM Abu-Hiamed HA Al-Sanea EA AL-Farga AM . Mode of action and determination of antioxidant activity in the dietary sources: An overview. Saudi J Biol Sci. (2021) 28:1633–44. doi: 10.1016/J.SJBS.2020.11.064 33732049 PMC7938136

[357] Archibong AE Rideout ML Harris KJ Ramesh A . Oxidative stress in reproductive toxicology. Curr Opin Toxicol. (2018) 7:95–101. doi: 10.1016/J.COTOX.2017.10.004 30105313 PMC6086129

[358] Yang Y Ling W . Health benefits and future research of phytochemicals: A literature review. J Nutr. (2025) 155:87–101. doi: 10.1016/J.TJNUT.2024.11.007 39536969

[359] Landis-Piwowar KR Iyer NR . Cancer chemoprevention: current state of the art. Cancer Growth Metastasis. (2014) 7:CGM.S11288. doi: 10.4137/CGM.S11288 24987270 PMC4064948

[360] Steward WP Brown K . Cancer chemoprevention: a rapidly evolving field. Br J Cancer. (2013) 109:1–7. doi: 10.1038/bjc.2013.280 23736035 PMC3708589

[361] Usman M Khan WR Yousaf N Akram S Murtaza G Kudus KA . Exploring the phytochemicals and anti-cancer potential of the members of Fabaceae family: A comprehensive review. Molecules. (2022) 27:3863. doi: 10.3390/molecules27123863 35744986 PMC9230627

[362] Ferrante A Tamma M Agriesti F Tucci F Lopriore P Amodio ML . Characterization of the effect of pomegranate crude extract, and its post-harvesting preservation procedures, on redox tone, cellular growth and metabolic profile of MDA-MB-231 cell line. BMC Complement Med Ther. (2023) 23:311. doi: 10.1186/s12906-023-04134-1 37684643 PMC10485948

[363] Díaz-Mula HM Tomás-Barberán FA García-Villalba R . Pomegranate fruit and juice (cv. Mollar), rich in ellagitannins and anthocyanins, also provide a significant content of a wide range of proanthocyanidins. J Agric Food Chem. (2019) 67:9160–7. doi: 10.1021/ACS.JAFC.8B07155 30768267

[364] Sharma P McClees SF Afaq F . Pomegranate for prevention and treatment of cancer: An update. Molecules. (2017) 22(1):E177. doi: 10.3390/molecules22010177 28125044 PMC5560105

[365] Syed DN Chamcheu J-C Adhami VM Mukhtar H . Pomegranate extracts and cancer prevention: Molecular and cellular activities. Anti-Cancer Agents Med Chem. (2013) 13:1149. doi: 10.2174/1871520611313080003 23094914 PMC4052369

[366] Rahman MM Islam MR Akash S Hossain ME Tumpa AA Abrar Ishtiaque GM . Pomegranate-specific natural compounds as onco-preventive and onco-therapeutic compounds: Comparison with conventional drugs acting on the same molecular mechanisms. Heliyon. (2023) 9:e18090. doi: 10.1016/J.HELIYON.2023.E18090 37519687 PMC10372646

[367] Rauf A Olatunde A Akram Z Hemeg HA Aljohani ASM Al Abdulmonem W . The role of pomegranate (Punica granatum) in cancer prevention and treatment: Modulating signaling pathways from inflammation to metastasis. Food Sci Nutr. (2025) 13:e4674. doi: 10.1002/FSN3.4674 39898127 PMC11782917

[368] Caruso A Barbarossa A Tassone A Ceramella J Carocci A Catalano A . Pomegranate: nutraceutical with promising benefits on human health. Appl Sci. (2020) 10:6915. doi: 10.3390/APP10196915 30654563

[369] Rocha A Wang L Penichet M Martins-Green M . Pomegranate juice and specific components inhibit cell and molecular processes critical for metastasis of breast cancer. Breast Cancer Res Treat. (2012) 136:647–58. doi: 10.1007/S10549-012-2264-5 23065001

[370] Eroglu Ozkan E Seyhan MF Kurt Sirin O Yilmaz- Ozden T Ersoy E Hatipoglu Cakmar SD . Antiproliferative effects of Turkish pomegranate (*Punica granatum* L.) extracts on MCF-7 human breast cancer cell lines with focus on antioxidant potential and bioactive compounds analyzed by LC-MS/MS. J Food Biochem. (2021) 45(9):e13904. doi: 10.1111/JFBC.13904 34414576

[371] Kuban-Jankowska A Kostrzewa T Musial C Barone G Lo-Bosco G Lo-Celso F . Green tea catechins induce inhibition of PTP1B phosphatase in breast cancer cells with potent anti-cancer properties: *In vitro* assay, molecular docking, and dynamics studies. Antioxidants. (2020) 9:1208. doi: 10.3390/antiox9121208 33266280 PMC7761018

[372] Chen D Guo Z Yao L Sun Y Dian Y Zhao D . Targeting oxidative stress-mediated regulated cell death as a vulnerability in cancer. Redox Biol. (2025) 84:103686. doi: 10.1016/J.REDOX.2025.103686 40424719 PMC12159232

[373] Didier AJ Stiene J Fang L Watkins D Dworkin LD Creeden JF . Antioxidant and anti-tumor effects of dietary vitamins A, C, and E. Antioxidants. (2023) 12:632. doi: 10.3390/ANTIOX12030632 36978880 PMC10045152

[374] Bhatnagar P Pant AB Shukla Y Chaudhari B Kumar P Gupta KC . Bromelain nanoparticles protect against 7,12-dimethylbenz[a]anthracene induced skin carcinogenesis in mouse model. Eur J Pharm Biopharm. (2015) 91:35–46. doi: 10.1016/J.EJPB.2015.01.015 25619920

[375] Ban C Jo M Park YH Kim JH Han JY Lee KW . Enhancing the oral bioavailability of curcumin using solid lipid nanoparticles. Food Chem. (2020) 302:125328. doi: 10.1016/j.foodchem.2019.125328 31404868

